# CDH1 overexpression predicts bladder cancer from early stage and inversely correlates with immune infiltration

**DOI:** 10.1186/s12894-022-01103-7

**Published:** 2022-09-21

**Authors:** Tao Fan, Liang Xue, Bingzheng Dong, Houguang He, Wenda Zhang, Lin Hao, Weiming Ma, Guanghui Zang, Conghui Han, Yang Dong

**Affiliations:** 1 Department of Clinical Medicine, Xuzhou Medical University, Xuzhou, China; 2 Department of Urology, Xuzhou Central Hospital, Jiefang South Road, No. 199, Xuzhou, Jiangsu China; 3 Medical College of Soochow University, Soochow, China

**Keywords:** Bladder cancer, Biomarker, CDH1, Methylation, Immune infiltration

## Abstract

**Background:**

Bladder cancer (BC) seriously endangers public health, but effective biomarkers for BC diagnosis, particularly in the early stage, are still lacking. Identification of reliable biomarkers associated with early-stage BC is of great importance to early treatment and an improved outcome.

**Methods:**

Differentially expressed genes (DEGs) were identified using four publicly available early-stage BC gene-expression profiles. Protein–protein interaction (PPI) and survival analysis for hub genes was evaluated. The correlation between methylation of genes and prognosis was evaluated using the MethSurv database. Co-expressed genes were explored using Cancer Cell Line Encyclopedia database and the corresponding expression were assessed in vitro. The competing endogenous RNA network and the immune cell infiltration in BC were generated using data of The Cancer Genome Atlas.

**Results:**

Ten hub genes of the 213 integrated DEGs were identified, including *CDH1*, *IGFBP3*, *PPARG*, *SDC1*, *EPCAM*, *ACTA2*, *COL3A1*, *TPM1*, *ACTC1*, and *ACTN1*. *CDH1* appeared to increase from tumor initiation stage and negatively correlated with methylation. Six methylated sites in *CDH1* indicated a good prognosis and one site indicated an aberrant prognosis. High *CDH1* expression was negatively correlated with infiltrations by most immune cells, such as plasmacytoid dendritic cells (pDCs), regulatory T cells, macrophages, neutrophils, DCs, and natural killer cells. *CDH1* was highly positively correlated with *EPCAM* and appeared to be directly regulated by miR-383.

**Conclusions:**

The identified oncogenic alterations provide theoretical support for the development of novel biomarkers to advance early-stage BC diagnosis and personalized therapy.

**Supplementary Information:**

The online version contains supplementary material available at 10.1186/s12894-022-01103-7.

## Background

Bladder cancer (BC) is one of the most common malignancies worldwide. For decades, BC has had the leading incidence and mortality rates among cancers of the genitourinary system in China [1], posing a serious threat to human health. Among the patients with bladder malignancies, approximately 75% of the diagnoses are related to non–muscle-invasive bladder cancer (NMIBC) at initial presentation [2]. NMIBC is more common in patients under 40 years of age [3], and although patients can be effectively treated by transurethral resection of the bladder tumor and postoperative bladder perfusion chemotherapy [4], NMIBC regularly becomes recurrent, causing fatigue in most patients. In approximately 30% of the patients, NMIBC eventually progresses to muscle-invasive bladder cancer (MIBC) within 5 years [5]. Patients with MIBC have a poor prognosis with a 5-year survival rate of approximately 50% [6].

For patients with BC in the early stage, effective early detection is the key to improving the cure rate and preventing progression to muscle invasion. Currently, cystoscopy and urine cytology are the gold standard for diagnosing BC. However, since relatively frequent cystoscopy involves an invasive examination, it presents a great challenge to the patients’ physical and mental health. Moreover, the sensitivity of urine cytology to low-grade tumors is low [7]. Therefore, more efficient, accurate, and less invasive examination methods have been pursued, and the identification of novel biomarkers for BC has attracted considerable research attention in recent years.

Recently, an increasing number of microarray and high throughput sequencing technologies have been developed to identify biomarkers associated with malignancy, which enable the early diagnosis, prognosis, recurrence monitoring, and exploration of novel drug targets [8]. Integrated bioinformatics technologies have proved effective in overcoming inconsistent results obtained from different platforms and limited cancer sample sizes, facilitating the discovery of a wealth of valuable biological insights [9].

In this study, we identified differentially expressed genes (DEGs) in four raw gene chip expression profile datasets downloaded from the Gene Expression Omnibus (GEO) database, including 29 normal bladder tissue samples and 39 early-stage BC samples (Ta and T1 stage). Gene ontology (GO) and Kyoto Encyclopedia of Genes and Genomes (KEGG) pathway enrichment analyses were performed. A protein–protein interaction (PPI) network was constructed to identify the final hub genes, and the expression of individual genes was assessed by quantitative reverse-transcription polymerase chain reaction (qRT-PCR). The association of specific gene expressions with clinicopathological characteristics and immune infiltration and the correlation between the specific gene methylation and prognosis in BC were further analyzed. Finally, the potential regulators of hub genes were evaluated using a competing endogenous RNA (ceRNA) network. The present study provides promising new insights into potentially reliable biomarkers for tumorigenesis of bladder cancer.

## Methods

### Microarray data

The GEO database (http://www.ncbi.nlm.nih.gov/geo) [10] is a public genomic data repository that allows users to access high-throughput gene expression data submitted by global research institutions. Four gene expression datasets containing human NMIBC and corresponding adjacent normal tissues [GSE3167 [11], GSE7476 [12], GSE40355 [13], and GSE65635 [14]] were downloaded from GEO. The annotation information on the platform was used as a reference for the corresponding gene symbols. The clinical information of patients with BC and their corresponding mRNA, lncRNA, and miRNA expression data were downloaded from The Cancer Genome Atlas (TCGA) website (https://cancergenome.nih.gov/), as of August 15, 2020. The expression of hub genes in BC and normal bladder samples was evaluated and visualized by the Gene Expression Profiling Interactive Analysis (GEPIA) platform (http://gepia.cancer-pku.cn) [15]. Additionally, RNA-Seq data of candidate genes in different urinary tract cancer cell lines (*n* = 25) were extracted from the Cancer Cell Line Encyclopedia (CCLE) database (https://portals.broadinstitute.org/ccle/about) [16], as of August 20, 2020.

### Integrated analysis of microarray datasets

The limma package [17] in the R/Bioconductor software was used to normalize the matrix data, perform Log2 conversion, and identify DEGs in each microarray dataset. DEGs were integrated using the RobustRankAggreg (RRA) method [18], assuming that each gene ranked randomly in each dataset. If the gene ranked higher across all datasets, the associated *P*-value was then lower, and the possibility of differential gene expression was greater. A difference in gene expression was considered significant if | Log2FC (fold change) |≥ 1 and adjusted *P*-value < 0.05.

### Function and pathway enrichment analysis

We used the DOSE [19] and clusterProfiler [20] packages of the statistical software R (Version 3.6.2) for mining information related to the biological effects of DEGs and for implementing GO classification and KEGG pathway enrichment [21–23]. High quality graphs were displayed using the ggplot2 and pROC packages. Gene set enrichment analysis (GSEA) is a computational method that determines whether an a priori defined set of genes shows significant, concordant differences between two biological states [24]. Gene set enrichment was analyzed using GSEA (version 4.0.3). The functional gene set file ‘c2.cp.kegg.v7.0.symbols.gmt’ summarizes specific and well-defined signaling. The number of substitutions per analysis was set at 1,000, and gene sets with *P* < 0.05 were recognized as significantly enriched.

### PPI network construction and module analysis

An initial PPI network was constructed using the Search Tool for the Retrieval of Interacting Genes (STRING) (version 11.0; http://string-db.org) platform [25]. The minimum value for the highest confidence was set to 0.7, and unconnected proteins were removed from the network. A given network was clustered based on topology using Molecular Complex Detection (MCODE) (version 1.4.2), a plugin of Cytoscape (version 3.4.0), to identify densely connected regions [26]. The final PPI networks were mapped using the Cytoscape visualization software, and the most significant module according to MCODE was identified. The data were filtered based on the following criteria: MCODE score > 5, maximum depth = 100, node score cut-off = 0.2, degree cut-off = 2, and *k*-score = 2.

### Cell culture

The human BC cell lines 5637 and RT4 and the normal urothelial cell line SVHUC1 were purchased from the Cell Resource Center of the Shanghai Institutes for Biological Sciences, Chinese Academy of Sciences (Shanghai, China). All cell lines were cultured in RPMI 1640 medium with 100 U/mL penicillin, 100 μg/mL streptomycin, and 10% fetal bovine serum at 5% CO_2_ in a 37 ℃ humidified culture environment. Short-tandem repeat profiling was used to authenticate the cell lines less than 6 months before this project was initiated, and the cells were not in culture for more than 2 months.

### Analysis of *CDH1* methylation and prognosis

*CDH1* methylation data was obtained from the cBioPortal (https://www.cbioportal.org/) platform. The correlation between *CDH1* methylation and expression level was tested by Spearman correlation analysis and visualized in the cBioPortal platform. Moreover, the prognostic value of the *CDH1* methylation level in BC and identification of methylation sites associated with prognosis were evaluated using the MethSurv database (https://biit.cs.ut.ee/methsurv/) [27], which provides a visualization tool to perform survival analysis based on the DNA methylation level of specific genes using TCGA-BLCA data.

### Immune infiltration analysis

Immune infiltration levels were evaluated by single-sample GSEA (ssGSEA) using the “GSVA” R package, which can determine the immune cell population in a tumor sample according to gene expression data [28]. The infiltration enrichment of 24 common immune cell types was computed, including B cells, cytotoxic cells, dendritic cells (DCs), activated DCs (aDCs), immature DCs (iDCs), plasmacytoid DCs (pDCs), eosinophils, macrophages, mast cells, neutrophils, natural killer (NK) cells, NK 56- cells, and NK 56 + cells, T cells, CD8 + T cells, T central memory cells (Tcm), T effector memory cells (Tem), T follicular helper cells (Tfh), T gamma delta cells (Tgd), T helper cells (Th), regulatory T cells (Treg), type 1 Th cells (Th1), type 2 Th cells (Th2), and type 17 Th cells (Th17). The correlation between *CDH1* expression and immune cell infiltration in BC was evaluated by Spearman rank correlation analysis. The ggplot2 package in the R language was used to show plots of immune cell types. The infiltration level in each immune cell was compared between low and high *CDH1* expression groups via Wilcoxon rank sum tests.

### RNA isolation and qRT-PCR

Total RNA from each cell line was isolated using TRIzol reagent (Life Technologies, Carlsbad, CA, USA) according to the manufacturer’s instructions. qRT-PCR was performed using the SYBR Premix Ex Taq II (Perfect Real Time) kit (TaKaRa Bio, Shiga, Japan), with the following settings: 95 °C for 30 s and 39 cycles of 95 ℃ for 5 s and 60 °C for 30 s. DNA dissociation analysis (melting curve) was performed at the end of each run to detect primer dimers, mixed-amplicon populations, and nonspecific products. The relative expression of genes was presented as comparative threshold cycle (2^−ΔΔCt^) values from at least three independent experiments. The expression of target genes was standardized against Actin Beta (*β-actin*). The following primer sequences were used: Human *CDH1*, forward 5′-GAGGCTAACGTCGTAATCACCACA-3′ and reverse 5′-CAAATTGTCCACCATCATCATTCAA-3′; human *EPCAM*, forward 5′-TGCCAGTGTACTTCAGTTGGT-3′ and reverse 5′-AAAGCCCATCATTGTTCTGGA-3′; and *β-actin*, forward 5′-AAACGTGCTGCTGACCGAG-3′ and reverse 5′-TAGCACAGCCTGGATAGCAAC-3′.

### The ceRNA network construction

To characterize potential regulators of hub genes, a ceRNA network was established. Potential miRNA associated with hub genes was first identified via the TargetScan (http://www.targetscan.org/) and miRDB (http://www.mirdb.org/mmiRDB/) databases. Then, to ensure the quantity of obtained data and matches, differentially expressed lncRNAs (DElncRNAs) (| Log_2_FC |> 2.0) and DEmiRNA (| Log_2_FC |> 1.0) with an adjusted *P* < 0.05 were identified using the edgeR package in R software. Next, miRNAs related to DElncRNAs were predicted using miRcode (http://www.mircode.org/), and miRNAs irrelevant to the hub genes were removed from the network. Finally, these data were integrated, and the ceRNA network was visualized using Cytoscape.

### Statistical analysis

The chi-square test was used to analyze the relationship between gene expression and clinical data. The Kaplan–Meier curve and log-rank test were used to plot survival curves. Univariate Cox analysis was used to select relevant variables, and subsequently, multivariate Cox analysis was used for prognostic analysis of gene expression relative to the overall survival (OS) rate of patients with BC. Using the expression level of each mRNA and the regression coefficient obtained from multivariate Cox analysis, a risk score was calculated using the function, Risk score = ExpmRNA1 × βmRNA1 + ExpmRNA2 × βmRNA2 + … + ExpmRNAn × βmRNAn, where Exp represents the expression level of each mRNA and β represents the regression coefficient of each mRNA. An optimal risk model was generated based on the Akaike Information Criterion (AIC) [29]. Patients were divided by median value of risk scores into high-risk and low-risk groups. The predicted power of the prognostic gene signature was determined by the area under the curve (AUC) of the receiver operating characteristic (ROC) curves. *P* < 0.05 was considered statistically significant.

## Results

### Identification and integration of DEGs

**Fig. 1:**
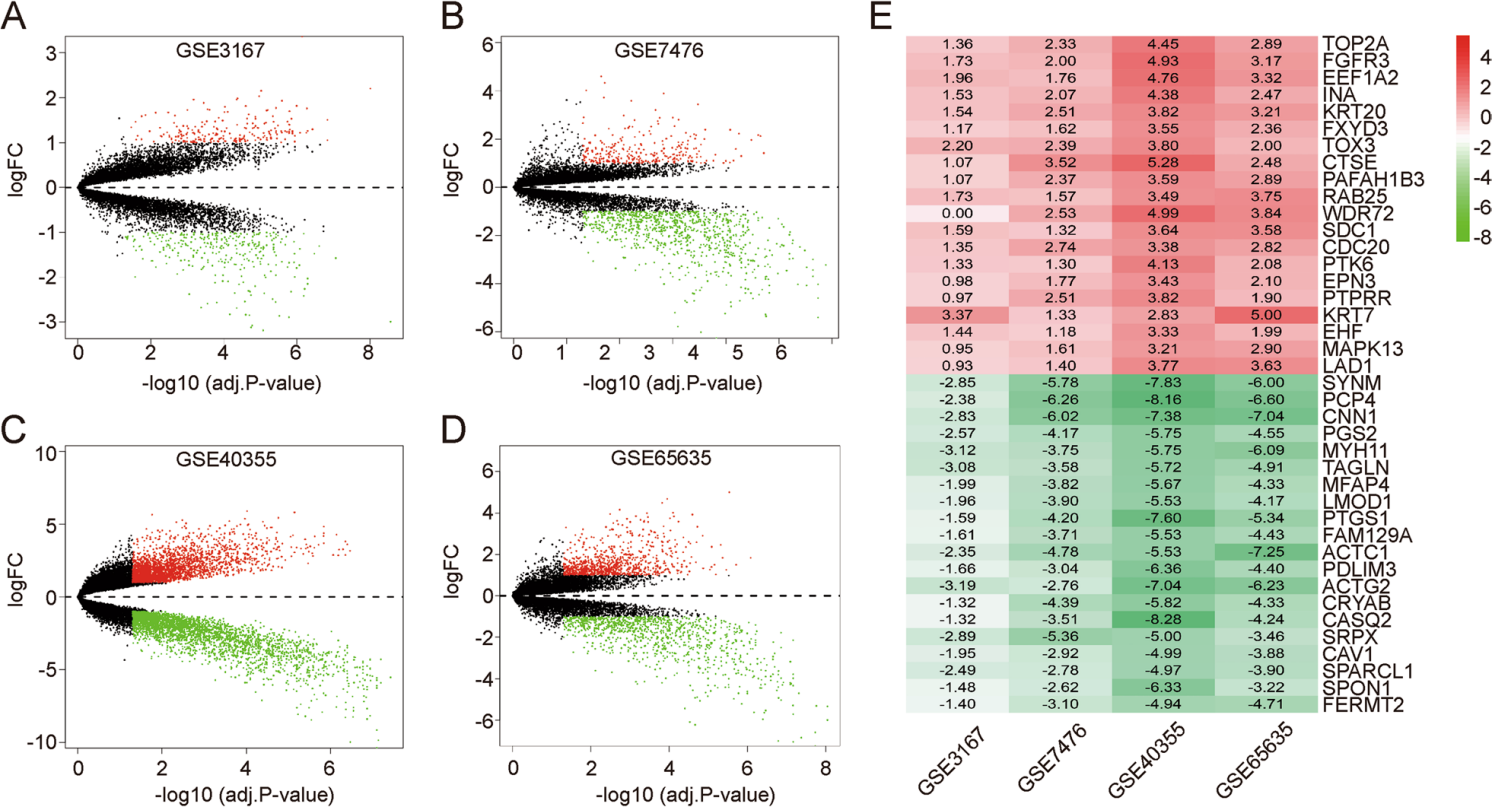
Identification and integration of differentially expressed genes (DEGs). Volcano plots of **A** GSE3167, **B** GSE7476, **C** GSE40355, and **D** GSE65635, and **E** heat map of differentially expressed genes. The abscissa represents the GEO IDs, the ordinate represents the gene name. Red represents logFC > 0; Green represents logFC < 0; and the value in the box represents the logFC

Thirty-nine NMIBC (Ta and T1 stage) and twenty-nine normal BC samples were enrolled in this study (Table 1). The corresponding clinical and pathological information for each dataset is shown in Additional file 2: Table S1. After normalizing the expression of genes in the four microarray datasets (Additional file 1: Fig. S1), 218, 298, 2925, and 855 up-regulated DEGs and 315, 872, 2399, and 1035 down-regulated DEGs were identified in the GSE3176, GSE7476, GSE40355, and GSE65635 datasets, respectively (Fig. 1A–D). Through integrated analysis by the RRA method, a total of 213 DEGs, 62 up-regulated and 151 down-regulated genes, were finally identified (Additional file 2: Table S2). The top 20 up-regulated and down-regulated genes ranked by fold change are displayed in a heat map (Fig. 1E).

**Table 1:** The characteristics of each enrolled bladder cancer datasets downloaded from GEO database

Reference	PMID	Record	BC stage	Platform	Tumor	Normal
Dyrskjot L et al., [11]	15,173,019	GSE3167	Ta/T1 stage	GPL96—[HG-U133A] Affymetrix Human Genome U133A Array	15	14
Mengual L et al., [12]	19,539,325	GSE7476	Ta/T1 stage	GPL570—[HG-U133_Plus_2] Affymetrix Human Genome U133 Plus 2.0 Array	6	3
Hecker N et al., [13]	23,717,626	GSE40355	Ta/T1 stage	GPL13497-Agilent-026652 Whole Human Genome Microarray 4 × 44 K v2	13	8
Borisov N et al., [14]	29,251,172	GSE65635	Ta/T1 stage	GPL14951- Illumina HumanHT-12 WG-DASL V4.0 R2 expression beadchip	5	4

*BC* Bladder cancer

### GO and KEGG pathway enrichment analysis for DEGs

**Fig. 2:**
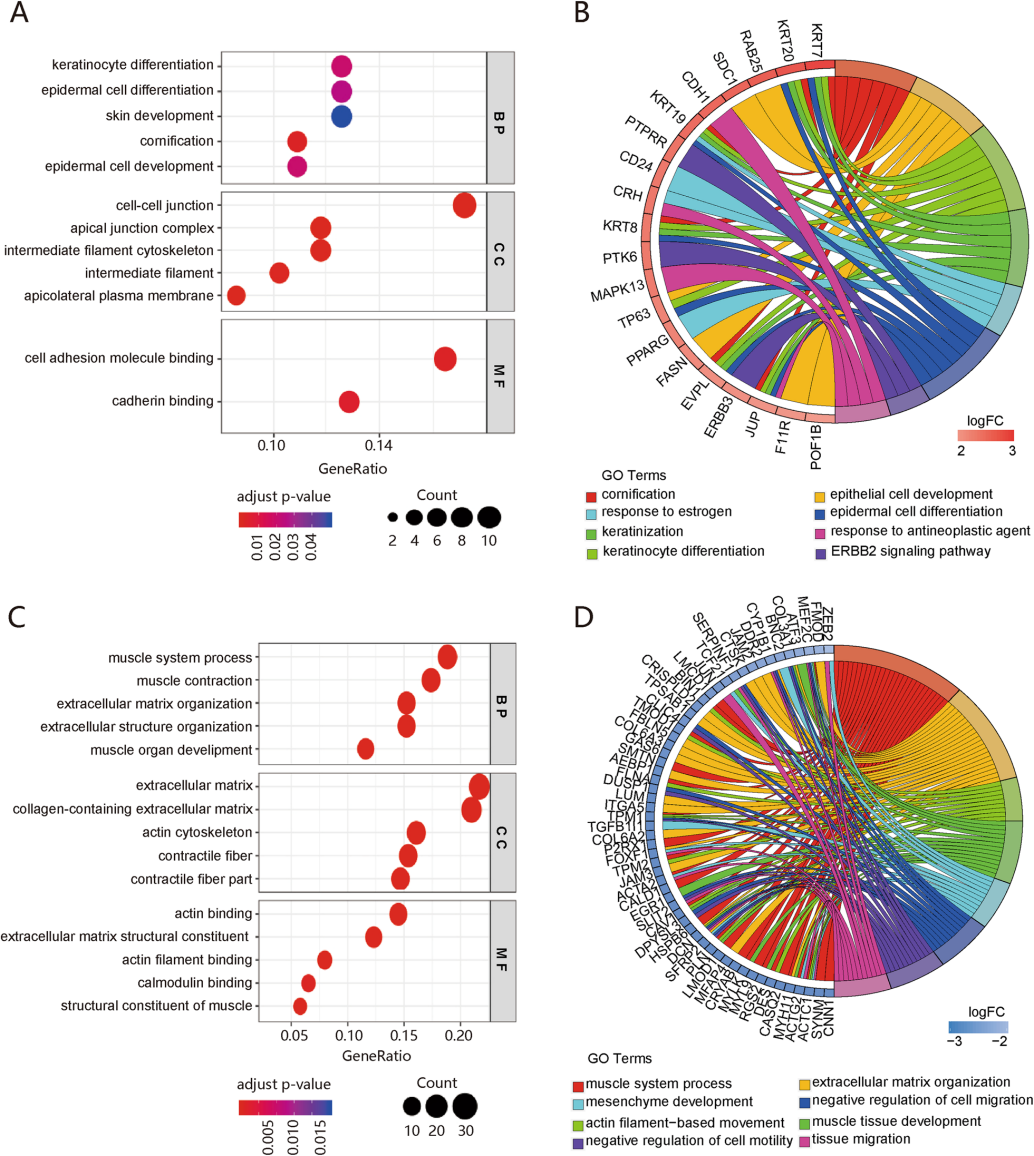
GO terms analysis for DEGs. **A** Bubble plot of prior significantly enriched GO terms of up-regulated DEGs in each classification. **B** GO Chord plot of the relationship between the top eight GO-BP terms and their corresponding up-regulated genes. **A** gene is linked to a certain GO term by colored bands. **C** Bubble plot of the prior significantly enriched GO terms of down-regulated DEGs. **D** GO Chord plot of the relationship between the eight tumor-related enriched GO-BP terms and their corresponding down-regulated genes

**Fig. 3:**
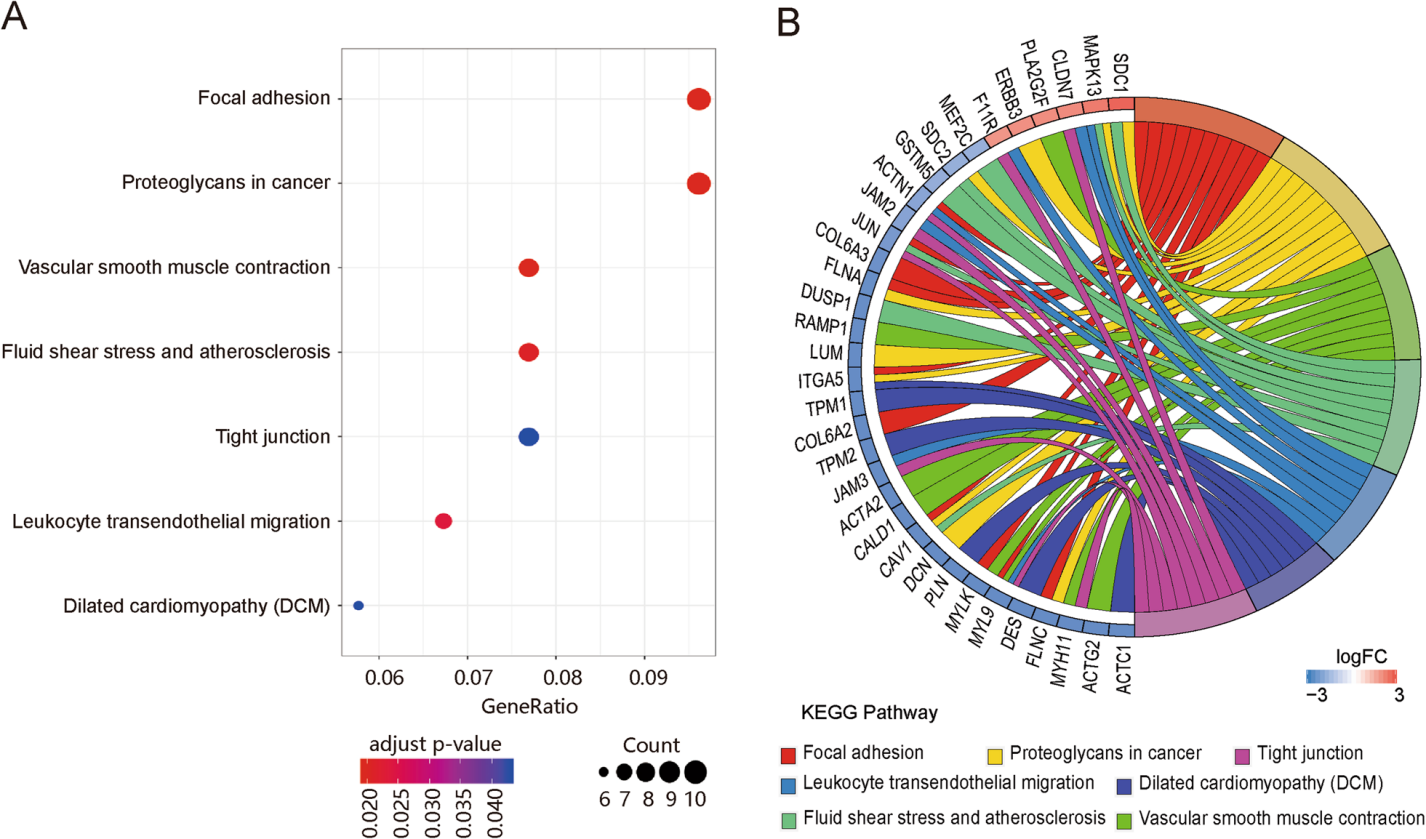
KEGG pathway enrichment analysis for DEGs. **A** Bubble plot of dysregulated pathways. **B** KEGG Chord plot of the relationship between the selected pathways and their corresponding genes

A total of 32 and 173 significantly enriched GO terms (adj. P-value ≤ 0.05) for the up-regulated and down-regulated DEGs, respectively, were identified (Additional file 2: Table S3). The up-regulated genes were mainly enriched in cornification, epithelial cell development, keratinocyte and epidermal cell differentiation, and the ERBB2 signaling pathway (Fig. 2A–B). The down-regulated DEGs were mainly enriched in biological processes related to muscle system and mesenchyme development, extracellular matrix and structure organization, and regulation of cell migration and growth (Fig. 2C–D). KEGG analysis indicated that the integrated DEGs were significantly enriched in seven pathways, including focal adhesion (FA), proteoglycans in cancer, vascular smooth muscle contraction, fluid shear stress and atherosclerosis, leukocyte transendothelial migration, dilated cardiomyopathy (DCM), and tight junctions (Table 2 and Fig. 3A–B).

**Table 2:** KEGG pathway enrichment results for integrated DEGs

Pathway ID	Pathway Description	Count	adj. *P*-value	*q*-value	Gene ID
hsa04510	Focal adhesion	10	1.96E-02	1.79E-02	*CAV1 COL6A2 MYL9 MYLK ITGA5 FLNC COL6A3 JUN ACTN1 FLNA*
hsa05205	Proteoglycans in cancer	10	1.96E-02	1.79E-02	*CAV1 LUM DCN ITGA5 FLNC SDC2 FLNA SDC1 MAPK13 ERBB3*
hsa04270	Vascular smooth muscle contraction	8	1.96E-02	1.79E-02	*MYH11 ACTG2 MYL9 MYLK RAMP1 CALD1 ACTA2 PLA2G2F*
hsa05418	Fluid shear stress and atherosclerosis	8	2.08E-02	1.90E-02	*CAV1 DUSP1 JUN MEF2C GSTM5 SDC2 SDC1 MAPK13*
hsa04670	Leukocyte transendothelial migration	7	2.36E-02	2.16E-02	*MYL9 JAM3 JAM2 ACTN1 MAPK13 CLDN7 F11R*
hsa05414	Dilated cardiomyopathy (DCM)	6	4.29E-02	3.92E-02	*ACTC1 DES ITGA5 TPM1 PLN TPM2*
hsa04530	Tight junction	8	4.29E-02	3.92E-02	*MYH11 MYL9 JAM3 JUN JAM2 ACTN1 CLDN7 F11R*

### Multivariate cox analysis and ROC curve plotting for DEGs

**Fig. 4:**
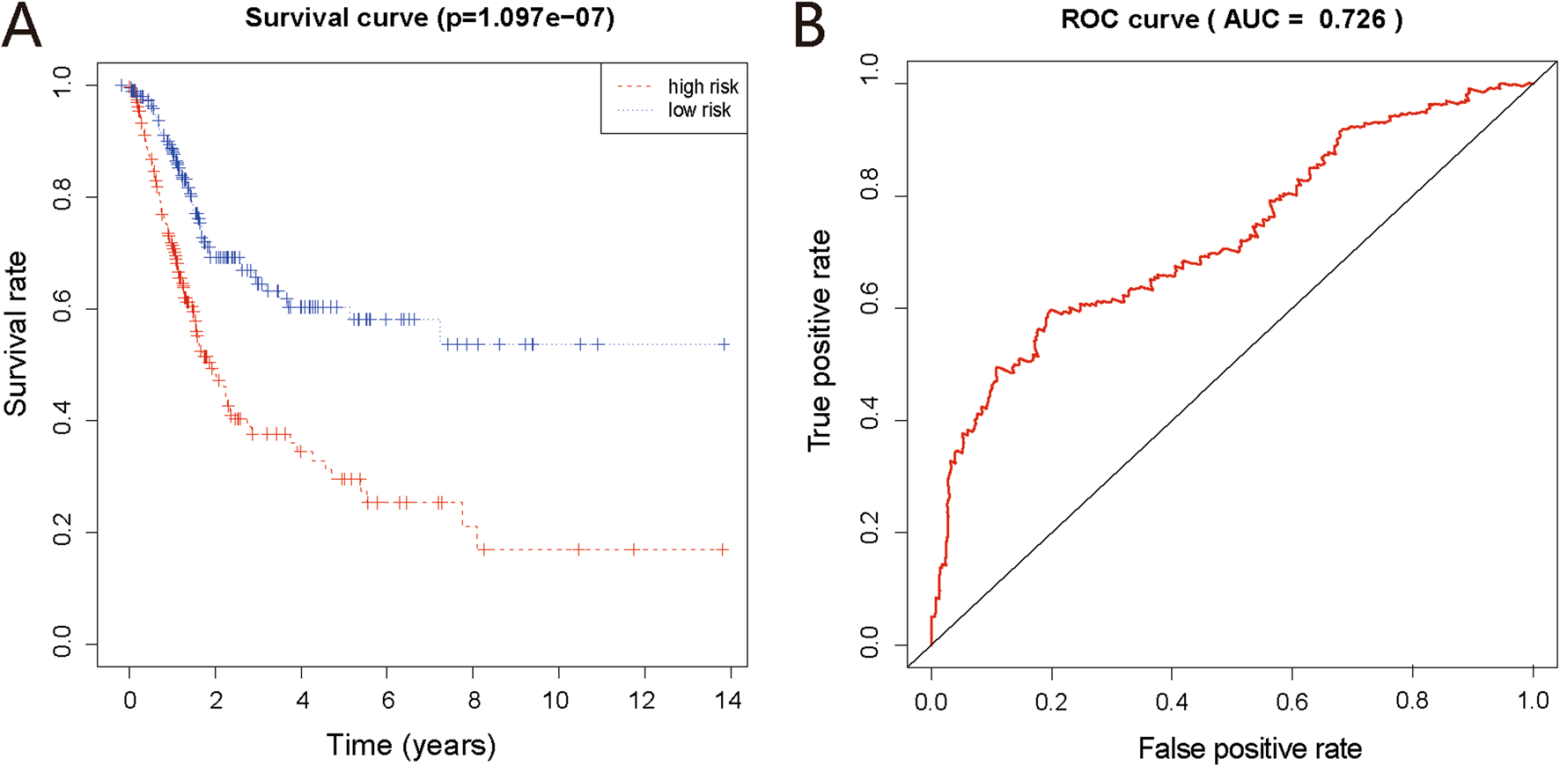
Survival and ROC curve analysis for DEGs. **A** Survival curve. Patients were divided into a high-risk group (> 1.009) and a low-risk group (< 1.009) according to the risk score. The difference in the survival rate between the high-risk and low-risk groups was significant (*P* < 0.001). **B** ROC curve. The calculated AUC was 0.726. The larger the AUC value, the more likely the current classification algorithm placed the positive sample in front of the negative sample

According to the AIC, the optimal risk model containing nine genes was determined based on bladder cancer patient gene expression data provided by the TCGA consortium (Table 3). Of these genes, *FASN, SPAG4, FCRLB,* and *UPK2* showed positive coefficients, indicating their role as risk factors for predicting a poor survival. However, *UBE2C, FER1L4, EPN3, CTSE*, and *TOX3* showed negative coefficients, suggesting that they might be protective factors associated with a longer survival. The calculated median value for the risk score of each sample was 1.009. Each sample was grouped into “high-risk” and “low-risk” according to the median risk score. Survival analysis (Fig. 4A) revealed a five-year survival rate of 27.5% [95% confidence interval (CI), 19.3–39.0%] in the high-risk group (202 patients) and 58.1% (95% CI, 49.1–68.7%) in the low-risk group (203 patients). We plotted the ROC curve for the risk model, which yielded an AUC value of 0.726 (Fig. 4B).

**Table 3:** Coefficients for the nine genes in the optimal risk model for survival of bladder cancer patients

Gene symbol	Description	Coefficient	*P*-value
*UBE2C*	Ubiquitin Conjugating Enzyme E2 C	− 0.23099	8.88E-03
*FASN*	Fatty Acid Synthase	0.31633	5.71E-04
*SPAG4*	Sperm Associated Antigen 4	0.16882	1.49E-01
*FCRLB*	Fc Receptor Like B	0.10812	1.02E-01
*FER1L4*	Fer-1 Like Family Member 4	− 0.35367	7.57E-05
*EPN3*	Epsin 3	− 0.13823	1.18E-01
*UPK2*	Uroplakin 2	0.11497	2.53E-03
*CTSE*	Cathepsin E	− 0.09189	6.96E-02
*TOX3*	TOX High Mobility Group Box Family Member 3	− 0.10033	1.39E-01

Risk score = UBE2C × (− 0.231) + FASN × (0.316) + SPAG4 × (0.169) + FCRLB × (0.108) + FER1L4 × (-0.354) + EPN3 × (− 0.138) + UPK2 × (0.115) + CTSE × (− 0.092) + TOX3 × (− 0.100)

Likelihood ratio test = 82.23 on 9 df, *p* = 5.83e-14, *n* = 530

### Integration of PPI network and module analysis

**Fig. 5:**
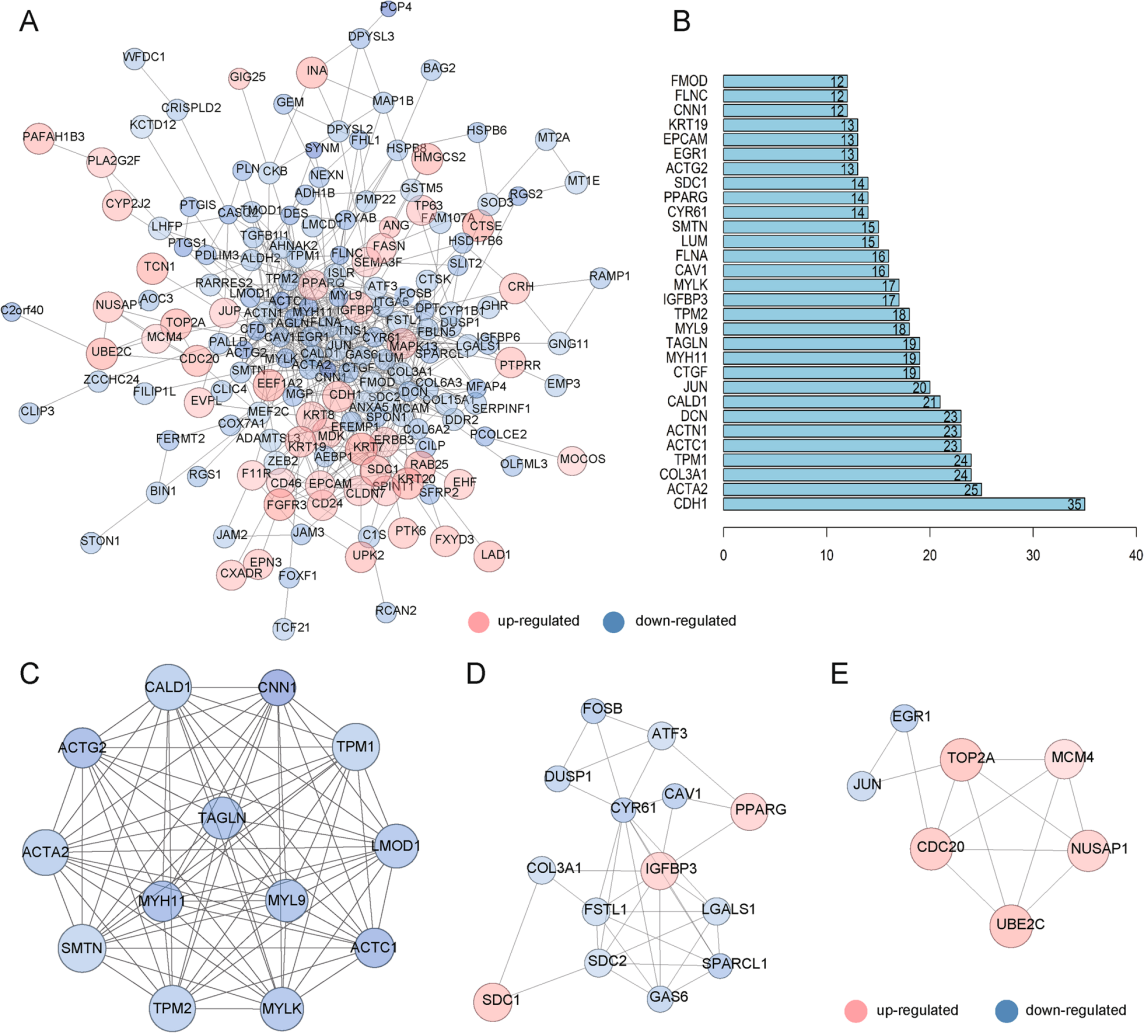
PPI network construction and module analysis for DEGs. **A** The PPI network of DEGs was constructed, and DEGs with ≤ 3 nodes and ≤ 3 edges were removed from the network. Up-regulated genes are marked in red, and down-regulated genes are marked in green. Larger sizes or darker colors of a node indicate that the corresponding gene had a greater logFC. **B** Bar plot of the nodes with top 30 degrees in the PPI network. Higher numbers and longer bar lengths represent greater interactions of the protein. **C** The most significant module in the PPI network had 13 nodes and 77 edges. **D** The second most significant module in the PPI network had 14 nodes and 36 edges. **E** The third most significant module in the PPI network had 7 nodes and 13 edges

A PPI network of the integrated DEGs was constructed that comprised 171 nodes and 561 edges (Fig. 5A). Then, the top 30 DEGs ranked by degree value in the PPI network were identified (Fig. 5B). After the module analysis of the PPI network, the top three significant modules were determined (Fig. 5C–E) and enriched in several KEGG pathways. Thirteen genes in module 1 were significantly enriched in vascular smooth muscle and cardiac contraction and pathways associated with cardiomyocyte structural and functional abnormalities. Fourteen genes in module 2 were significantly enriched in proteoglycans in cancer, malaria, and ECM–receptor interaction, and seven genes in module 3 were significantly enriched for HTLV-I infection, cell cycle, and ubiquitin-mediated proteolysis (Additional file 2: Table S4).

### Identification and survival analysis for hub genes

**Fig. 6:**
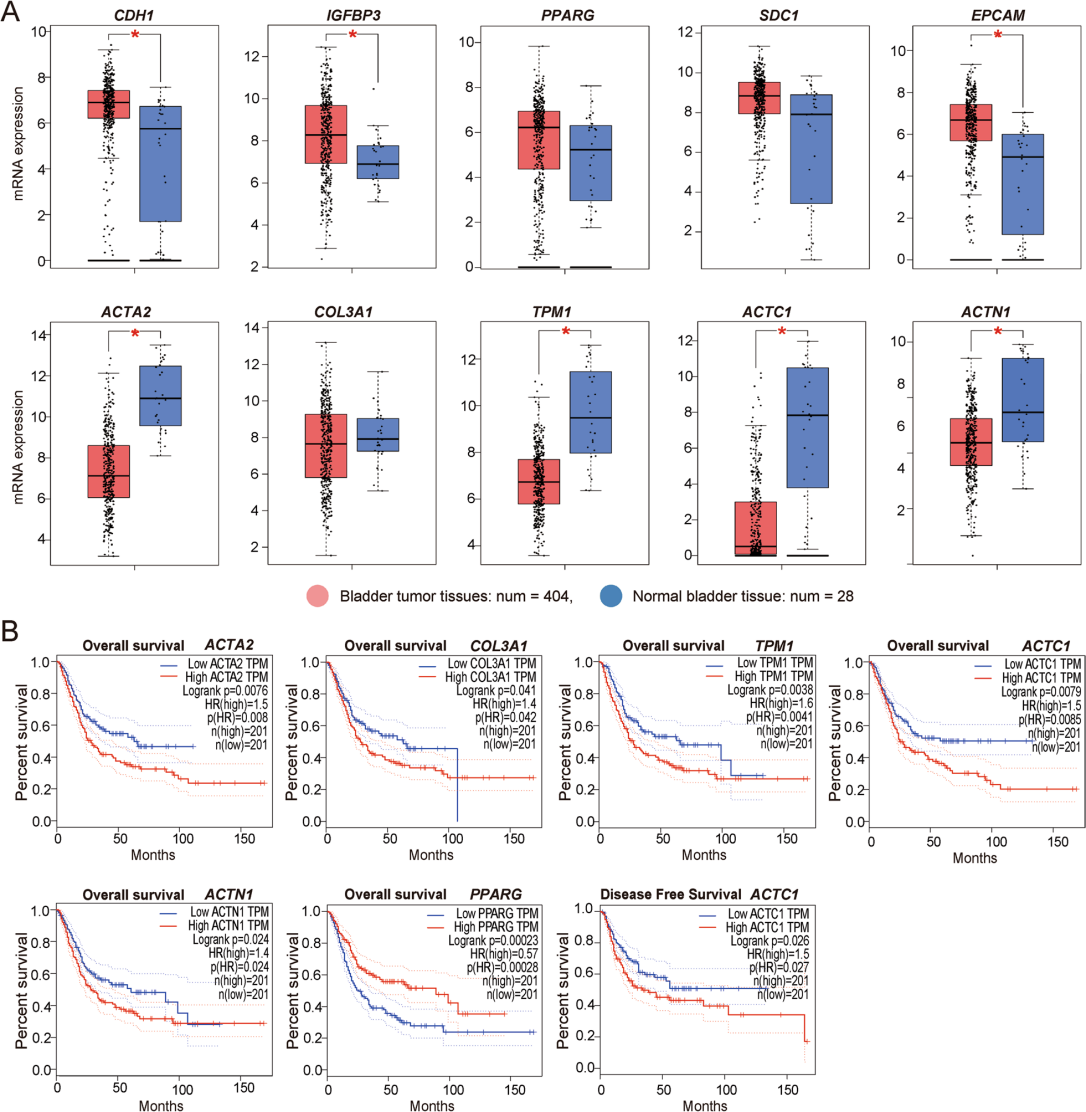
Expression verification and survival analysis for hub genes. **A** mRNA expression levels of the ten hub genes using GEPIA online tool. *P < 0.01. **B** Overall survival (OS) and disease free survival (DFS) analyses and for the hub genes using the GEPIA online tool. Only the genes whose altered expression significantly affect OS and DFS are shown

According to the degree value in the PPI network, up-regulated DEGs exhibiting the top five high degree genes (*CDH1*, *IGFBP3*, *PPARG*, *SDC1,* and *EPCAM*) and down-regulated DEGs exhibiting the top five high degree genes (*ACTA2*, *COL3A1*, *TPM1*, *ACTC1*, and *ACTN1*) were screened out as hub genes. The gene descriptions, fold changes, and corresponding degree values for the hub gene are presented in Table 4. GO analysis for the hub genes identified eleven significantly enriched GO terms, which were the most associated with the differentiation and development of muscle cells and the cytoskeleton (adj. *P*-value ≤ 0.05) (Additional file 2: Table S5). The hub genes were also confirmed to be expressed in many BC tissue samples (Fig. 6A), showing that *CDH1*, *IGFBP3*, and *EPCAM* were significantly up-regulated in BC samples from different stages, while *ACTA2*, *TPM1*, *ACTC1,* and *ACTN1* were significantly down-regulated. In addition, an analysis of the correlation between the OS and DFS of patients with BC and hub genes showed that patients with BC showing altered expression of *ACTA2, COL3A1, TPM1, ACTC1*, and *ACTN1* exhibited a worse OS, while those with altered expression of *PPARG* had a better OS, and only high-expressed *ACTC1* could predict a worse DFS (Fig. 6B).

**Table 4:** The fold change and degree values for the hub genes

Gene symbol	Description	LogFC	Degree
*CDH1*	Cadherin 1	2.49	35
*ACTA2*	Actin Alpha 2	− 3.39	25
*COL3A1*	Collagen Type III Alpha 1 Chain	− 2.24	24
*TPM1*	Tropomyosin 1	− 3.08	24
*ACTC1*	Actin Alpha Cardiac Muscle 1	− 4.98	23
*ACTN1*	Actinin Alpha 1	− 2.31	23
*IGFBP3*	Insulin Like Growth Factor Binding Protein 3n	2.08	17
*PPARG*	Peroxisome Proliferator Activated Receptor Gamma	2.02	14
*SDC1*	Syndecan 1	2.53	14
*EPCAM*	Epithelial Cell Adhesion Molecule	1.91	13

*F*C Fold change

### Correlation between CDH1 expression and clinicopathological characteristics in BC

**Fig. 7:**
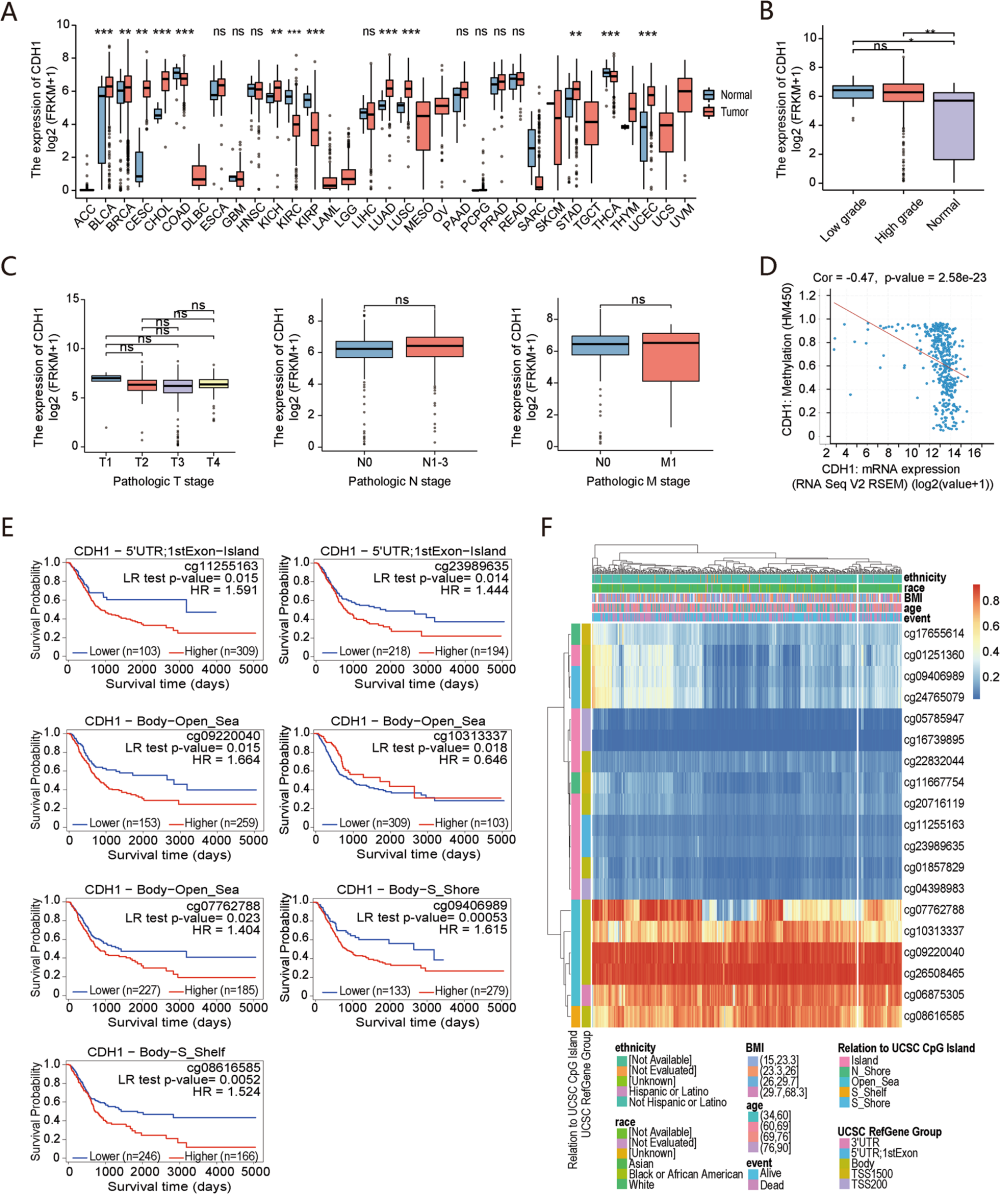
Association of *CDH1* expression with clinicopathological characteristics and *CDH1* methylation with prognosis in BC. **A** Comparison of *CDH1* expression between tumor and para-carcinoma tissues in different types of cancers based on TCGA database. ns, *P* > 0.05; *P < 0.05; **P < 0.01; ***P < 0.001. **B** *CDH1* expression in different histological grade BC tissues and normal bladder tissues. **C** Patients with different T, N, and M stages shared similar *CDH1* expression levels in BC. **D** Correlation between *CDH1* methylation and its expression level in BC. **E** *CDH1* methylation was correlated with prognosis in BC from the MethSurv database. **F** Heat maps of the association between the methylation level of *CDH1* and patient characteristics and genomic subregions

As the gene with the highest degree in the PPI network, *CDH1* was significantly overexpressed in multiple tumor tissues, especially in tumors originating from urogenital tracts, breast, lung, cholangio, cervix, and endometrium, based on TCGA database. However, in colon adenocarcinoma, renal clear cell and papillary cell carcinoma, and thyroid carcinoma, *CDH1* was significantly decreased (Fig. 7A). Moreover, *CDH1* showed a significantly higher expression in patients with low and high histological grades than in normal BC tissues (Fig. 7B). However, patients with different histological grades, i.e., T, N, and M stages, shared similar *CDH1* expression levels (Fig. 7C). Given the results of *CDH1* expression in patients with Ta/T1 stage BC, we speculate that *CDH1* is markedly up-regulated in the tumor initiation stage but not further altered during the tumor development phase.

### Correlation between methylation of CDH1 and prognosis in BC

Although altered expression of *CDH1* did not significantly influence the OS rate in patients with BC, DNA methylation also affects clinical outcomes. The cBioPortal platform was used to evaluate *CDH1* methylation in BC, showing that *CDH1* expression was highly negatively correlated with methylation (R =  − 0.47, *P* < 0.001) in BC (Fig. 7D). Kaplan–Meier plots by MethSurv analysis were drawn to identify methylation sites in *CDH1* associated with prognosis in BC. We found six methylated sites indicating good prognosis (5′-UTR;1stExon-Island-cg11255163 and cg23989635, Body-Open_Sea-cg09220040 and cg07762788, Body-S_Shore-cg09406989, and Body-S_Shelf-cg08616585) and one site indicating an aberrant prognosis (Body-Open_Sea-cg10313337) (Fig. 7E). The heat map plotted by ‘Gene Visualization’ further illustrates the relationship of *CDH1* methylation levels with gene subregions and available characteristics of patients (Fig. 7F).

### CDH1 expression and identification of correlated genes in BC cell lines

**Fig. 8:**
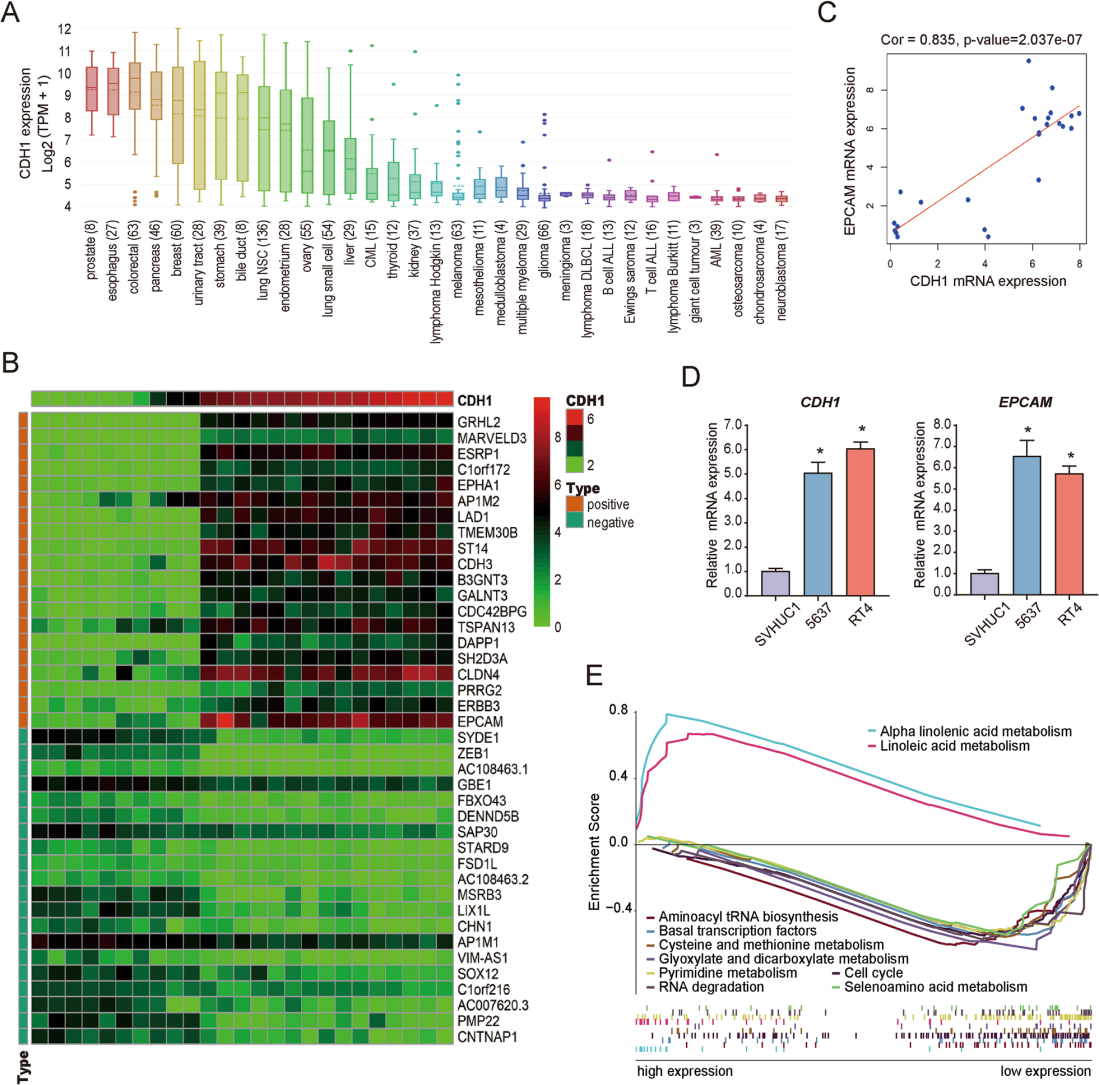
*CDH1* expression in vitro and corresponding gene identification and function analysis. **A** *CDH1* expression in various cancer cell lines based on the CCLE database. The ordinate represents the expression level of *CDH1*. **B** Heat map of the top 20 positively and negatively co-expressed genes with *CDH1* in urinary tract cancer cell lines extracted from the CCLE database. **C** Correlation between the expression of *CDH1* and *EPCAM*. Cor is the correlation coefficient. **D** mRNA expression levels of *CDH1* and *EPCAM* in SVHUC1 cells, and 5637 and RT4 bladder tumor cells examined using RT-PCR. Three independent experiments were conducted for each RT-PCR assay, and *P* < 0.05 was considered to reflect a statistically significant difference from the SVHUC1 group. **E** GSEA function enrichment analysis of differentially expressed genes in the high *CDH1* expression group and low *CDH1* expression group based on the CCLE database

Based on the CCLE database, *CDH1* was also significantly overexpressed in several different cancer cells (Fig. 8A), derived from the urinary tract, breast, lung, cholangio, prostate, esophagus, colorectal, and endometrium, which generally conformed to that in tumor tissues. Through co-expression analysis in 25 different urinary tract cancer cell lines extracted from the CCLE database, a total of 755 and 197 genes were identified that were positively and negatively co-expressed with *CDH1*, respectively (Additional file 2: Table S6). The top 20 positively and negatively co-expressed genes are depicted in a heat map (Fig. 8B). Notably, another hub gene, *EPCAM*, had a significant positive correlation of 0.805 with *CDH1* (Fig. 8C). Both *CDH1* and *EPCAM* were confirmed by qRT-PCR to be up-regulated in 5637 and RT4 BC cells compared to those in the normal urinary tract epithelial cell line SVHUC1 (Fig. 8D). High and low expression *CDH1* phenotypes of significantly enriched pathways, including representative metabolic signaling, cell cycle, and RNA degradation pathways (Fig. 8E and Additional file 2: Table S7), were determined using GSEA.

### Correlation between CDH1 expression and immune cell infiltration in BC

**Fig. 9:**
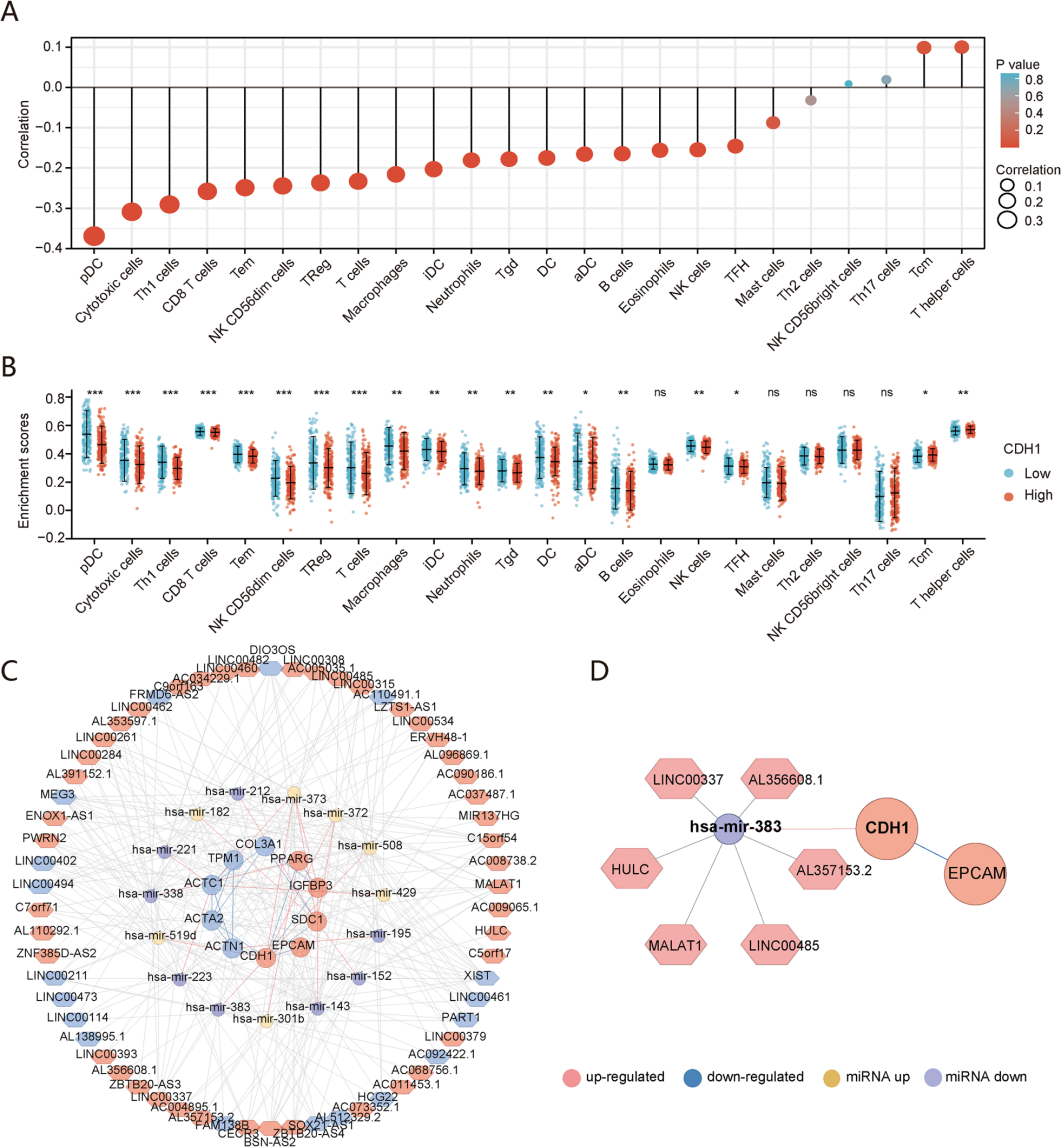
Correlation between *CDH1* expression and immune cell infiltration in BC and the ceRNA network construction. **A** Relationships among infiltration levels of 24 immune cell types and *CDH1* expression profiles in BC samples based on the TCGA database by Spearman correlation analysis. **B** The infiltration levels of the 24 immune cell types were evaluated in distinct *CDH1* groups in BC samples. ns, *P* > 0.05; *P < 0.05; **P < 0.01; ***P < 0.001. DCs, dendritic cells; aDCs, activated DCs; iDCs, immature DCs; pDCs, plasmacytoid DCs; NK, natural killer cells; Th, T helper cells; Th1, type 1 Th cells; Th2, type 2 Th cells; Th17, type 17 Th cells; Tcm, T central memory cells; Tem, T effector memory cells; Tfh, T follicular helper cells; Tgd, T gamma delta cells; Treg, regulatory T cells. **C** ceRNA network containing hub genes, differently expressed miRNAs and lncRNAs in BC. **D** ceRNA network associated with *CDH1*. Larger circular nodes represent mRNA, smaller circular nodes represent miRNA, and hexagonal nodes represent lncRNA

By the ssGSEA method, we quantified the infiltration levels of 24 immune cell types for 413 BC samples of TCGA-BLCA and investigated the association between *CDH1* expression and immune cell infiltration. Spearman correlation analyses revealed that high *CDH1* expression was mainly associated with low infiltration of the most immune cell types (Fig. 9A), especially pDCs (*R* = − 0.369, *P* < 0.001), cytotoxic cells (*R* = − 0.309, *P* < 0.001) and Th1 cells (*R* = − 0.291, *P* < 0.001). Besides, CD8 + T cells (*R* = − 0.258, *P* < 0.001), Treg (*R* =− 0.233, *P* < 0.001), T cells (*R* = − 0.233, *P* < 0.001), macrophages (*R* = − 0.216, *P* < 0.001), neutrophils (*R* = -0.181, *P* < 0.001), DCs (*R* = − 0.175, *P* = 0.001), B cells (*R* = − 0.165, *P* < 0.001), and NK cells (*R* = − 0.155, *P* = 0.002) were all negative correlated with *CDH1* expression. We observed weakly positive correlations of *CDH1* expression level with infiltration of only two immune cell types, including T helper cells (*R* = 0.1, *P* = 0.042) and Tcm cells (*R* = 0.099, *P* = 0.045). The immune cells relevant to infiltration levels were further evaluated in distinct *CDH1* groups (Fig. 9B), which conformed to the results in Fig. 9A.

### Construction of ceRNA network

We identified 556,431 significantly up-regulated and 125 significantly down-regulated DElncRNAs and 289,181 significantly up-regulated and 108 significantly down-regulated DEmiRNAs between BC and adjacent non-cancer bladder tissues in TCGA database (Additional file 1: Fig S2). Furthermore, a total of 552 miRNAs related to hub genes were predicted by integrating the results obtained in the TargetScan and miRDB databases (Additional file 2: Table S8). Subsequently, a ceRNA network was constructed using DEmiRNAs that interacted with both hub genes and DElncRNAs (Additional file 2: Table S9), including 46 lncRNA nodes, 42 miRNA nodes, and 10 mRNA nodes (Fig. 9C). Of these, hsa-miR-383 was the only significantly down-regulated miRNA interacting with *CDH1* in BC and associated with LINC00337, AL356608.1, AL357153.2, LINC00485, MALAT1, and HULC, which were all significantly up-regulated in BC (Fig. 9D).

## Discussion

In this study we included four independent datasets of early-stage BC and identified 213 DEGs through integrated analysis. Most GO terms these DEGs enriched were closely associated with the development of BC, such as keratinocyte and epidermal cell differentiation, cell–cell junction, muscle system processes, regulation of cell migration and growth, etc. Besides, DEGs participated in the control of various pathways, including focal adhesion, proteoglycans in cancer, vascular smooth muscle contraction, leukocyte transendothelial migration, and tight junction signaling, contributing to BC progression. FA-related structural molecules are associated with cancer progression and metastasis by promoting cell invasion and epithelial–mesenchymal transition (EMT) [7, 30]. Moreover, emerging evidence supports a critical role of proteoglycans in maintaining homeostasis and carcinogenesis [31]. The contraction of vascular smooth muscle tissue is critical for evaluating vasoactivity [32], and the disruption of tight junctions is a vital step during EMT [33]. Thus, these pathways are potential targets for developing therapeutic strategies for cancer treatment [33, 34]. Leukocyte transendothelial migration has been linked to adhesion molecules and chemokines [35] and is a key step in cancer progression [36]. In this study, an optimal risk model with an AUC value of 0.726 was generated using multivariate Cox analysis of all DEGs, indicating a highly predictive model for patient prognosis of survival in BC.

Normally, the degree of connectivity of a gene in a PPI network reflects its association with corresponding disease, so up-regulated DEGs exhibiting the top five high degree genes (*CDH1*, *IGFBP3*, *PPARG*, *SDC1*, and *EPCAM*) and down-regulated DEGs exhibiting the top five high degree genes (*ACTA2*, *COL3A1*, *TPM1*, *ACTC1,* and *ACTN1*) were screened out as hub genes. These hub genes were predominantly enriched in pathways associated with muscle cell differentiation and development, regulation of cytoskeleton, and growth factor binding. We compared additional BC and adjacent normal tissues on the GEPIA platform to assess whether the expression trends of these hubs were similar when including intermediate and advanced stage BC tissues as compared to when only early-stage tumors were included. We found that the trends in the expression of *CDH1*, *IGFBP3*, *EPCAM, ACTA2*, *TPM1*, *ACTC1,* and *ACTN1* in BC samples containing early-advanced stages were consistent with those in early-stage BC. However, significant changes of *COL3A1*, *PPARG,* and *SDC1* were not detected, implying that their expression appeared to be specifically altered in early-stage BC only. In addition, survival analysis indicated that changes in the expression of *ACTA2*, *COL3A1*, *TPM1*, *ACTC1,* and *ACTN1* were associated with a poor prognosis of BC, while *PPARG* overexpression indicated a better OS outcome.

Some studies have suggested that PPARG-dependent transcriptional regulation may be involved in the etiology of urothelial carcinoma [37], and decreasing PPARG activity via drug inhibition or gene ablation inhibits the proliferation of BC cells [38]. *SDC1* encodes essential cell surface adhesion molecules that maintain cell morphology and a stable microenvironment and promotes tumor progression by stimulating cell proliferation, metastasis, invasion, and angiogenesis [39]. *ACTB2* is closely associated with cell motility, structure, and integrity; abnormal expression of this gene accelerates the invasion and metastasis of lung adenocarcinoma [40], suggesting that *ACTB2* is a potential mass spectrometry-based diagnostic protein marker for BC [41]. Down-regulation of *TPM1* has been detected in both gastric [42] and colorectal cancer [43] during tumor invasion and lymph node metastasis, which are closely associated with BC progression [44], suggesting a role as tumor suppressor. However, increased expression of *COL3A1* in BC predicts a poor prognosis [45], consistent with our findings. *ACTC1* has been linked to cancer recurrence and OS rate in glioma patients, suggesting that it may be a novel independent marker for prognosis and invasion in glioma [46]. *ACTN1* encodes a non-muscular alpha-actin subtype involved in keratinocyte motility by modulating the actin cytoskeleton, focal adhesion, and hemidesmosomal protein complexes, which in turn modulates cell velocity, lipid dynamics, and directional migration [47]. Decreased expression of *ACTN1* may improve survival in pancreatic cancer [48]. Recently, *IGFBP3* was found to be overexpressed in various tumor types and is not only associated with increased incidence in colorectal cancer [49] but also leads to tumor metastasis by increasing cell migration and adhesion in nasopharyngeal carcinoma [50]. In addition, evidence suggests that genetic polymorphisms in *IGFBP3* may be associated with BC tumorigenesis [51]. Brunner et al. [52] found that *EPCAM* is associated with advanced stage, high grade tumor and poor OS rate in patients with BC, suggesting that it is potential novel predictive marker and a therapeutic target for BC.

*CDH1* has the highest degree value in PPI network and was significantly overexpressed in BC tissues, consistent with its overexpression in multiple human cancers. We then found that patients with different histological grades (T, N, and M stages) shared similar *CDH1* expression levels. Taking the results of *CDH1* expression in Ta/T1 stage BC, we speculate that *CDH1* is markedly up-regulated from the initiation tumor stage but not further altered during the tumor development phase, making the value of CDH1 in prediction of BC more prominent than other hub genes. This suggests that high *CDH1* expression could predict the presence of early-advanced stage BC, where its expression rises to the peak in the early stage. Notably, although there was no significant correlation between increased *CDH1* expression and survival times in our study, loss of CDH1 function contributes to cancer progression by increasing proliferation, invasion, and metastasis in various tumor, such as gastric, breast, and colorectal cancers [53]. Liu Jia et al. reported that in various tumor cells, CDH1 can inhibit PI3K/Akt oncogenic signaling to suppress tumorigenesis [54]. All these findings implied that CDH1 would be a potential therapeutic target for cancers. *CDH1* expression was highly negatively correlated with methylation in BC. Methylation of CDH1 is more frequent in BC tissues than in normal control tissues and increasing scientific evidences has suggested that CDH1 gene promoter polymorphism and DNA methylation might contribute to the development and progression of BC [55]. By survival analysis using the MethSurv web tool, patients with high *CDH1* methylation generally had a poorer OS rate than patients with low *CDH1* methylation. Additionally, several methylated sites in *CDH1* associated with prognosis have also been identified, representing abnormal demethylated sites of *CDH1* in BC. *CDH1* methylation might be a promising prognostic biomarker for BC.

We also investigated the underlying relationship between *CDH1* expression and immune cell infiltration in BC. A high *CDH1* expression was negatively correlated with immune cell infiltration, such as pDCs, Treg, T cells, macrophages, neutrophils, DCs, and NK cells. Among them, pDCs were the most significantly negatively associated immune cells with a high *CDH1* expression. The role of pDCs in different tumor progression stages remains controversial. pDCs can promote tumor cell growth, survival, and drug resistance in multiple myeloma cells and xenograft models [56]. pDC is a unique DC subset, which is considered to play a significant role in immune responses. Activated pDCs can secrete large quantities of type I interferon and are involved in the activation and function regulation of NK cells, B cells, and T cells [57]. pDCs can also induce Treg cell generation, and pDC depletion leads to decreased Treg numbers in the tumor microenvironment [58]. Treg cells have been traditionally regarded as cancer promoters, owing to their function in suppressing antitumor immune responses [59]. In contrast, macrophage and neutrophil infiltration were also negatively correlated with a high *CDH1* expression. Different subtypes of macrophages have different effects in tumor development, among which the M2 subtype can promote tumor progression [60]. Tumor neutrophil infiltration also contributes to tumor growth [61]. Therefore, increased *CDH1* expression appears to improve tumor immunity by inhibiting pDCs and macrophage and neutrophil accumulation and by reducing Treg generation via the suppression of pDCs to restrict the escape of cancer cells from annihilation and, ultimately, relieve tumorigenesis.

To further explore the mechanism of CDH1 underlying BC tumorigenesis, we investigated the correlated genes of *CDH1* in urothelial carcinoma cells and their potentially enriched functions. As a result, another hub gene, *EPCAM*, exhibited a highly positive correlation with *CDH1*. We confirmed that both *CDH1* and *EPCAM* expression was up-regulated in BC cells. EPCAM is an epithelial cell adhesion molecule localized on the cell surface and mainly overexpressed in various epithelial malignancies [62]. Increasing evidence suggests that EPCAM is one of the most highly immunogenic tumor-associated antigens. *EPCAM* promotes proliferation, metastasis, and invasion of tumor cells, but overexpressed *EPCAM* is associated with a better prognosis in patients with adenocarcinoma of the lung, breast and gall bladder cancer, and squamous cell carcinoma of the esophagus [62]. The actual contribution of *EPCAM* to tumorigenesis and its prognostic potential for various cancers remain to be explored, which may be mediated via interaction with self-related signaling and other proteins in the plasma membrane, regulation in cancer stem cells, or DNA methylation.

After analyzing the potential function associated with *CDH1*-related genes, we found that a decreased *CDH1* expression corresponded to a significant enrichment of its co-expression partners in many metabolic pathways, cell cycle, and RNA degradation. The propensity of cancer cells to reprogram their metabolism [63] and the disruption of the cell cycle balance [64] have been recognized as key steps in promoting carcinogenesis. Therefore, targeting these metabolic and cell cycle pathways has been the focus of cancer therapy research. As a key factor involved in cell cycle regulation, *CDH1* is essential for cell viability and cell cycle progression and regulates the cell cycle by regulating the Claspin/Chk1 and Rb/E2F1 pathways [65]. Furthermore, the activation of the CDH1–APC axis serves an important function during G1 phase arrest and DNA damage-induced G2 phase arrest [66]. *EPCAM* has also been described as promoter of cell cycle progression, which up-regulates the proto-oncogenes, C-MYC and cyclin A/E [67]. Moreover, the ceRNA analysis revealed that *CDH1* may be regulated by miR-383 in BC. miR-383 is a tumor suppressor that inhibits cell proliferation, metastasis, and EMT in BC via targeting ETS1 [68], which can also suppress cell cycle progression in gastric carcinoma cells through regulating Cyclin E2 expression [69]. However, to date, studies on the association between miR-338 and *CDH1* are lacking. Therefore, further research is required to verify the correlation between *CDH1* and miR-338 underlying BC carcinogenesis and progression.

There were several limitations in this study. First, the sample size was relatively small, and the genetic data lacked ethnic and geographical diversity, potentially influencing our analysis of gene expression in early-stage BC. Second, as gender, age, and pathological typing were not accounted for in this study, it is likely that some biological information is missing.

## Conclusions

In summary, *CDH1*, *IGFBP3*, *PPARG*, *SDC1*, *EPCAM*, *ACTA2*, *COL3A1*, *TPM1*, *ACTC1*, and *ACTN1*, were identified as vital players in the progression of early-stage BC. *CDH1* appeared to be significantly up-regulated from the tumor initiation stage, without further alterations during the tumor development phase. Six methylated sites in *CDH1* were identified indicating a good prognosis in BC patients and one site for an aberrant outcome. Besides, a high *CDH1* expression was negatively correlated with immune cell infiltration, such as pDCs, Treg, T cells, macrophages, neutrophils, DCs, and NK cells. Moreover, *EPCAM* was highly positively correlated with *CDH1,* which was predicted to be directly regulated by miR-383 in BC. These biomarkers could serve as potential predictors and therapeutic targets for early-stage BC, which are required to be future confirmed experimentally.

## Supplementary Information

**Additional file 1: Figure S1. **Normalisation of gene expression. **Figure S2.** DElncRNAs and DEmiRNAs in patients with BC.

**Additional file 2: Table S1. **The clinical information of the samples in each Dataset. **Table S2.** Information of the integrated DEGs. **Table S3.** GO terms enrichment analysis of EGs. **Table S4.** KEGG enrichment for the top significant modules of the PPI network. **Table S5.** GO terms analysis for the hub genes. **Table S6.** Genes correlated with CDH1 based on CCLE database. **Table S7.** The GSEA results for CDH1 in BC cell lines. **Table S8.** The miRNAs related to hub genes. **Table S9.** The miRNAs paired with DElncRNAs and hub genes.

## Data Availability

The datasets generated and/or analysed during the current study are available in the GEO repository, which can be found here: https://www.ncbi.nlm.nih.gov/geo/query/acc.cgi?acc=GSE3167 (accessed on 10 August 2018); https://www.ncbi.nlm.nih.gov/geo/query/acc.cgi?acc=GSE7476 (accessed on 25 March 2019); https://www.ncbi.nlm.nih.gov/geo/query/acc.cgi?acc=GSE40355 (accessed on 01 February 2019); https://www.ncbi.nlm.nih.gov/geo/query/acc.cgi?acc=GSE65635 (accessed on 13 August 2019).

## Abbreviations

BP: Biological processes
CC: Cell component
CCLE: Cancer cell line encyclopedia
ceRNA: Competitive endogenous RNA
CI: Confidence interval
DEGs: Differentially expressed genes
DElncRNAs: Differentially expressed lncRNAs
DEmRNAs: Differentially expressed microRNAs
DFS: Disease free survival
FC: Fold change
FDR: False-discovery rate
GEO: Gene expression omnibus
GEPIA: Gene expression profiling interactive analysis
GO: Gene ontology
GSEA: Gene set enrichment analysis
KEGG: Kyoto encyclopedia of genes and genomes
MCODE: Molecular complex detection
MF: Molecular function
NES: Normalised enrichment score
OS: Overall survival
PPI: Protein–protein interaction RCC
ROC: Receiver operating-characteristic
STRING: Search tool for the retrieval of interacting genes
TCGA: The cancer genome atlas
