# Supplementary material for: CDH1 overexpression predicts bladder cancer from early stage and inversely correlates with immune infiltration
Source: BMC Urol. 2022 Sep 21;22:156. doi: 10.1186/s12894-022-01103-7 (PMC9494810; doi:10.1186/s12894-022-01103-7)
Supplement: Supplementary file 1 — Additional file 1: Figure S1. Normalisation of gene expression. Figure S2. DElncRNAs and DEmiRNAs in patients with BC. [file 12894_2022_1103_MOESM1_ESM.pdf]

BMC Urology

CDH1 Overexpression Predicts Bladder Cancer from Early Stage and Inversely Correlates with Immune Infiltration

Tao Fan[1,2#], Liang Xue[2#], Houguang He[1,2#], Wenda Zhang[1,2], Lin Hao[1,2,3],Weiming Ma[1,3], Bingzheng Dong[1,3], Guanghui Zang[1], Conghui Han[1,2,3], Yang Dong[1,3]*

***Correspondence:**

Yang Dong: E-mail: ydong0802@stu.suda.edu.cn

Department of Urology, Xuzhou Central Hospital,

Jiefang South Road, No. 199, Xuzhou, Jiangsu, China

Additional file

Additional file 1:

Supplementary Figure S1. Normalisation of gene expression.

Supplementary Figure S2. DElncRNAs and DEmiRNAs in patients with BC.

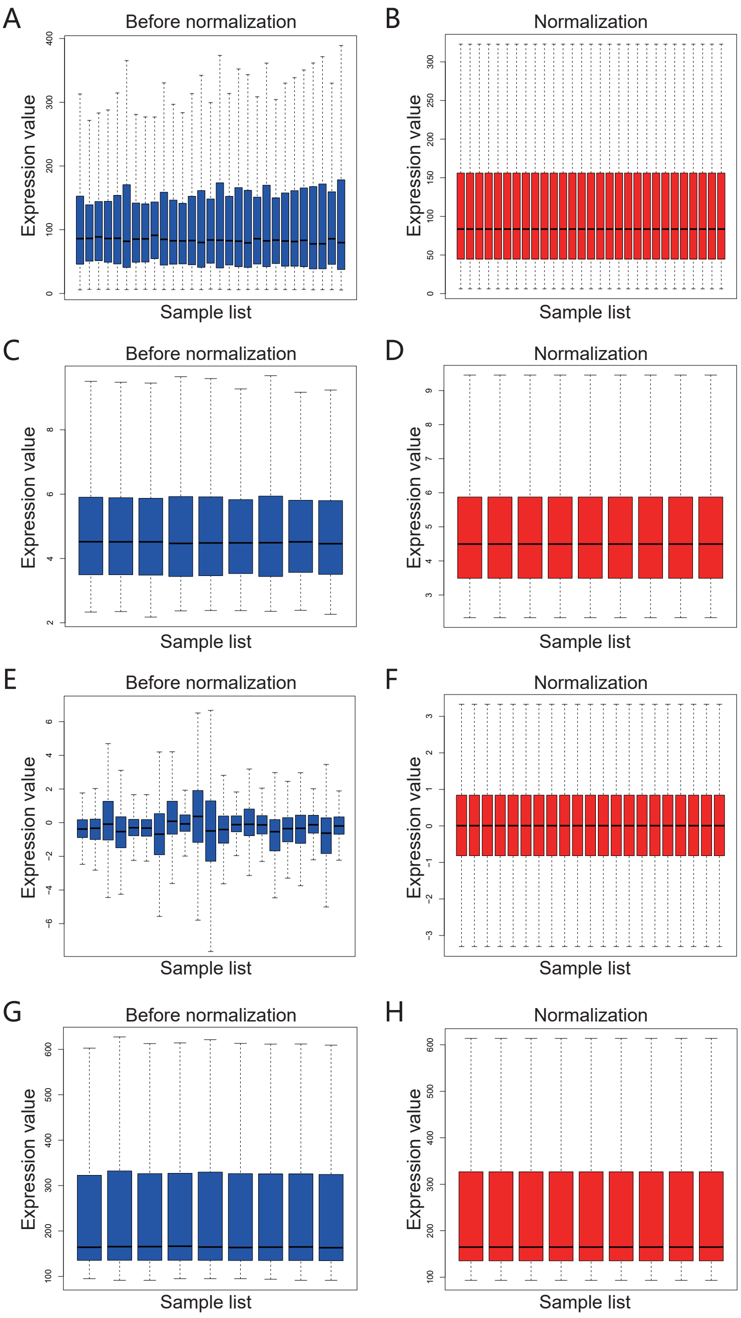

Supplementary Figure S1. Normalization of gene expression. (A–B) Normalization of the GSE3167 data set. (C–D) Normalization of the GSE7476 data set. (E–F) Normalization of the GSE40355 data set. (G–H) Normalization of the GSE65635 data set. Blue represents data before normalization, and red represents data after normalization.

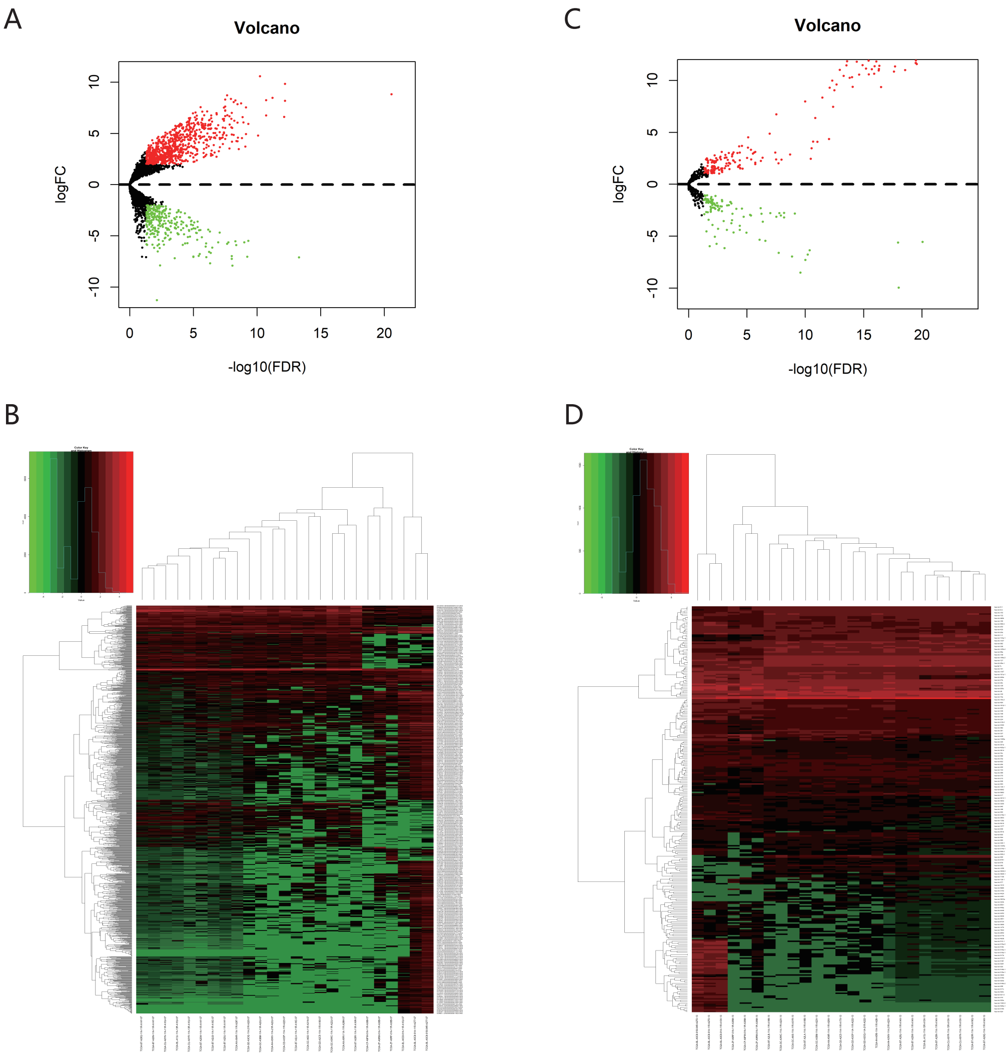

Supplementary Figure S2. DElncRNAs and DEmiRNAs in patients with NMIBC (A) Volcano plot and (B) heatmap of DElncRNAs in NMIBC and adjacent non-carcinoma bladder tissues. The DElncRNAs were identified with the thresholds of |Log2 FC| > 2.0 and adj.P-value < 0.05. (C) Volcano plot and (D) heatmap of DEmiRNAs NMIBC and adjacent non-carcinoma bladder tissues. The DEmiRNAs were identified with the thresholds of |Log2 FC| > 1.0 and adj.P-value < 0.05.
