# Supplementary material for: CDH1 overexpression predicts bladder cancer from early stage and inversely correlates with immune infiltration
Source: BMC Urol. 2022 Sep 21;22:156. doi: 10.1186/s12894-022-01103-7 (PMC9494810; doi:10.1186/s12894-022-01103-7)
Supplement: Supplementary file 2 — Additional file 2: Table S1. The clinical information of the samples in each Dataset. Table S2. Information of the integrated DEGs. Table S3. GO terms enrichment analysis of EGs. Table S4. KEGG enrichment for the top significant modules of the PPI network. Table S5. GO terms analysis for the hub genes. Table S6. Genes correlated with CDH1 based on CCLE database. Table S7. The GSEA results for CDH1 in BC cell lines. Table S8. The miRNAs related to hub genes. Table S9. The miRNAs paired with DElncRNAs and hub genes. [file 12894_2022_1103_MOESM2_ESM.pdf]

BMC Urology

CDH1 Overexpression Predicts Bladder Cancer from Early Stage and Inversely Correlates with Immune Infiltration

Tao Fan[1,2#], Liang Xue[2#], Houguang He[1,2#], Wenda Zhang[1,2], Lin Hao[1,2,3],Weiming Ma[1,3], Bingzheng Dong[1,3], Guanghui Zang[1], Conghui Han[1,2,3]， Yang Dong[1,3]*

*Correspondence:**

Yang Dong: E-mail: ydong0802@stu.suda.edu.cn

Department of Urology, Xuzhou Central Hospital,

Jiefang South Road, No. 199, Xuzhou, Jiangsu, China

Additional file

Additional file 2:

Supplementary Table S1. The clinical information of the samples in each Dataset.

Supplementary Table S2. Information of the integrated DEGs.

Supplementary Table S3. GO terms enrichment analysis of EGs.

Supplementary Table S4. KEGG enrichment for the top significant modules of the PPI network.

Supplementary Table S5. GO terms analysis for the hub genes.

Supplementary Table S6. Genes correlated with CDH1 based on CCLE database.

Supplementary Table S7. The GSEA results for CDH1 in BC cell lines.

Supplementary Table S8. The miRNAs related to hub genes.

Supplementary Table S9. The miRNAs paired with DElncRNAs and hub genes.

Supplementary Table S1. The clinical and pathological information of the samples in each dataset.

GSE3167

Sample ID	Type	Platform	Stage	Grade	Biological source	Organism	Molecule	Gender
GSM71019	Normal bladder tissues	GPL96	none	none	Normal bladder biopsy	Homo sapiens	total RNA	no information
GSM71020	Normal bladder tissues	GPL96	none	none	Normal bladder biopsy	Homo sapiens	total RNA	no information
GSM71021	Normal bladder tissues	GPL96	none	none	Normal bladder biopsy	Homo sapiens	total RNA	no information
GSM71022	Normal bladder tissues	GPL96	none	none	Normal bladder biopsy	Homo sapiens	total RNA	no information
GSM71023	Normal bladder tissues	GPL96	none	none	Normal bladder biopsy	Homo sapiens	total RNA	no information
GSM71024	Normal bladder tissues	GPL96	none	none	Normal bladder biopsy	Homo sapiens	total RNA	no information
GSM71025	Normal bladder tissues	GPL96	none	none	Normal bladder biopsy	Homo sapiens	total RNA	no information
GSM71026	Normal bladder tissues	GPL96	none	none	Normal bladder biopsy	Homo sapiens	total RNA	no information
GSM71027	Normal bladder tissues	GPL96	none	none	Normal bladder biopsy	Homo sapiens	total RNA	no information
GSM71069	Normal bladder tissues	GPL96	none	none	Normal bladder tissue	Homo sapiens	total RNA	no information
GSM71071	Normal bladder tissues	GPL96	none	none	Normal bladder tissue	Homo sapiens	total RNA	no information
GSM71073	Normal bladder tissues	GPL96	none	none	Normal bladder tissue	Homo sapiens	total RNA	no information
GSM71075	Normal bladder tissues	GPL96	none	none	Normal bladder tissue	Homo sapiens	total RNA	no information
GSM71078	Normal bladder tissues	GPL96	none	none	Normal bladder tissue	Homo sapiens	total RNA	no information
GSM71029	Tumoral bladder tissues	GPL96	Ta	G2	Tumoral bladder tissues	Homo sapiens	total RNA	no information
GSM71031	Tumoral bladder tissues	GPL96	Ta	G2	Tumoral bladder tissues	Homo sapiens	total RNA	no information
GSM71033	Tumoral bladder tissues	GPL96	Ta	G3	Tumoral bladder tissues	Homo sapiens	total RNA	no information
GSM71036	Tumoral bladder tissues	GPL96	Ta	G3	Tumoral bladder tissues	Homo sapiens	total RNA	no information
GSM71042	Tumoral bladder tissues	GPL96	Ta	G2	Tumoral bladder tissues	Homo sapiens	total RNA	no information
GSM71044	Tumoral bladder tissues	GPL96	Ta	G3	Tumoral bladder tissues	Homo sapiens	total RNA	no information
GSM71045	Tumoral bladder tissues	GPL96	Ta	G3	Tumoral bladder tissues	Homo sapiens	total RNA	no information
GSM71049	Tumoral bladder tissues	GPL96	Ta	G2	Tumoral bladder tissues	Homo sapiens	total RNA	no information
GSM71055	Tumoral bladder tissues	GPL96	Ta	G2	Tumoral bladder tissues	Homo sapiens	total RNA	no information
GSM71056	Tumoral bladder tissues	GPL96	Ta	G3	Tumoral bladder tissues	Homo sapiens	total RNA	no information
GSM71058	Tumoral bladder tissues	GPL96	Ta	G3	Tumoral bladder tissues	Homo sapiens	total RNA	no information
GSM71059	Tumoral bladder tissues	GPL96	Ta	G2	Tumoral bladder tissues	Homo sapiens	total RNA	no information
GSM71064	Tumoral bladder tissues	GPL96	Ta	G3	Tumoral bladder tissues	Homo sapiens	total RNA	no information
GSM71065	Tumoral bladder tissues	GPL96	Ta	G3	Tumoral bladder tissues	Homo sapiens	total RNA	no information
GSM71067	Tumoral bladder tissues	GPL96	Ta	G3	Tumoral bladder tissues	Homo sapiens	total RNA	no information

GSE7476

Sample ID	Type	Platform	Stage	Grade	Biological source	Organism	Molecule	Gender
GSM180991	Normal bladder tissues	GPL570	none	none	Normal bladder tissues	Homo sapiens	total RNA	no information
GSM180992	Normal bladder tissues	GPL570	none	none	Normal bladder tissues	Homo sapiens	total RNA	no information
GSM180993	Normal bladder tissues	GPL570	none	none	Normal bladder tissues	Homo sapiens	total RNA	no information
GSM180994	Tumoral bladder tissues	GPL570	Ta	low	Tumoral bladder tissues	Homo sapiens	total RNA	no information
GSM180995	Tumoral bladder tissues	GPL570	Ta	low	Tumoral bladder tissues	Homo sapiens	total RNA	no information
GSM180996	Tumoral bladder tissues	GPL570	Ta	low	Tumoral bladder tissues	Homo sapiens	total RNA	no information
GSM180997	Tumoral bladder tissues	GPL570	T1	high	Tumoral bladder tissues	Homo sapiens	total RNA	no information
GSM180998	Tumoral bladder tissues	GPL570	T1	high	Tumoral bladder tissues	Homo sapiens	total RNA	no information
GSM180999	Tumoral bladder tissues	GPL570	T1	high	Tumoral bladder tissues	Homo sapiens	total RNA	no information

GSE40355

Sample ID	Type	Platform	Stage	Grade	Biological source	Organism	Molecule	Gender
GSM991923	Normal bladder tissues	GPL13497	none	none	Normal bladder tissues	Homo sapiens	total RNA	male
GSM991924	Normal bladder tissues	GPL13497	none	none	Normal bladder tissues	Homo sapiens	total RNA	male
GSM991925	Normal bladder tissues	GPL13497	none	none	Normal bladder tissues	Homo sapiens	total RNA	male
GSM991926	Normal bladder tissues	GPL13497	none	none	Normal bladder tissues	Homo sapiens	total RNA	male
GSM991927	Normal bladder tissues	GPL13497	none	none	Normal bladder tissues	Homo sapiens	total RNA	male
GSM991928	Normal bladder tissues	GPL13497	none	none	Normal bladder tissues	Homo sapiens	total RNA	male
GSM991929	Normal bladder tissues	GPL13497	none	none	Normal bladder tissues	Homo sapiens	total RNA	male
GSM991930	Normal bladder tissues	GPL13497	none	none	Normal bladder tissues	Homo sapiens	total RNA	male
GSM991931	Tumoral bladder tissues	GPL13497	Ta	G1	Papillary urothelial carcinoma	Homo sapiens	total RNA	male
GSM991932	Tumoral bladder tissues	GPL13497	Ta	G2	Papillary urothelial carcinoma	Homo sapiens	total RNA	male
GSM991933	Tumoral bladder tissues	GPL13497	Ta	G2	Papillary urothelial carcinoma	Homo sapiens	total RNA	male
GSM991934	Tumoral bladder tissues	GPL13497	Ta	G2	Papillary urothelial carcinoma	Homo sapiens	total RNA	male
GSM991935	Tumoral bladder tissues	GPL13497	Ta	G1	Papillary urothelial carcinoma	Homo sapiens	total RNA	male
GSM991936	Tumoral bladder tissues	GPL13497	Ta	G2	Papillary urothelial carcinoma	Homo sapiens	total RNA	male
GSM991937	Tumoral bladder tissues	GPL13497	Ta	G2	Papillary urothelial carcinoma	Homo sapiens	total RNA	male
GSM991938	Tumoral bladder tissues	GPL13497	Ta	G2	Papillary urothelial carcinoma	Homo sapiens	total RNA	male
GSM991941	Tumoral bladder tissues	GPL13497	T1	G3	Papillary urothelial carcinoma	Homo sapiens	total RNA	male
GSM991942	Tumoral bladder tissues	GPL13497	T1	G3	Papillary urothelial carcinoma	Homo sapiens	total RNA	female
GSM991944	Tumoral bladder tissues	GPL13497	T1	G3	Papillary urothelial carcinoma	Homo sapiens	total RNA	male

GSM991945	Tumoral bladder tissues	GPL13497	T1	G3	Papillary urothelial carcinoma	Homo sapiens	total RNA	male
GSM991946	Tumoral bladder tissues	GPL13497	T1	G1	Papillary urothelial carcinoma	Homo sapiens	total RNA	male

GSE65635

**Sample ID**	**Type**	**Platform**	**Stage**	**Grade**	**Biological source**	**Organism**	**Molecule**	**Gender**
GSM1602279	Normal bladder tissues	GPL14951	none	none	Normal bladder tissues	Homo sapiens	total RNA	no information
GSM1602280	Normal bladder tissues	GPL14951	none	none	Normal bladder tissues	Homo sapiens	total RNA	no information
GSM1602281	Normal bladder tissues	GPL14951	none	none	Normal bladder tissues	Homo sapiens	total RNA	no information
GSM1602282	Normal bladder tissues	GPL14951	none	none	Normal bladder tissues	Homo sapiens	total RNA	no information
GSM1602283	Tumoral bladder tissues	GPL14951	T1N0 M0	high	Primary bladder cancer	Homo sapiens	total RNA	male
GSM1602285	Tumoral bladder tissues	GPL14951	T1Nx M0	low	Primary bladder cancer	Homo sapiens	total RNA	male
GSM1602286	Tumoral bladder tissues	GPL14951	T1Nx M0	low	Recurrent bladder cancer	Homo sapiens	total RNA	male
GSM1602289	Tumoral bladder tissues	GPL14951	T1N0 M0	low	Primary bladder cancer	Homo sapiens	total RNA	male
GSM1602290	Tumoral bladder tissues	GPL14951	T1N0 M0	low	Primary bladder cancer	Homo sapiens	total RNA	male

Supplementary Table S2. Information of the integrated DEGs.

Name	logFC	corrected P-value	Category
TOP2A	2.75828142	1.76043E-06	Up-regulated gene
FGFR3	2.958455257	2.79795E-06	Up-regulated gene
EEF1A2	2.95013886	1.41646E-05	Up-regulated gene
INA	2.613983016	1.66771E-05	Up-regulated gene
KRT20	2.767441453	3.45815E-05	Up-regulated gene
FXYD3	2.174890144	0.000112053	Up-regulated gene
TOX3	2.598046427	0.000142595	Up-regulated gene
CTSE	3.088860857	0.000152868	Up-regulated gene
PAFAH1B3	2.481144635	0.000156413	Up-regulated gene
RAB25	2.632083474	0.000160018	Up-regulated gene
WDR72	2.841329513	0.000300398	Up-regulated gene
SDC1	2.533057033	0.000343272	Up-regulated gene
CDC20	2.571627504	0.000343272	Up-regulated gene
PTK6	2.209420828	0.00039067	Up-regulated gene
EPN3	2.073337532	0.00051733	Up-regulated gene
PTPRR	2.297963479	0.000716274	Up-regulated gene
KRT7	3.133837119	0.000832168	Up-regulated gene
EHF	1.984898714	0.000926339	Up-regulated gene
MAPK13	2.170484697	0.001039788	Up-regulated gene
LAD1	2.433706595	0.001280225	Up-regulated gene
RNF128	2.087766727	0.001662906	Up-regulated gene
CDH1	2.485135004	0.002176638	Up-regulated gene
CXorf57	1.925732898	0.002202512	Up-regulated gene
MDK	2.150009434	0.002832358	Up-regulated gene
SPAG4	2.166092628	0.003091896	Up-regulated gene
MSMB	2.12565375	0.003125549	Up-regulated gene
UPK2	2.456614929	0.003551181	Up-regulated gene
CYP2J2	2.238102667	0.003937891	Up-regulated gene
PPARG	2.020323637	0.004486829	Up-regulated gene
FCRLB	2.572514165	0.005274301	Up-regulated gene
POF1B	1.6700673	0.005701554	Up-regulated gene
FER1L4	2.104946629	0.006598707	Up-regulated gene
PLA2G2F	1.859837071	0.006658087	Up-regulated gene
CXADR	2.047607201	0.007730211	Up-regulated gene
TCN1	2.796192137	0.008428322	Up-regulated gene
SCNN1B	2.235393694	0.00863362	Up-regulated gene
EPCAM	1.912244864	0.00863362	Up-regulated gene
HN1	1.877567438	0.009227549	Up-regulated gene
JUP	1.769882714	0.0093807	Up-regulated gene
UBE2C	2.825670989	0.010012446	Up-regulated gene
ERBB3	1.824410778	0.012287691	Up-regulated gene

FASN	1.937130747	0.014605452	Up-regulated gene
MCM4	1.459834514	0.015596241	Up-regulated gene
C1orf106	1.983174066	0.015709359	Up-regulated gene
SSH3	1.69009767	0.018613616	Up-regulated gene
SPINT1	1.86739832	0.019800302	Up-regulated gene
KRT8	2.21124494	0.022195614	Up-regulated gene
HMGCS2	2.65279363	0.023501779	Up-regulated gene
CD24	2.22453246	0.027676857	Up-regulated gene
SEMA3F	1.712953439	0.029223789	Up-regulated gene
GJB6	2.271054898	0.029937906	Up-regulated gene
KRT19	2.425795387	0.02995112	Up-regulated gene
MOCOS	1.467073749	0.029953209	Up-regulated gene
CRH	2.224470971	0.030718055	Up-regulated gene
CLDN7	1.907772606	0.033807222	Up-regulated gene
CD46	1.520117658	0.035868057	Up-regulated gene
EVPL	1.853270109	0.038021705	Up-regulated gene
NUSAP1	2.258269525	0.03824228	Up-regulated gene
IGFBP3	2.083903574	0.038686308	Up-regulated gene
TP63	2.021631607	0.040732466	Up-regulated gene
F11R	1.736810403	0.044321778	Up-regulated gene
RAPGEFL1	2.07273933	0.046075645	Up-regulated gene
SYNM	-5.614549416	4.76313E-09	Down-regulated gene
PCP4	-5.848602236	2.17337E-08	Down-regulated gene
CNN1	-5.817880216	2.66833E-08	Down-regulated gene
RGS2	-4.2584057	4.00416E-06	Down-regulated gene
MYH11	-4.677222155	4.23792E-06	Down-regulated gene
TAGLN	-4.323101021	5.00088E-06	Down-regulated gene
MFAP4	-3.95475996	5.56383E-06	Down-regulated gene
LMOD1	-3.889929755	1.04636E-05	Down-regulated gene
PTGS1	-4.680686384	1.04636E-05	Down-regulated gene
FAM129A	-3.819645985	1.09418E-05	Down-regulated gene
ACTC1	-4.978789446	1.14363E-05	Down-regulated gene
PDLIM3	-3.840153058	1.95098E-05	Down-regulated gene
ACTG2	-4.804323857	5.87492E-05	Down-regulated gene
CRYAB	-3.966264984	7.78733E-05	Down-regulated gene
CASQ2	-4.335641609	8.44276E-05	Down-regulated gene
SRPX	-4.179494683	8.90213E-05	Down-regulated gene
CAV1	-3.435467411	9.13871E-05	Down-regulated gene
SPARCL1	-3.533486343	9.62601E-05	Down-regulated gene
SPON1	-3.411362818	0.000112053	Down-regulated gene
FERMT2	-3.538472258	0.000114863	Down-regulated gene
FXYD6	-3.548211134	0.000199605	Down-regulated gene
EGR1	-3.408483025	0.000221949	Down-regulated gene
EFEMP1	-3.482821297	0.000231392	Down-regulated gene

COL6A2	-3.122252424	0.000277667	Down-regulated gene
CFD	-4.213352956	0.000300398	Down-regulated gene
FGL2	-3.173005758	0.000324337	Down-regulated gene
MYL9	-4.229015965	0.000330559	Down-regulated gene
FHL1	-3.951841942	0.000337898	Down-regulated gene
CTGF	-3.494955191	0.000343272	Down-regulated gene
P2RX1	-3.262845378	0.00039067	Down-regulated gene
HSD17B6	-3.30716477	0.000458629	Down-regulated gene
BAG2	-3.113390544	0.000491522	Down-regulated gene
DES	-4.287190987	0.00053509	Down-regulated gene
LUM	-3.005187376	0.00057198	Down-regulated gene
MGP	-3.455300555	0.00063085	Down-regulated gene
SLIT2	-3.430732895	0.000716274	Down-regulated gene
C7	-3.181595302	0.000762101	Down-regulated gene
OLFML3	-3.2557932	0.000797889	Down-regulated gene
RGS1	-3.233700577	0.000886287	Down-regulated gene
AEBP1	-2.935017541	0.000912841	Down-regulated gene
FOXF1	-3.270044254	0.001025065	Down-regulated gene
TUBB6	-2.82601284	0.001039788	Down-regulated gene
DPYSL3	-3.490287102	0.001084908	Down-regulated gene
RASL12	-3.271809461	0.001084908	Down-regulated gene
CYR61	-3.591700739	0.001179537	Down-regulated gene
CILP	-3.465393115	0.001212414	Down-regulated gene
CLIC4	-2.787473931	0.001350842	Down-regulated gene
AOC3	-3.210490099	0.00142434	Down-regulated gene
ALDH2	-2.789798924	0.001705406	Down-regulated gene
MAP1B	-2.906843326	0.001770678	Down-regulated gene
PTGIS	-3.898858476	0.001837805	Down-regulated gene
PRUNE2	-3.432449567	0.001953903	Down-regulated gene
STON1	-2.879074983	0.001953903	Down-regulated gene
MYLK	-4.030455441	0.001994904	Down-regulated gene
TNS1	-2.75272931	0.002026151	Down-regulated gene
ITM2A	-2.797898078	0.002176638	Down-regulated gene
DUSP1	-2.964960268	0.002202512	Down-regulated gene
DPYSL2	-2.777292729	0.002335356	Down-regulated gene
REEP1	-3.269472591	0.002390143	Down-regulated gene
DCN	-3.504637221	0.002417894	Down-regulated gene
RAMP1	-2.982127846	0.002739375	Down-regulated gene
GAS6	-2.877437693	0.002863873	Down-regulated gene
MXRA7	-2.64230543	0.003025406	Down-regulated gene
JAM3	-3.314403591	0.003091896	Down-regulated gene
CALD1	-3.390887597	0.003228158	Down-regulated gene
EMP3	-2.416986341	0.003262915	Down-regulated gene
ITGA5	-3.011833537	0.003477393	Down-regulated gene

GEM	-3.550565155	0.003557459	Down-regulated gene
TMOD1	-2.810466123	0.003626138	Down-regulated gene
LMCD1	-2.659427745	0.003740789	Down-regulated gene
FAM107A	-2.555570217	0.003740789	Down-regulated gene
SLC2A3	-2.482887877	0.004531307	Down-regulated gene
HSPB6	-3.497428956	0.004531307	Down-regulated gene
COX7A1	-3.155676005	0.004758682	Down-regulated gene
SMTN	-2.91597999	0.005648701	Down-regulated gene
PLSCR4	-2.928267103	0.005754777	Down-regulated gene
RARRES2	-2.423916017	0.006081982	Down-regulated gene
CRISPLD2	-2.742000444	0.006365148	Down-regulated gene
ADH1B	-3.453805203	0.006481138	Down-regulated gene
FLNC	-4.468419628	0.006481138	Down-regulated gene
TMEM158	-2.251729737	0.008779332	Down-regulated gene
CTSK	-2.447504642	0.008852875	Down-regulated gene
COLEC12	-2.611120882	0.009001348	Down-regulated gene
COL6A3	-2.863971631	0.0093807	Down-regulated gene
CYP1B1	-2.286354518	0.00953575	Down-regulated gene
MT2A	-2.490181911	0.010257388	Down-regulated gene
JUN	-2.631682906	0.010257388	Down-regulated gene
ACTA2	-3.385464649	0.010590934	Down-regulated gene
ISLR	-2.309506662	0.011282364	Down-regulated gene
TGFB1I1	-3.079273027	0.011460386	Down-regulated gene
EMP1	-2.034424978	0.011640506	Down-regulated gene
FOSB	-3.499072464	0.011914658	Down-regulated gene
ZCCHC24	-2.786583835	0.012100096	Down-regulated gene
TPSAB1	-2.747938008	0.012193623	Down-regulated gene
RCAN2	-3.174504289	0.013080145	Down-regulated gene
BNC2	-2.271355437	0.013258594	Down-regulated gene
MCAM	-2.123733276	0.013662637	Down-regulated gene
GHR	-2.407658615	0.013662637	Down-regulated gene
PCOLCE2	-3.932884317	0.013991666	Down-regulated gene
C1S	-2.764645118	0.014391847	Down-regulated gene
RNASE4	-2.60905055	0.014391847	Down-regulated gene
BIN1	-2.66206538	0.014605452	Down-regulated gene
DPT	-3.458979282	0.014605452	Down-regulated gene
TCF21	-2.563214719	0.015039785	Down-regulated gene
CKB	-2.460661753	0.015371838	Down-regulated gene
SOD3	-2.102946658	0.01593744	Down-regulated gene
TPM1	-3.078292519	0.016284208	Down-regulated gene
HSPB8	-3.105729271	0.016636603	Down-regulated gene
WFDC1	-2.668989592	0.017358522	Down-regulated gene
CPA3	-2.847358378	0.017852684	Down-regulated gene
SFRP2	-3.688583031	0.018618538	Down-regulated gene

PAMR1	-2.530229809	0.019398618	Down-regulated gene
NEXN	-3.299697435	0.019768853	Down-regulated gene
PLN	-3.730645179	0.020901972	Down-regulated gene
CLIP3	-3.021607145	0.020901972	Down-regulated gene
AHNAK2	-2.641780494	0.021184427	Down-regulated gene
COL15A1	-2.342331514	0.021326723	Down-regulated gene
MEF2C	-1.988943287	0.021757916	Down-regulated gene
TPST1	-2.045137572	0.023857222	Down-regulated gene
LHFP	-2.0456983	0.024169077	Down-regulated gene
PALLD	-3.137944304	0.024483979	Down-regulated gene
GSTM5	-2.079779788	0.026104897	Down-regulated gene
SERPINA3	-2.617354882	0.026271302	Down-regulated gene
FBLN5	-2.813786296	0.026775294	Down-regulated gene
COL3A1	-2.236150451	0.027286502	Down-regulated gene
SDC2	-2.064064391	0.02745852	Down-regulated gene
TPM2	-3.30731718	0.028507773	Down-regulated gene
FILIP1L	-2.540446362	0.029404881	Down-regulated gene
DIXDC1	-2.250776515	0.029404881	Down-regulated gene
PMP22	-2.646077027	0.029586813	Down-regulated gene
IGFBP6	-3.406917993	0.030718055	Down-regulated gene
SERPINF1	-2.489974117	0.031072835	Down-regulated gene
PNMA1	-2.059603999	0.031262447	Down-regulated gene
JAM2	-2.372389136	0.032614144	Down-regulated gene
ANXA5	-2.162247184	0.035032738	Down-regulated gene
RBPMS2	-3.582273101	0.035684817	Down-regulated gene
ATF3	-2.158602306	0.035868057	Down-regulated gene
ACTN1	-2.306227423	0.036291274	Down-regulated gene
DDR2	-2.303899189	0.036291274	Down-regulated gene
FSTL1	-2.247994927	0.037365703	Down-regulated gene
FLNA	-2.944060131	0.038021705	Down-regulated gene
C2orf40	-4.237020424	0.041159424	Down-regulated gene
FMOD	-1.914436215	0.041667463	Down-regulated gene
ADAMTSL3	-2.19398999	0.043342329	Down-regulated gene
TCEAL2	-2.537269562	0.043342329	Down-regulated gene
KCTD12	-1.904384126	0.043585654	Down-regulated gene
ZEB2	-1.668315627	0.044321778	Down-regulated gene
GNG11	-2.073568186	0.046330382	Down-regulated gene
LIMS2	-2.733231247	0.046843023	Down-regulated gene
LGALS1	-2.296343584	0.047100933	Down-regulated gene
MT1E	-2.121319032	0.048936247	Down-regulated gene

Note: $|logFC| \geqslant 1$ and corrected P-value < 0.05

Supplementary Table S3. GO terms analysis for the integrated DEGs. Through GO classification for the upregulated and downregulated integrated DEGs, 32 and 173 remarkably (adj. P-value ≤0.05) enriched GO terms were obtained respectively, including biological process (BP), cellular component (CC) and molecular function (MF).

Group	GO terms	GO ID	Description	Count	adj. P-value	FDR	Gene ID
Upregulated DEGs	BP	GO:0070268	cornification	6	2.08E-03	1.70E-03	KRT20/KRT7/JUP/KRT8/KRT19/EVPL
Upregulated DEGs	BP	GO:0002064	epithelial cell development	6	2.00E-02	1.63E-02	RAB25/SDC1/POF1B/FASN/TP63/F11R
Upregulated DEGs	BP	GO:0030216	keratinocyte differentiation	7	2.00E-02	1.63E-02	KRT20/KRT7/JUP/KRT8/KRT19/EVPL/TP63
Upregulated DEGs	BP	GO:0031424	keratinization	6	2.52E-02	2.05E-02	KRT20/KRT7/JUP/KRT8/KRT19/EVPL
Upregulated DEGs	BP	GO:0043627	response to estrogen	4	2.52E-02	2.05E-02	PPARG/CD24/KRT19/CRH
Upregulated DEGs	BP	GO:0009913	epidermal cell differentiation	7	2.56E-02	2.09E-02	KRT20/KRT7/JUP/KRT8/KRT19/EVPL/TP63
Upregulated DEGs	BP	GO:0038128	ERBB2 signaling pathway	3	3.05E-02	2.48E-02	PTK6/PTPRR/ERBB3
Upregulated DEGs	BP	GO:0097327	response to antineoplastic agent	4	4.55E-02	3.71E-02	MAPK13/CDH1/JUP/CRH
Upregulated DEGs	BP	GO:0043588	skin development	7	5.00E-02	4.08E-02	KRT20/KRT7/JUP/KRT8/KRT19/EVPL/TP63
Upregulated DEGs	CC	GO:0016327	apicolateral plasma membrane	5	4.22E-07	3.16E-07	CXADR/JUP/KRT8/KRT19/CLDN7
Upregulated DEGs	CC	GO:0043296	apical junction complex	7	1.48E-05	1.11E-05	CDH1/POF1B/CXADR/EPCAM/JUP/CLDN7/F11R
Upregulated DEGs	CC	GO:0005911	cell-cell junction	10	2.90E-05	2.17E-05	CDH1/POF1B/CXADR/EPCAM/JUP/KRT8/GJB6/CLDN7/EVPL/F11R
Upregulated DEGs	CC	GO:0016328	lateral plasma membrane	5	3.79E-05	2.83E-05	CDH1/EPCAM/JUP/ERBB3/CLDN7
Upregulated DEGs	CC	GO:0045111	intermediate filament cytoskeleton	7	3.32E-04	2.49E-04	INA/KRT20/KRT7/JUP/KRT8/KRT19/EVPL
Upregulated DEGs	CC	GO:0005923	bicellular tight junction	5	8.23E-04	6.16E-04	POF1B/CXADR/EPCAM/CLDN7/F11R
Upregulated DEGs	CC	GO:0070160	tight junction	5	8.23E-04	6.16E-04	POF1B/CXADR/EPCAM/CLDN7/F11R
Upregulated DEGs	CC	GO:0005882	intermediate filament	6	8.23E-04	6.16E-04	INA/KRT20/KRT7/JUP/KRT8/KRT19
Upregulated DEGs	CC	GO:0030057	desmosome	3	1.03E-03	7.74E-04	POF1B/JUP/EVPL
Upregulated DEGs	CC	GO:0016010	dystrophin-associated glycoprotein complex	2	1.48E-02	1.11E-02	KRT8/KRT19
Upregulated DEGs	CC	GO:0090665	glycoprotein complex	2	1.48E-02	1.11E-02	KRT8/KRT19
Upregulated DEGs	CC	GO:0043034	costamere	2	1.72E-02	1.29E-02	KRT8/KRT19
Upregulated DEGs	CC	GO:0005680	anaphase-promoting complex	2	2.03E-02	1.52E-02	CDC20/UBE2C
Upregulated DEGs	CC	GO:0005913	cell-cell adherens junction	3	2.03E-02	1.52E-02	CDH1/CXADR/JUP
Upregulated DEGs	CC	GO:0016323	basolateral plasma membrane	4	3.53E-02	2.65E-02	CXADR/EPCAM/ERBB3/CLDN7
Upregulated DEGs	CC	GO:0019897	extrinsic component of plasma membrane	3	3.66E-02	2.74E-02	EPN3/CDH1/JUP
Upregulated DEGs	CC	GO:0030018	Z disc	3	3.68E-02	2.75E-02	JUP/KRT8/KRT19
Upregulated DEGs	CC	GO:0005912	adherens junction	6	3.68E-02	2.75E-02	FGFR3/CDH1/POF1B/CXADR/JUP/CD46
Upregulated DEGs	CC	GO:0098552	side of membrane	5	4.08E-02	3.06E-02	EEF1A2/SDC1/CDH1/SCNN1B/JUP
Upregulated DEGs	CC	GO:0031674	I band	3	4.18E-02	3.13E-02	JUP/KRT8/KRT19
Upregulated DEGs	CC	GO:0000152	nuclear ubiquitin ligase complex	2	4.33E-02	3.25E-02	CDC20/UBE2C
Upregulated DEGs	MF	GO:0050839	cell adhesion molecule binding	9	2.32E-03	1.99E-03	LAD1/CDH1/CXADR/JUP/FASN/CLDN7/CD46/EVPL/F11R
Upregulated DEGs	MF	GO:0045296	cadherin binding	7	4.63E-03	3.98E-03	LAD1/CDH1/JUP/FASN/CD46/EVPL/F11R
Downregulated DEGs	BP	GO:0006936	muscle contraction	24	3.97E-13	3.25E-13	SYNM/CNN1/RGS2/MYH11/LMOD1/ACTC1/ACTG2/CRYAB/CASQ2/CAV1/MYL9/P2

							RX1/DES/MYLK/CALD1/TMOD1/HSPB6/SMTN/ACTA2/BIN1/TPM1/PLN/TPM2/FLNA
Downregulated DEGs	BP	GO:0003012	muscle system process	26	8.90E-13	7.30E-13	SYNM/CNN1/RGS2/MYH11/LMOD1/ACTC1/ACTG2/CRYAB/CASQ2/CAV1/MYL9/P2RX1/DES/MYLK/CALD1/TMOD1/LMCD1/HSPB6/SMTN/ACTA2/BIN1/TPM1/PLN/MEF2C/TPM2/FLNA
Downregulated DEGs	BP	GO:0030198	extracellular matrix organization	21	1.00E-10	8.21E-11	MYH11/MFAP4/COL6A2/LUM/AEBP1/FOXF1/DCN/GAS6/JAM3/ITGA5/CRISPLD2/CTSK/COL6A3/CYP1B1/TPSAB1/SFRP2/FBLN5/COL3A1/JAM2/DDR2/FMOD
Downregulated DEGs	BP	GO:0043062	extracellular structure organization	21	1.29E-09	1.06E-09	MYH11/MFAP4/COL6A2/LUM/AEBP1/FOXF1/DCN/GAS6/JAM3/ITGA5/CRISPLD2/CTSK/COL6A3/CYP1B1/TPSAB1/SFRP2/FBLN5/COL3A1/JAM2/DDR2/FMOD
Downregulated DEGs	BP	GO:0030199	collagen fibril organization	7	2.35E-05	1.93E-05	LUM/AEBP1/CYP1B1/SFRP2/COL3A1/DDR2/FMOD
Downregulated DEGs	BP	GO:0006937	regulation of muscle contraction	11	2.35E-05	1.93E-05	CNN1/RGS2/CASQ2/CAV1/MYL9/P2RX1/HSPB6/BIN1/TPM1/PLN/FLNA
Downregulated DEGs	BP	GO:0007517	muscle organ development	16	3.10E-05	2.54E-05	RGS2/TAGLN/ACTC1/CRYAB/CAV1/EGR1/FHL1/MYLK/DCN/SMTN/COL6A3/TCF21/TPM1/MEF2C/COL3A1/ATF3
Downregulated DEGs	BP	GO:0070252	actin-mediated cell contraction	9	8.00E-05	6.56E-05	ACTC1/CAV1/DES/TMOD1/BIN1/TPM1/PLN/TPM2/FLNA
Downregulated DEGs	BP	GO:0090257	regulation of muscle system process	12	1.55E-04	1.27E-04	CNN1/RGS2/CASQ2/CAV1/MYL9/P2RX1/LMCD1/HSPB6/BIN1/TPM1/PLN/FLNA
Downregulated DEGs	BP	GO:0002576	platelet degranulation	9	1.60E-04	1.31E-04	CFD/P2RX1/GAS6/RARRES2/ISLR/SERPINA3/ANXA5/ACTN1/FLNA
Downregulated DEGs	BP	GO:0030048	actin filament-based movement	9	1.88E-04	1.54E-04	ACTC1/CAV1/DES/TMOD1/BIN1/TPM1/PLN/TPM2/FLNA
Downregulated DEGs	BP	GO:0032060	bleb assembly	4	2.20E-04	1.80E-04	MYLK/EMP3/EMP1/PMP22
Downregulated DEGs	BP	GO:0006939	smooth muscle contraction	8	2.86E-04	2.35E-04	CNN1/RGS2/MYH11/CAV1/P2RX1/MYLK/SMTN/ACTA2
Downregulated DEGs	BP	GO:0051271	negative regulation of cellular component movement	13	8.85E-04	7.26E-04	SLIT2/DPYSL3/CLIC4/DUSP1/DCN/CYP1B1/BIN1/TPM1/SFRP2/MEF2C/COL3A1/SERPINF1/ACTN1
Downregulated DEGs	BP	GO:0006941	striated muscle contraction	9	1.12E-03	9.18E-04	SYNM/RGS2/ACTC1/CASQ2/CAV1/BIN1/TPM1/PLN/FLNA
Downregulated DEGs	BP	GO:0060048	cardiac muscle contraction	8	1.29E-03	1.06E-03	RGS2/ACTC1/CASQ2/CAV1/BIN1/TPM1/PLN/FLNA
Downregulated DEGs	BP	GO:0060537	muscle tissue development	13	1.45E-03	1.19E-03	RGS2/MYH11/ACTC1/CAV1/EGR1/MYLK/DCN/TCF21/TPM1/PLN/MEF2C/COL3A1/ATF3
Downregulated DEGs	BP	GO:0010038	response to metal ion	12	1.45E-03	1.19E-03	CASQ2/CAV1/CLIC4/DUSP1/MT2A/JUN/FOSB/SOD3/PLN/MEF2C/SERPINF1/MT1E
Downregulated DEGs	BP	GO:0030049	muscle filament sliding	5	1.45E-03	1.19E-03	ACTC1/DES/TMOD1/TPM1/TPM2
Downregulated DEGs	BP	GO:0033275	actin-myosin filament sliding	5	1.45E-03	1.19E-03	ACTC1/DES/TMOD1/TPM1/TPM2
Downregulated DEGs	BP	GO:0032970	regulation of actin	12	1.56E-03	1.28E-03	LMOD1/CAV1/SLIT2/JAM3/TMOD1/FAM1

			filament-based process				07A/BIN1/TPM1/PLN/MEF2C/DIXDC1/FLNA
Downregulated DEGs	BP	GO:0051493	regulation of cytoskeleton organization	14	1.93E-03	1.58E-03	LMOD1/SLIT2/CLIC4/MAP1B/JAM3/TMOD1/FAM107A/BIN1/TPM1/NEXN/CLIP3/MEF2C/DIXDC1/FLNA
Downregulated DEGs	BP	GO:1902903	regulation of supramolecular fiber organization	11	3.19E-03	2.62E-03	LMOD1/CRYAB/SLIT2/AEBP1/MAP1B/TMOD1/BIN1/TPM1/CLIP3/MEF2C/FLNA
Downregulated DEGs	BP	GO:0060485	mesenchyme development	10	3.19E-03	2.62E-03	ACTC1/ACTG2/FOXF1/ACTA2/TGFB1I1/BNC2/TCF21/SFRP2/MEF2C/ZEB2
Downregulated DEGs	BP	GO:0055001	muscle cell development	8	3.19E-03	2.62E-03	RGS2/MYH11/ACTC1/CASQ2/FLNC/BIN1/TPM1/MEF2C
Downregulated DEGs	BP	GO:0055117	regulation of cardiac muscle contraction	6	3.55E-03	2.91E-03	RGS2/CASQ2/CAV1/BIN1/PLN/FLNA
Downregulated DEGs	BP	GO:0031589	cell-substrate adhesion	11	3.55E-03	2.91E-03	FERMT2/FOXF1/GAS6/JAM3/ITGA5/FAM107A/FBLN5/COL3A1/ACTN1/FLNA/LGALS1
Downregulated DEGs	BP	GO:0007178	transmembrane receptor protein serine/threonine kinase signaling pathway	11	3.55E-03	2.91E-03	CAV1/FERMT2/EGR1/CILP/JUN/TGFB1I1/SFRP2/COL3A1/RBPMS2/FSTL1/FMOD
Downregulated DEGs	BP	GO:0034329	cell junction assembly	9	3.55E-03	2.91E-03	CAV1/FERMT2/TNS1/ITGA5/FAM107A/FLNC/ACTN1/FLNA/LIMS2
Downregulated DEGs	BP	GO:0007015	actin filament organization	11	3.55E-03	2.91E-03	LMOD1/ACTC1/PDLIM3/SLIT2/DPYSL3/TMOD1/FAM107A/BIN1/TPM1/ACTN1/FLNA
Downregulated DEGs	BP	GO:0030239	myofibril assembly	5	3.55E-03	2.91E-03	MYH11/ACTC1/CASQ2/TPM1/MEF2C
Downregulated DEGs	BP	GO:1904063	negative regulation of cation transmembrane transport	6	3.84E-03	3.15E-03	RGS2/CASQ2/CAV1/GEM/BIN1/PLN
Downregulated DEGs	BP	GO:0030336	negative regulation of cell migration	11	3.84E-03	3.15E-03	SLIT2/DPYSL3/CLIC4/DUSP1/DCN/CYP1B1/TPM1/SFRP2/MEF2C/COL3A1/SERPINF1
Downregulated DEGs	BP	GO:0060047	heart contraction	10	3.84E-03	3.15E-03	RGS2/ACTC1/CASQ2/CAV1/FXYD6/DES/BIN1/TPM1/PLN/FLNA
Downregulated DEGs	BP	GO:0051592	response to calcium ion	7	3.84E-03	3.15E-03	CASQ2/CAV1/CLIC4/DUSP1/JUN/FOSB/MEF2C
Downregulated DEGs	BP	GO:0085029	extracellular matrix assembly	4	4.10E-03	3.37E-03	MYH11/MFAP4/GAS6/FBLN5
Downregulated DEGs	BP	GO:0003015	heart process	10	4.75E-03	3.90E-03	RGS2/ACTC1/CASQ2/CAV1/FXYD6/DES/BIN1/TPM1/PLN/FLNA
Downregulated DEGs	BP	GO:2000146	negative regulation of cell motility	11	5.15E-03	4.22E-03	SLIT2/DPYSL3/CLIC4/DUSP1/DCN/CYP1B1/TPM1/SFRP2/MEF2C/COL3A1/SERPINF1
Downregulated DEGs	BP	GO:1903522	regulation of blood circulation	10	5.67E-03	4.65E-03	RGS2/CASQ2/CAV1/FXYD6/P2RX1/DES/BIN1/TPM1/PLN/FLNA
Downregulated DEGs	BP	GO:0006942	regulation of striated muscle contraction	6	6.08E-03	4.99E-03	RGS2/CASQ2/CAV1/BIN1/PLN/FLNA
Downregulated DEGs	BP	GO:0032956	regulation of actin cytoskeleton organization	10	6.13E-03	5.03E-03	LMOD1/SLIT2/JAM3/TMOD1/FAM107A/BIN1/TPM1/MEF2C/DIXDC1/FLNA
Downregulated DEGs	BP	GO:0034766	negative regulation of ion transmembrane transport	6	6.13E-03	5.03E-03	RGS2/CASQ2/CAV1/GEM/BIN1/PLN
Downregulated DEGs	BP	GO:0090130	tissue migration	11	6.78E-03	5.56E-03	ACTC1/ACTG2/SLIT2/FOXF1/DCN/CYP1B1/JUN/ACTA2/MEF2C/SERPINF1/ZEB2
Downregulated DEGs	BP	GO:0035637	multicellular organismal	8	7.09E-03	5.81E-03	CASQ2/CAV1/FXYD6/P2RX1/JAM3/BIN1/

			signaling				PLN/FLNA
Downregulated DEGs	BP	GO:0048566	embryonic digestive tract development	4	7.09E-03	5.81E-03	FOXF1/RARRES2/TCF21/RBPMS2
Downregulated DEGs	BP	GO:0055002	striated muscle cell development	7	7.09E-03	5.81E-03	RGS2/MYH11/ACTC1/CASQ2/FLNC/TPM1/MEF2C
Downregulated DEGs	BP	GO:0008016	regulation of heart contraction	9	7.12E-03	5.84E-03	RGS2/CASQ2/CAV1/FXYD6/DES/BIN1/TPM1/PLN/FLNA
Downregulated DEGs	BP	GO:0014706	striated muscle tissue development	11	8.26E-03	6.77E-03	RGS2/MYH11/ACTC1/CAV1/EGR1/DCN/TCF21/TPM1/PLN/MEF2C/ATF3
Downregulated DEGs	BP	GO:0040013	negative regulation of locomotion	11	8.28E-03	6.79E-03	SLIT2/DPYSL3/CLIC4/DUSP1/DCN/CYP1B1/TPM1/SFRP2/MEF2C/COL3A1/SERPINF1
Downregulated DEGs	BP	GO:0010975	regulation of neuron projection development	12	1.11E-02	9.09E-03	RGS2/SLIT2/DPYSL3/MAP1B/DPYSL2/SFRP2/MEF2C/SDC2/PMP22/SERPINF1/ZEB2/LGALS1
Downregulated DEGs	BP	GO:0086103	G protein-coupled receptor signaling pathway involved in heart process	3	1.13E-02	9.25E-03	RGS2/CAV1/PLN
Downregulated DEGs	BP	GO:0009410	response to xenobiotic stimulus	9	1.16E-02	9.50E-03	RGS2/PTGS1/CASQ2/EGR1/AOC3/CYP1B1/FOSB/GHR/SERPINF1
Downregulated DEGs	BP	GO:1903170	negative regulation of calcium ion transmembrane transport	4	1.16E-02	9.50E-03	CASQ2/GEM/BIN1/PLN
Downregulated DEGs	BP	GO:0071466	cellular response to xenobiotic stimulus	7	1.27E-02	1.04E-02	PTGS1/CASQ2/EGR1/AOC3/CYP1B1/GHR/SERPINF1
Downregulated DEGs	BP	GO:0048678	response to axon injury	5	1.27E-02	1.04E-02	DPYSL3/MAP1B/JAM3/JUN/LGALS1
Downregulated DEGs	BP	GO:0034330	cell junction organization	9	1.30E-02	1.07E-02	CAV1/FERMT2/TNS1/ITGA5/FAM107A/FLNC/ACTN1/FLNA/LIMS2
Downregulated DEGs	BP	GO:0086004	regulation of cardiac muscle cell contraction	4	1.30E-02	1.07E-02	CAV1/BIN1/PLN/FLNA
Downregulated DEGs	BP	GO:0071248	cellular response to metal ion	7	1.34E-02	1.10E-02	CLIC4/MT2A/JUN/FOSB/MEF2C/SERPINF1/MT1E
Downregulated DEGs	BP	GO:0048557	embryonic digestive tract morphogenesis	3	1.40E-02	1.15E-02	FOXF1/TCF21/RBPMS2
Downregulated DEGs	BP	GO:0034763	negative regulation of transmembrane transport	6	1.48E-02	1.21E-02	RGS2/CASQ2/CAV1/GEM/BIN1/PLN
Downregulated DEGs	BP	GO:1902904	negative regulation of supramolecular fiber organization	6	1.48E-02	1.21E-02	LMOD1/CRYAB/SLIT2/MAP1B/TMOD1/CLIP3
Downregulated DEGs	BP	GO:0051258	protein polymerization	8	1.75E-02	1.44E-02	LMOD1/CASQ2/SLIT2/MAP1B/TMOD1/FAM107A/BIN1/CLIP3
Downregulated DEGs	BP	GO:0071560	cellular response to transforming growth factor beta stimulus	8	1.77E-02	1.45E-02	CAV1/FERMT2/CILP/JUN/TGFB1I1/MEF2C/COL3A1/FMOD
Downregulated DEGs	BP	GO:1903115	regulation of actin filament-based movement	4	1.79E-02	1.47E-02	CAV1/BIN1/PLN/FLNA
Downregulated DEGs	BP	GO:0042692	muscle cell differentiation	10	1.79E-02	1.47E-02	RGS2/MYH11/ACTC1/CASQ2/FOXF1/FLNC/BIN1/TPM1/MEF2C/RBPMS2
Downregulated DEGs	BP	GO:1901379	regulation of potassium ion transmembrane transport	5	1.79E-02	1.47E-02	CASQ2/CAV1/FHL1/BIN1/FLNA
Downregulated DEGs	BP	GO:0048546	digestive tract morphogenesis	4	1.86E-02	1.53E-02	FOXF1/TCF21/SFRP2/RBPMS2

Downregulated DEGs	BP	GO:0071559	response to transforming growth factor beta	8	1.94E-02	1.59E-02	CAV1/FERMT2/CILP/JUN/TGFB1I1/MEF2C/COL3A1/FMOD
Downregulated DEGs	BP	GO:0048565	digestive tract development	6	1.99E-02	1.63E-02	FOXF1/RARRES2/TCF21/SFRP2/COL3A1/RBPMS2
Downregulated DEGs	BP	GO:0061448	connective tissue development	8	2.11E-02	1.73E-02	EGR1/EFEMP1/LUM/MGP/CTSK/ACTA2/SFRP2/MEF2C
Downregulated DEGs	BP	GO:0045926	negative regulation of growth	8	2.13E-02	1.75E-02	RGS2/CRYAB/FHL1/SLIT2/MT2A/WFDC1/SFRP2/MT1E
Downregulated DEGs	BP	GO:0072132	mesenchyme morphogenesis	4	2.18E-02	1.79E-02	ACTC1/ACTG2/FOXF1/ACTA2
Downregulated DEGs	BP	GO:0042391	regulation of membrane potential	10	2.19E-02	1.80E-02	CASQ2/CAV1/FHL1/P2RX1/DCN/JUN/BIN1/PLN/MEF2C/FLNA
Downregulated DEGs	BP	GO:0010927	cellular component assembly involved in morphogenesis	5	2.30E-02	1.89E-02	MYH11/ACTC1/CASQ2/TPM1/MEF2C
Downregulated DEGs	BP	GO:1904062	regulation of cation transmembrane transport	9	2.33E-02	1.91E-02	RGS2/CASQ2/CAV1/FHL1/GEM/BIN1/PLN/MEF2C/FLNA
Downregulated DEGs	BP	GO:0071241	cellular response to inorganic substance	7	2.50E-02	2.05E-02	CLIC4/MT2A/JUN/FOSB/MEF2C/SERPINF1/MT1E
Downregulated DEGs	BP	GO:0007229	integrin-mediated signaling pathway	5	2.55E-02	2.09E-02	FERMT2/ITGA5/COL3A1/FLNA/LIMS2
Downregulated DEGs	BP	GO:0072012	glomerulus vasculature development	3	2.55E-02	2.09E-02	EGR1/ACTA2/TCF21
Downregulated DEGs	BP	GO:0055123	digestive system development	6	2.64E-02	2.17E-02	FOXF1/RARRES2/TCF21/SFRP2/COL3A1/RBPMS2
Downregulated DEGs	BP	GO:0043271	negative regulation of ion transport	6	2.65E-02	2.17E-02	RGS2/CASQ2/CAV1/GEM/BIN1/PLN
Downregulated DEGs	BP	GO:0061337	cardiac conduction	6	2.65E-02	2.17E-02	CASQ2/CAV1/FXYD6/BIN1/PLN/FLNA
Downregulated DEGs	BP	GO:0007044	cell-substrate junction assembly	5	2.65E-02	2.17E-02	FERMT2/TNS1/ITGA5/FAM107A/ACTN1
Downregulated DEGs	BP	GO:0002027	regulation of heart rate	5	2.83E-02	2.32E-02	CASQ2/CAV1/BIN1/TPM1/PLN
Downregulated DEGs	BP	GO:0043266	regulation of potassium ion transport	5	2.83E-02	2.32E-02	CASQ2/CAV1/FHL1/BIN1/FLNA
Downregulated DEGs	BP	GO:0016049	cell growth	11	2.83E-02	2.32E-02	RGS2/CRYAB/FHL1/SLIT2/MAP1B/DPYSL2/FAM107A/WFDC1/SFRP2/FBLN5/ZEB2
Downregulated DEGs	BP	GO:0001558	regulation of cell growth	10	2.84E-02	2.33E-02	RGS2/CRYAB/FHL1/SLIT2/MAP1B/DPYSL2/FAM107A/WFDC1/SFRP2/FBLN5
Downregulated DEGs	BP	GO:0046677	response to antibiotic	9	2.84E-02	2.33E-02	RGS2/ACTC1/CRYAB/EGR1/AOC3/DUSP1/CYP1B1/JUN/MEF2C
Downregulated DEGs	BP	GO:0061437	renal system vasculature development	3	2.84E-02	2.33E-02	EGR1/ACTA2/TCF21
Downregulated DEGs	BP	GO:0061440	kidney vasculature development	3	2.84E-02	2.33E-02	EGR1/ACTA2/TCF21
Downregulated DEGs	BP	GO:0032272	negative regulation of protein polymerization	4	3.03E-02	2.49E-02	LMOD1/SLIT2/TMOD1/CLIP3
Downregulated DEGs	BP	GO:0006940	regulation of smooth muscle contraction	4	3.20E-02	2.62E-02	CNN1/RGS2/CAV1/P2RX1
Downregulated DEGs	BP	GO:0006027	glycosaminoglycan catabolic process	4	3.33E-02	2.73E-02	LUM/DCN/SDC2/FMOD
Downregulated DEGs	BP	GO:0032835	glomerulus development	4	3.33E-02	2.73E-02	EGR1/ACTA2/TCF21/MEF2C
Downregulated DEGs	BP	GO:0002688	regulation of leukocyte	5	3.41E-02	2.79E-02	SLIT2/DUSP1/GAS6/JAM3/RARRES2

			chemotaxis				
Downregulated DEGs	BP	GO:0007519	skeletal muscle tissue development	6	3.41E-02	2.79E-02	CAV1/EGR1/DCN/TCF21/MEF2C/ATF3
Downregulated DEGs	BP	GO:0090288	negative regulation of cellular response to growth factor stimulus	6	3.41E-02	2.79E-02	CAV1/SLIT2/DCN/TGFB1I1/SFRP2/RBPMS2
Downregulated DEGs	BP	GO:0006026	aminoglycan catabolic process	4	3.55E-02	2.91E-02	LUM/DCN/SDC2/FMOD
Downregulated DEGs	BP	GO:0071277	cellular response to calcium ion	4	3.55E-02	2.91E-02	CLIC4/JUN/FOSB/MEF2C
Downregulated DEGs	BP	GO:0006816	calcium ion transport	10	3.55E-02	2.91E-02	CASQ2/CAV1/P2RX1/MYLK/RAMP1/GAS6/GEM/BIN1/PLN/ANXA5
Downregulated DEGs	BP	GO:0032387	negative regulation of intracellular transport	4	3.92E-02	3.22E-02	CRYAB/FOXF1/MAP1B/PLN
Downregulated DEGs	BP	GO:0031032	actomyosin structure organization	6	4.01E-02	3.29E-02	CNN1/MYH11/ACTC1/CASQ2/TPM1/MEF2C
Downregulated DEGs	BP	GO:0050680	negative regulation of epithelial cell proliferation	6	4.01E-02	3.29E-02	CAV1/WFDC1/SFRP2/MEF2C/SERPINF1/LIMS2
Downregulated DEGs	BP	GO:0051926	negative regulation of calcium ion transport	4	4.03E-02	3.30E-02	CASQ2/GEM/BIN1/PLN
Downregulated DEGs	BP	GO:1901380	negative regulation of potassium ion transmembrane transport	3	4.08E-02	3.35E-02	CASQ2/CAV1/BIN1
Downregulated DEGs	BP	GO:0060538	skeletal muscle organ development	6	4.12E-02	3.38E-02	CAV1/EGR1/DCN/TCF21/MEF2C/ATF3
Downregulated DEGs	BP	GO:0009743	response to carbohydrate	7	4.12E-02	3.38E-02	EGR1/COL6A2/MAP1B/GAS6/COLEC12/SERPINF1/LGALS1
Downregulated DEGs	BP	GO:0030308	negative regulation of cell growth	6	4.29E-02	3.52E-02	RGS2/CRYAB/FHL1/SLIT2/WFDC1/SFRP2
Downregulated DEGs	BP	GO:0086003	cardiac muscle cell contraction	4	4.29E-02	3.52E-02	CAV1/BIN1/PLN/FLNA
Downregulated DEGs	BP	GO:0072001	renal system development	8	4.29E-02	3.52E-02	EGR1/SLIT2/FOXF1/DCN/ACTA2/TCF21/MEF2C/SERPINF1
Downregulated DEGs	BP	GO:0008217	regulation of blood pressure	6	4.47E-02	3.66E-02	PTGS1/P2RX1/GAS6/ACTA2/TPM1/CPA3
Downregulated DEGs	BP	GO:0043010	camera-type eye development	8	4.59E-02	3.76E-02	CRYAB/EFEMP1/CLIC4/TMOD1/CYP1B1/JUN/SERPINF1/ZEB2
Downregulated DEGs	BP	GO:0010959	regulation of metal ion transport	9	4.63E-02	3.80E-02	CASQ2/CAV1/FHL1/P2RX1/MYLK/GEM/BIN1/PLN/FLNA
Downregulated DEGs	BP	GO:0043407	negative regulation of MAP kinase activity	4	4.81E-02	3.94E-02	RGS2/CAV1/DUSP1/SFRP2
Downregulated DEGs	BP	GO:0098901	regulation of cardiac muscle cell action potential	3	4.85E-02	3.97E-02	CAV1/BIN1/FLNA
Downregulated DEGs	BP	GO:0090066	regulation of anatomical structure size	10	4.85E-02	3.97E-02	RGS2/LMOD1/CAV1/P2RX1/SLIT2/MAP1B/DPYSL2/TMOD1/ACTA2/BIN1
Downregulated DEGs	BP	GO:0032413	negative regulation of ion transmembrane transporter activity	4	4.93E-02	4.04E-02	CASQ2/CAV1/GEM/PLN
Downregulated DEGs	CC	GO:0062023	collagen-containing extracellular matrix	30	4.24E-19	3.21E-19	MFAP4/SRPX/SPARCL1/SPON1/EFEMP1/COL6A2/FGL2/LUM/MGP/AEBP1/CILP/DCN/MXRA7/RARRES2/COL6A3/TGFB1I1/TP

							SAB1/DPT/SOD3/CPA3/SFRP2/COL15A1/SERPINA3/FBLN5/COL3A1/SDC2/SERPINF1/ANXA5/FMOD/LGALS1
Downregulated DEGs	CC	GO:0031012	extracellular matrix	31	1.75E-18	1.32E-18	MFAP4/SRPX/SPARCL1/SPON1/EFEMP1/COL6A2/FGL2/LUM/MGP/AEBP1/CILP/DCN/MXRA7/RARRES2/CRISPLD2/COL6A3/TGFB1I1/TPSAB1/DPT/SOD3/CPA3/SFRP2/COL15A1/SERPINA3/FBLN5/COL3A1/SDC2/SERPINF1/ANXA5/FMOD/LGALS1
Downregulated DEGs	CC	GO:0043292	contractile fiber	22	3.76E-17	2.84E-17	SYNM/MYH11/LMOD1/ACTC1/PDLIM3/CRYAB/CASQ2/FERMT2/MYL9/DES/CALD1/TMOD1/FLNC/ACTA2/BIN1/TPM1/NEXN/AHNAK2/PALLD/TPM2/ACTN1/FLNA
Downregulated DEGs	CC	GO:0044449	contractile fiber part	21	8.48E-17	6.41E-17	SYNM/MYH11/LMOD1/ACTC1/PDLIM3/CRYAB/CASQ2/FERMT2/MYL9/DES/TMOD1/FLNC/ACTA2/BIN1/TPM1/NEXN/AHNAK2/PALLD/TPM2/ACTN1/FLNA
Downregulated DEGs	CC	GO:0030016	myofibril	20	2.06E-15	1.56E-15	SYNM/LMOD1/ACTC1/PDLIM3/CRYAB/CASQ2/FERMT2/MYL9/DES/CALD1/TMOD1/FLNC/BIN1/TPM1/NEXN/AHNAK2/PALLD/TPM2/ACTN1/FLNA
Downregulated DEGs	CC	GO:0030017	sarcomere	18	5.00E-14	3.78E-14	LMOD1/ACTC1/PDLIM3/CRYAB/CASQ2/FERMT2/MYL9/DES/TMOD1/FLNC/BIN1/TPM1/NEXN/AHNAK2/PALLD/TPM2/ACTN1/FLNA
Downregulated DEGs	CC	GO:0015629	actin cytoskeleton	23	7.25E-12	5.48E-12	MYH11/LMOD1/ACTC1/PDLIM3/ACTG2/CRYAB/FERMT2/MYL9/DPYSL3/CLIC4/MYLK/CALD1/TMOD1/FAM107A/SMTN/ACTA2/BIN1/TPM1/PALLD/TPM2/ACTN1/DDR2/FLNA
Downregulated DEGs	CC	GO:0031674	I band	14	7.25E-12	5.48E-12	ACTC1/PDLIM3/CRYAB/CASQ2/FERMT2/MYL9/DES/FLNC/BIN1/NEXN/AHNAK2/PALLD/ACTN1/FLNA
Downregulated DEGs	CC	GO:0030018	Z disc	12	7.53E-10	5.70E-10	PDLIM3/CRYAB/CASQ2/MYL9/DES/FLNC/BIN1/NEXN/AHNAK2/PALLD/ACTN1/FLNA
Downregulated DEGs	CC	GO:0005912	adherens junction	21	4.17E-09	3.16E-09	SYNM/CNN1/ACTC1/CAV1/FERMT2/FHL1/DES/TNS1/ITGA5/FAM107A/FLNC/TGFB1I1/MCAM/NEXN/PALLD/DIXDC1/ANXA5/ACTN1/DDR2/FLNA/LIMS2
Downregulated DEGs	CC	GO:0005925	focal adhesion	19	4.17E-09	3.16E-09	CNN1/ACTC1/CAV1/FERMT2/FHL1/TNS1/ITGA5/FAM107A/FLNC/TGFB1I1/MCAM/NEXN/PALLD/DIXDC1/ANXA5/ACTN1/DDR2/FLNA/LIMS2
Downregulated DEGs	CC	GO:0005924	cell-substrate adherens junction	19	4.17E-09	3.16E-09	CNN1/ACTC1/CAV1/FERMT2/FHL1/TNS1/ITGA5/FAM107A/FLNC/TGFB1I1/MCAM/NEXN/PALLD/DIXDC1/ANXA5/ACTN1/DDR2/FLNA/LIMS2
Downregulated DEGs	CC	GO:0030055	cell-substrate junction	19	4.75E-09	3.59E-09	CNN1/ACTC1/CAV1/FERMT2/FHL1/TNS1/

							ITGA5/FAM107A/FLNC/TGFB1I1/MCAM/NEXN/PALLD/DIXDC1/ANXA5/ACTN1/DDR2/FLNA/LIMS2
Downregulated DEGs	CC	GO:0032432	actin filament bundle	7	5.21E-06	3.94E-06	CRYAB/FERMT2/MYL9/MYLK/FAM107A/TPM1/ACTN1
Downregulated DEGs	CC	GO:0042641	actomyosin	7	1.18E-05	8.91E-06	ACTC1/FERMT2/MYL9/MYLK/FAM107A/TPM1/ACTN1
Downregulated DEGs	CC	GO:0005884	actin filament	7	2.18E-05	1.65E-05	LMOD1/ACTC1/DPYSL3/TMOD1/PALLD/ACTN1/FLNA
Downregulated DEGs	CC	GO:0001725	stress fiber	6	5.09E-05	3.85E-05	FERMT2/MYL9/MYLK/FAM107A/TPM1/ACTN1
Downregulated DEGs	CC	GO:0097517	contractile actin filament bundle	6	5.09E-05	3.85E-05	FERMT2/MYL9/MYLK/FAM107A/TPM1/ACTN1
Downregulated DEGs	CC	GO:0005581	collagen trimer	7	5.09E-05	3.85E-05	COL6A2/LUM/DCN/COLEC12/COL6A3/COL15A1/COL3A1
Downregulated DEGs	CC	GO:0005788	endoplasmic reticulum lumen	11	2.71E-04	2.05E-04	CASQ2/SPARCL1/SPON1/COL6A2/GAS6/COL6A3/COL15A1/COL3A1/SDC2/FSTL1/LGALS1
Downregulated DEGs	CC	GO:0044420	extracellular matrix component	5	3.10E-04	2.34E-04	MFAP4/LUM/COL15A1/FBLN5/COL3A1
Downregulated DEGs	CC	GO:0042383	sarcolemma	7	5.09E-04	3.85E-04	SYNM/COL6A2/DES/FLNC/COL6A3/BIN1/AHNAK2
Downregulated DEGs	CC	GO:0005796	Golgi lumen	6	1.30E-03	9.84E-04	LUM/DCN/GAS6/SOD3/SDC2/FMOD
Downregulated DEGs	CC	GO:0031093	platelet alpha granule lumen	5	1.51E-03	1.14E-03	CFD/GAS6/ISLR/SERPINA3/ACTN1
Downregulated DEGs	CC	GO:0031252	cell leading edge	11	1.65E-03	1.25E-03	ACTC1/ACTG2/FERMT2/DPYSL3/MYLK/ITGA5/FAM107A/ACTA2/TPM1/PALLD/ACTN1
Downregulated DEGs	CC	GO:0005865	striated muscle thin filament	3	1.97E-03	1.49E-03	TMOD1/TPM1/TPM2
Downregulated DEGs	CC	GO:0043034	costamere	3	2.73E-03	2.07E-03	SYNM/FLNC/AHNAK2
Downregulated DEGs	CC	GO:0036379	myofilament	3	3.11E-03	2.36E-03	TMOD1/TPM1/TPM2
Downregulated DEGs	CC	GO:0031091	platelet alpha granule	5	5.07E-03	3.84E-03	CFD/GAS6/ISLR/SERPINA3/ACTN1
Downregulated DEGs	CC	GO:0030027	lamellipodium	7	5.07E-03	3.84E-03	ACTC1/ACTG2/FERMT2/DPYSL3/MYLK/ACTA2/PALLD
Downregulated DEGs	CC	GO:0043202	lysosomal lumen	5	5.63E-03	4.26E-03	LUM/DCN/CTSK/SDC2/FMOD
Downregulated DEGs	CC	GO:0005775	vacuolar lumen	6	1.44E-02	1.09E-02	LUM/DCN/CTSK/SERPINA3/SDC2/FMOD
Downregulated DEGs	CC	GO:0005916	fascia adherens	2	1.71E-02	1.29E-02	DES/ACTN1
Downregulated DEGs	CC	GO:0016528	sarcoplasm	4	1.82E-02	1.37E-02	CASQ2/FLNC/PLN/MEF2C
Downregulated DEGs	CC	GO:0005583	fibrillar collagen trimer	2	1.90E-02	1.44E-02	LUM/COL3A1
Downregulated DEGs	CC	GO:0098643	banded collagen fibril	2	1.90E-02	1.44E-02	LUM/COL3A1
Downregulated DEGs	CC	GO:0043218	compact myelin	2	2.60E-02	1.97E-02	JAM3/PMP22
Downregulated DEGs	CC	GO:0072562	blood microparticle	5	3.24E-02	2.45E-02	ACTC1/ACTG2/C1S/SERPINA3/ANXA5
Downregulated DEGs	CC	GO:0098552	side of membrane	8	3.73E-02	2.82E-02	RGS2/FERMT2/P2RX1/RGS1/ITGA5/GEM/MCAM/ANXA5
Downregulated DEGs	CC	GO:0097440	apical dendrite	2	4.11E-02	3.11E-02	MAP1B/FLNA
Downregulated DEGs	CC	GO:0005859	muscle myosin complex	2	4.38E-02	3.32E-02	MYH11/MYL9
Downregulated DEGs	CC	GO:0098644	complex of collagen trimers	2	4.38E-02	3.32E-02	LUM/COL3A1
Downregulated DEGs	CC	GO:0031941	filamentous actin	2	4.65E-02	3.52E-02	DPYSL3/FLNA
Downregulated DEGs	CC	GO:0101031	chaperone complex	2	4.65E-02	3.52E-02	BAG2/HSPB8
Downregulated DEGs	CC	GO:0001726	ruffle	5	4.74E-02	3.59E-02	ITGA5/FAM107A/TPM1/PALLD/ACTN1

Downregulated DEGs	MF	GO:0005201	extracellular matrix structural constituent	17	3.17E-12	2.77E-12	MFAP4/SRPX/SPON1/EFEMP1/COL6A2/FGL2/LUM/MGP/AEBP1/CILP/DCN/COL6A3/DPT/COL15A1/FBLN5/COL3A1/FMOD
Downregulated DEGs	MF	GO:0003779	actin binding	20	1.85E-09	1.61E-09	CNN1/MYH11/TAGLN/LMOD1/FERMT2/MYLK/TNS1/CALD1/TMOD1/FAM107A/SMTN/FLNC/BIN1/TPM1/NEXN/PALLD/TPM2/DIXDC1/ACTN1/FLNA
Downregulated DEGs	MF	GO:0008307	structural constituent of muscle	8	3.64E-08	3.17E-08	SYNM/MYH11/PDLIM3/MYL9/SMTN/TPM1/NEXN/TPM2
Downregulated DEGs	MF	GO:0051015	actin filament binding	11	1.67E-06	1.46E-06	MYH11/TAGLN/FERMT2/TMOD1/FLNC/BIN1/TPM1/NEXN/TPM2/ACTN1/FLNA
Downregulated DEGs	MF	GO:0005516	calmodulin binding	9	4.65E-04	4.06E-04	PCP4/CNN1/RGS2/MYH11/RGS1/AEBP1/MYLK/CALD1/GEM
Downregulated DEGs	MF	GO:0005518	collagen binding	6	4.65E-04	4.06E-04	LUM/AEBP1/DCN/CTSK/PCOLCE2/DDR2
Downregulated DEGs	MF	GO:0046332	SMAD binding	5	1.04E-02	9.11E-03	JUN/TGFB1I1/COL3A1/FLNA/ZEB2
Downregulated DEGs	MF	GO:0005178	integrin binding	6	1.04E-02	9.11E-03	JAM3/ITGA5/SFRP2/FBLN5/COL3A1/ACTN1
Downregulated DEGs	MF	GO:0030020	extracellular matrix structural constituent conferring tensile strength	4	1.04E-02	9.11E-03	COL6A2/COL6A3/COL15A1/COL3A1
Downregulated DEGs	MF	GO:0030021	extracellular matrix structural constituent conferring compression resistance	3	1.76E-02	1.54E-02	LUM/DCN/FMOD
Downregulated DEGs	MF	GO:0061134	peptidase regulator activity	7	2.52E-02	2.20E-02	CAV1/GAS6/COL6A3/PCOLCE2/WFDC1/SFRP2/SERPINA3
Downregulated DEGs	MF	GO:0005539	glycosaminoglycan binding	7	2.85E-02	2.48E-02	SLIT2/DPYSL3/DCN/CRISPLD2/PCOLCE2/SOD3/FSTL1

Supplementary Table S4. KEGG pathway enrichment for the top three significant modules of the PPI network.

Module	Pathway	Count	P-value	Genes
Module1	Vascular smooth muscle contraction	4	1.60E-04	ACTG2, ACTA2, CALD1, MYLK
	Hypertrophic cardiomyopathy (HCM)	3	2.70E-03	ACTC1, TPM2, TPM1
	Cardiac muscle contraction	3	3.10E-03	ACTC1, TPM2, TPM1
	Dilated cardiomyopathy	3	3.10E-03	ACTC1, TPM2, TPM1
	Adrenergic signaling in cardiomyocytes	3	7.89E-03	ACTC1, TPM2, TPM1
Module2	Proteoglycans in cancer	3	2.10E-02	SDC1, CAV1, SDC2
	Malaria	2	5.70E-02	SDC1, SDC2
	ECM-receptor interaction	2	9.90E-02	SDC1, COL3A1
Module3	HTLV-I infection	3	7.10E-03	EGR1, JUN, CDC20
	Cell cycle	2	6.50E-02	CDC20, MCM4
	Ubiquitin mediated proteolysis	2	7.00E-02	CDC20, UBE2C

Supplementary Table S5. GO terms analysis for the hub genes. Through GO classification for the ten hub genes, eleven remarkably (adj. P-value ≤0.05) enriched GO terms were obtained, including biological process (BP), cellular component (CC) and molecular function (MF).

GO terms	GO ID	Description	Count	P-value	Gene ID
BP	GO:0007015	actin filament organization	2	0.024	ACTA2, ACTN1
BP	GO:0055002	striated muscle cell development	2	0.024	SDC1, ACTA2
BP	GO:0055001	muscle cell development	2	0.025	SDC1, ACTA2
BP	GO:0051146	striated muscle cell differentiation	2	0.039	SDC1, ACTA2
BP	GO:0042692	muscle cell differentiation	2	0.048	SDC1, ACTA2
CC	GO:0030016	myofibril	3	0.0016	ACTA2, ACTN1, TPM1
CC	GO:0043292	contractile fiber	3	0.0018	ACTA2, ACTN1, TPM1
CC	GO:0005884	actin filament	2	0.018	ACTA2, TPM1
CC	GO:0005856	cytoskeleton	4	0.032	ACTC1, ACTA2, ACTN1, TPM1
MF	GO:0008092	cytoskeletal protein binding	3	0.014	SDC1, ACTN1, TPM1
MF	GO:0019838	growth factor binding	2	0.043	COL3A1, IGFBP3

Note: BP, biological process; CC, cellular component; MF, molecular function

Supplementary Table S6. The genes correlated with *CDH1* expression based on data extracted from urinary tract cell samples via CCLE database.

Gene	Correlation coefficient	P-value
CDH1	1	0.00E+00
GRHL2	0.918	1.08E-10
MARVELD3	0.914	1.82E-10
ESRP1	0.91	3.04E-10
C1orf172	0.908	3.53E-10
EPHA1	0.902	7.46E-10
AP1M2	0.901	8.05E-10
LAD1	0.9	9.13E-10
TMEM30B	0.9	9.52E-10
ST14	0.899	1.02E-09
CDH3	0.891	2.46E-09
B3GNT3	0.883	5.27E-09
GALNT3	0.881	6.27E-09
CDC42BPG	0.877	8.90E-09
TSPAN13	0.868	1.91E-08
DAPP1	0.866	2.33E-08
SH2D3A	0.865	2.39E-08
CLDN4	0.858	4.18E-08
PRRG2	0.852	6.76E-08
ERBB3	0.851	6.96E-08
EPCAM	0.835	2.04E-07
LLGL2	0.831	2.65E-07
CGN	0.828	3.25E-07
RP11-532F12.5	0.825	3.93E-07
ITGB6	0.822	4.67E-07
ITGB4	0.821	4.95E-07
PLEKHG6	0.82	5.15E-07
JUP	0.82	5.39E-07
ITPKC	0.815	7.10E-07
C1orf106	0.813	8.01E-07
MYH14	0.812	8.23E-07
U47924.27	0.811	8.89E-07
LYPD3	0.809	1.00E-06
PRKCH	0.808	1.05E-06
GJB3	0.807	1.07E-06
RHOD	0.806	1.14E-06
GRTP1	0.806	1.14E-06
CCDC64B	0.806	1.18E-06
TMEM125	0.805	1.19E-06
PPL	0.805	1.19E-06

IRF6	0.804	1.32E-06
S100A14	0.803	1.34E-06
PIP4K2C	0.803	1.39E-06
C1orf116	0.801	1.53E-06
EHF	0.801	1.51E-06
ARHGAP27	0.801	1.54E-06
KIAA0040	0.8	1.62E-06
CLDN7	0.8	1.59E-06
GPR87	0.798	1.80E-06
ANO9	0.795	2.05E-06
RP3-523K23.2	0.79	2.67E-06
SLC44A3	0.789	2.76E-06
GRHL1	0.789	2.71E-06
CTSH	0.789	2.84E-06
EPS8L1	0.788	2.95E-06
PRSS16	0.787	3.06E-06
DDR1	0.787	3.10E-06
MPZL3	0.784	3.60E-06
PRSS22	0.782	3.91E-06
GRB7	0.781	4.07E-06
ADAP1	0.778	4.67E-06
MFSD11	0.777	4.89E-06
SYTL1	0.776	5.22E-06
ARHGEF35	0.776	5.13E-06
BSPRY	0.776	5.20E-06
HSH2D	0.776	5.21E-06
CCDC120	0.776	5.22E-06
TC2N	0.775	5.47E-06
AIM1L	0.773	5.88E-06
ARAP2	0.773	5.89E-06
ELMO3	0.773	5.93E-06
MYO5B	0.773	5.88E-06
VGLL1	0.773	5.84E-06
TSPAN15	0.772	6.14E-06
LPAR2	0.772	6.13E-06
SEMA4A	0.771	6.33E-06
PVRL4	0.771	6.58E-06
FAM83B	0.771	6.56E-06
ARHGEF16	0.769	6.92E-06
RP11-22C11.2	0.768	7.32E-06
IL4R	0.768	7.40E-06
MARVELD2	0.767	7.64E-06
TMEM184A	0.767	7.76E-06
PTPRU	0.764	8.96E-06

BAIAP2L1	0.764	8.98E-06
TMC6	0.763	9.24E-06
MAOA	0.762	9.73E-06
SOWAHB	0.761	9.86E-06
ESRP2	0.761	9.86E-06
TNFRSF21	0.758	1.12E-05
MIR205HG	0.757	1.19E-05
MPZL2	0.757	1.21E-05
ELF3	0.756	1.25E-05
KCNK1	0.756	1.26E-05
KCNMB4	0.755	1.30E-05
HCAR2	0.755	1.29E-05
ACOT11	0.752	1.43E-05
VWA1	0.751	1.55E-05
IGSF9	0.751	1.52E-05
SPINT2	0.751	1.53E-05
SLC37A1	0.75	1.58E-05
TTC9	0.749	1.62E-05
CAMSAP3	0.749	1.67E-05
TMC4	0.749	1.62E-05
INADL	0.748	1.74E-05
PTK2B	0.748	1.70E-05
CBLC	0.748	1.73E-05
IKZF2	0.746	1.88E-05
B3GALT4	0.746	1.86E-05
PRRG4	0.746	1.83E-05
HCAR3	0.746	1.86E-05
PTGES	0.744	2.05E-05
EPHB6	0.743	2.08E-05
PTAFR	0.742	2.17E-05
MDFI	0.742	2.22E-05
CST6	0.742	2.20E-05
DENND1C	0.741	2.29E-05
FAM110C	0.74	2.39E-05
MACC1	0.739	2.42E-05
MYO1D	0.738	2.55E-05
FGD3	0.736	2.79E-05
UPK2	0.736	2.72E-05
ST6GALNAC2	0.736	2.75E-05
RNF223	0.733	3.12E-05
C1QTNF6	0.733	3.07E-05
TACSTD2	0.732	3.19E-05
ABHD17C	0.732	3.19E-05
SERPINB5	0.731	3.33E-05

TP53TG1	0.725	4.12E-05
OR7E91P	0.724	4.28E-05
PRR15	0.724	4.25E-05
DTX4	0.723	4.44E-05
TJP3	0.723	4.42E-05
TNK1	0.722	4.64E-05
MISP	0.722	4.64E-05
GPR110	0.721	4.77E-05
PCDH1	0.72	4.97E-05
C6orf132	0.72	5.04E-05
FGFR2	0.72	4.96E-05
ARHGEF5	0.718	5.37E-05
MICALCL	0.718	5.33E-05
CD9	0.718	5.38E-05
STARD10	0.717	5.52E-05
FHDC1	0.715	5.99E-05
ITGA2	0.715	5.97E-05
LINC01057	0.713	6.36E-05
NIPAL1	0.711	6.71E-05
TMEM238	0.711	6.79E-05
TRIM29	0.71	7.09E-05
SLC29A3	0.709	7.14E-05
CD46	0.707	7.79E-05
PROM2	0.707	7.90E-05
GJB5	0.706	8.05E-05
LAMC2	0.706	7.95E-05
IL17RE	0.704	8.50E-05
CAPN1	0.704	8.60E-05
ADAM8	0.702	9.06E-05
MREG	0.701	9.62E-05
PKP3	0.701	9.45E-05
GJB2	0.701	9.57E-05
NEDD9	0.7	9.87E-05
IVL	0.698	1.04E-04
LSR	0.698	1.04E-04
CDC42EP5	0.698	1.05E-04
CSTA	0.697	1.08E-04
PIM1	0.697	1.10E-04
SCEL	0.697	1.07E-04
RBBP8NL	0.697	1.07E-04
SLC52A3	0.696	1.12E-04
MST1R	0.695	1.17E-04
RBM47	0.695	1.14E-04
EPPK1	0.695	1.17E-04

ANXA9	0.694	1.18E-04
FUCA1	0.693	1.23E-04
AGR2	0.693	1.22E-04
DSG2	0.692	1.26E-04
MAPK13	0.69	1.34E-04
CDKN1A	0.69	1.35E-04
RP11-274E7.2	0.689	1.39E-04
PERP	0.689	1.41E-04
ACSF2	0.689	1.40E-04
TMEM63A	0.688	1.45E-04
ALS2CL	0.686	1.55E-04
RP11-134G8.8	0.685	1.60E-04
ZNF385A	0.684	1.62E-04
PKP2	0.683	1.69E-04
KLC3	0.682	1.72E-04
PLXNB2	0.682	1.75E-04
UPK1B	0.681	1.79E-04
FUT3	0.681	1.77E-04
RASSF5	0.68	1.87E-04
TMPRSS13	0.68	1.87E-04
ARHGEF34P	0.679	1.88E-04
SLC39A9	0.679	1.87E-04
PHLDA3	0.677	2.01E-04
EXPH5	0.676	2.07E-04
ERP27	0.676	2.08E-04
RHOV	0.676	2.10E-04
FXYD3	0.676	2.10E-04
MYCL	0.675	2.13E-04
ARL14	0.675	2.12E-04
LNX1	0.675	2.15E-04
ANK3	0.675	2.11E-04
GRHL3	0.674	2.18E-04
TINAGL1	0.674	2.20E-04
TMBIM1	0.674	2.20E-04
RASEF	0.674	2.23E-04
LPAR5	0.674	2.19E-04
FAM84A	0.673	2.27E-04
SH3YL1	0.672	2.33E-04
DUSP11	0.672	2.36E-04
MAST4-AS1	0.671	2.41E-04
TEAD3	0.67	2.47E-04
WIBG	0.67	2.48E-04
LAMB3	0.669	2.56E-04
SLC15A2	0.668	2.65E-04

CARD14	0.668	2.60E-04
GALNT12	0.667	2.72E-04
SP6	0.666	2.81E-04
LAMA5	0.666	2.79E-04
FTLP14	0.665	2.85E-04
SLC6A11	0.664	2.97E-04
PWWP2B	0.664	2.95E-04
ELF1	0.663	3.03E-04
ATP2C2	0.662	3.10E-04
APOL6	0.662	3.17E-04
KRT19	0.661	3.21E-04
RP11-486A14.1	0.66	3.32E-04
S100A11	0.659	3.41E-04
BTG2	0.659	3.44E-04
NHLRC1	0.659	3.44E-04
TMBIM6	0.659	3.45E-04
ACOX1	0.659	3.43E-04
AC093162.5	0.658	3.52E-04
CEACAM1	0.658	3.46E-04
SORL1	0.657	3.60E-04
ANKEF1	0.657	3.58E-04
CYP2J2	0.656	3.75E-04
ENTPD3	0.656	3.71E-04
RP11-385J1.2	0.656	3.73E-04
NDFIP2	0.656	3.73E-04
SYT7	0.655	3.84E-04
PLEKHG3	0.655	3.83E-04
LIPG	0.655	3.79E-04
RP3-325F22.5	0.654	3.95E-04
SCAMP2	0.653	3.99E-04
ACSL3	0.652	4.16E-04
AC110619.1	0.652	4.14E-04
HNRNPA1P33	0.652	4.14E-04
TES	0.65	4.36E-04
TMED2	0.649	4.54E-04
RP11-44F14.2	0.649	4.43E-04
HOOK2	0.649	4.52E-04
GGCT	0.648	4.60E-04
FAM83A	0.648	4.55E-04
RAB17	0.647	4.70E-04
C3orf83	0.647	4.73E-04
SPPL2A	0.647	4.71E-04
USP43	0.647	4.78E-04
PTGS2	0.646	4.87E-04

FRK	0.646	4.81E-04
TMEM179B	0.646	4.90E-04
S100A9	0.645	5.03E-04
FAM25C	0.645	4.94E-04
SSH3	0.645	4.93E-04
ABCA7	0.645	5.03E-04
ANXA8L2	0.644	5.09E-04
P2RY2	0.644	5.16E-04
NYNRIN	0.644	5.13E-04
STYK1	0.643	5.31E-04
PRR15L	0.643	5.32E-04
SERINC2	0.641	5.48E-04
AKTIP	0.641	5.51E-04
PCTP	0.641	5.58E-04
TSPAN1	0.64	5.73E-04
AC110619.2	0.64	5.66E-04
HIP1R	0.64	5.66E-04
PCSK6	0.639	5.89E-04
TMEM134	0.638	6.04E-04
DIRC2	0.637	6.10E-04
SMIM22	0.637	6.22E-04
CTD-2267D19.3	0.637	6.22E-04
C2orf54	0.636	6.40E-04
TMEM40	0.636	6.29E-04
VIPR1	0.636	6.40E-04
UNC13D	0.635	6.44E-04
EXOC6B	0.634	6.70E-04
RP5-1159O4.1	0.633	6.77E-04
TMEM191A	0.633	6.92E-04
CHMP4C	0.632	7.08E-04
LAMA3	0.632	6.97E-04
ADIPOR1	0.631	7.19E-04
LEMD3	0.631	7.28E-04
UCA1	0.63	7.46E-04
IFNLR1	0.629	7.58E-04
ANXA8L1	0.629	7.49E-04
TDG	0.629	7.64E-04
RP11-473M20.5	0.629	7.60E-04
TRIM11	0.628	7.74E-04
RAB20	0.628	7.83E-04
RP11-299G20.2	0.628	7.81E-04
RP11-597D13.9	0.627	7.94E-04
COX19	0.627	8.04E-04
DUOXA1	0.627	7.95E-04

PAK6	0.626	8.12E-04
SPRR3	0.625	8.47E-04
SLC38A9	0.625	8.43E-04
CAMK2N1	0.624	8.56E-04
LRRC1	0.624	8.56E-04
TRAFD1	0.624	8.62E-04
KIAA0247	0.624	8.54E-04
SLC44A2	0.624	8.67E-04
SLC22A23	0.623	8.70E-04
TMED10	0.623	8.70E-04
FAM103A1	0.622	9.09E-04
NIPAL2	0.621	9.18E-04
COL17A1	0.621	9.31E-04
COG2	0.62	9.48E-04
ILDR1	0.62	9.51E-04
SERINC5	0.62	9.38E-04
PAG1	0.62	9.41E-04
TMBIM4	0.62	9.55E-04
GJB6	0.62	9.52E-04
FAM83F	0.62	9.51E-04
SHROOM2	0.62	9.42E-04
CTNND1	0.619	9.65E-04
AP001007.1	0.619	9.63E-04
GAREM	0.619	9.76E-04
TP63	0.618	9.96E-04
UPK3B	0.618	9.87E-04
RP11-363E7.4	0.618	1.00E-03
PIK3C2B	0.617	1.03E-03
LIPH	0.617	1.03E-03
KLF3	0.617	1.02E-03
SNCG	0.617	1.02E-03
TMPRSS4	0.617	1.02E-03
SPTB	0.617	1.02E-03
TNS4	0.617	1.02E-03
DSC2	0.616	1.04E-03
KIAA1522	0.615	1.08E-03
C2ORF15	0.615	1.06E-03
DOK7	0.615	1.06E-03
LYPD5	0.615	1.06E-03
DENND2D	0.614	1.10E-03
SPRR1B	0.614	1.10E-03
VAMP8	0.614	1.09E-03
SRD5A3	0.614	1.11E-03
CLDN23	0.614	1.09E-03

LMTK3	0.614	1.09E-03
NUDT4P1	0.613	1.12E-03
LGALS8	0.613	1.13E-03
S100P	0.613	1.12E-03
RP11-44F21.5	0.613	1.13E-03
C11orf52	0.612	1.15E-03
CTB-39G8.3	0.612	1.15E-03
LRP5	0.611	1.17E-03
TNRC6C-AS1	0.611	1.18E-03
AC009299.3	0.61	1.19E-03
HES1	0.61	1.21E-03
BPGM	0.61	1.22E-03
ALOX5	0.61	1.19E-03
ANKRD22	0.61	1.20E-03
KDM5B	0.609	1.24E-03
PFKFB2	0.609	1.25E-03
AMPD3	0.609	1.24E-03
LINC00920	0.608	1.25E-03
CHN2	0.607	1.30E-03
BLCAP	0.607	1.30E-03
TFAP2C	0.607	1.30E-03
TIGD2	0.606	1.33E-03
MYO6	0.606	1.32E-03
ZNF704	0.606	1.33E-03
SH3TC1	0.605	1.34E-03
HID1	0.605	1.34E-03
PDLIM1	0.604	1.37E-03
SLC22A20	0.604	1.40E-03
DAAM1	0.604	1.38E-03
TLE3	0.604	1.40E-03
RP1-313I6.12	0.603	1.41E-03
RP11-800A3.4	0.603	1.42E-03
HOMEZ	0.603	1.42E-03
WNT10A	0.602	1.46E-03
FGFR3	0.602	1.47E-03
ACER2	0.602	1.44E-03
CYB561	0.602	1.45E-03
KLK6	0.602	1.45E-03
RP11-73M7.1	0.601	1.48E-03
FAM84B	0.601	1.50E-03
LRG1	0.601	1.49E-03
SIK1	0.601	1.49E-03
DUOX1	0.6	1.54E-03
AC026806.2	0.6	1.53E-03

PMEL	0.599	1.56E-03
BATF	0.599	1.54E-03
SHROOM3	0.598	1.59E-03
SMIM5	0.598	1.60E-03
SPRR1A	0.596	1.66E-03
ZNF552	0.596	1.67E-03
DLX5	0.595	1.71E-03
RNF19B	0.594	1.75E-03
HOOK1	0.594	1.74E-03
NPTN	0.594	1.74E-03
FA2H	0.594	1.76E-03
KRT16	0.594	1.75E-03
PTK6	0.594	1.74E-03
TMEM159	0.593	1.79E-03
CREG1	0.592	1.82E-03
ASAH1	0.592	1.84E-03
AQP3	0.592	1.83E-03
KRT17	0.592	1.81E-03
TMEM54	0.591	1.86E-03
RP11-666A8.9	0.591	1.88E-03
MBP	0.591	1.88E-03
STAMBP	0.59	1.89E-03
KRCC1	0.59	1.90E-03
KIAA1217	0.59	1.92E-03
MANBA	0.589	1.96E-03
FAAH	0.588	1.98E-03
NCOA7	0.587	2.02E-03
RP11-65J3.1	0.587	2.03E-03
C15orf39	0.587	2.02E-03
ANKRA2	0.586	2.07E-03
ZNF165	0.586	2.08E-03
PVRL1	0.586	2.08E-03
NPC2	0.586	2.10E-03
EML2	0.586	2.10E-03
FAAH2	0.586	2.09E-03
AC007255.8	0.585	2.11E-03
MBNL3	0.585	2.14E-03
ZC3H12A	0.584	2.18E-03
DSP	0.584	2.17E-03
SLC11A2	0.584	2.19E-03
MYO5C	0.584	2.17E-03
GPR56	0.584	2.19E-03
TRAF4	0.584	2.19E-03
DTX2	0.583	2.21E-03

FAM214A	0.583	2.23E-03
AUP1	0.582	2.25E-03
MSX2	0.582	2.30E-03
RNF141	0.582	2.29E-03
NTN4	0.582	2.26E-03
FAM134C	0.582	2.26E-03
TINCR	0.582	2.25E-03
RP11-285F7.2	0.581	2.34E-03
TJP2	0.581	2.34E-03
SLC44A1	0.581	2.33E-03
ENDOD1	0.581	2.31E-03
ARHGAP32	0.581	2.30E-03
LRMP	0.581	2.32E-03
TM9SF2	0.581	2.30E-03
FURIN	0.581	2.31E-03
RAB11FIP1	0.58	2.37E-03
MB	0.58	2.37E-03
MFSD9	0.579	2.42E-03
HS3ST1	0.578	2.48E-03
LMBRD1	0.578	2.46E-03
C19orf82	0.578	2.47E-03
TNFAIP8	0.577	2.51E-03
SUN1	0.577	2.52E-03
FAM83H	0.577	2.53E-03
KRT13	0.577	2.53E-03
RNASEL	0.576	2.58E-03
MUC20	0.576	2.58E-03
IL20RA	0.576	2.61E-03
LRP11	0.576	2.58E-03
SPPL3	0.575	2.66E-03
EFNA1	0.574	2.67E-03
TMEM154	0.574	2.71E-03
ARID3B	0.574	2.69E-03
XG	0.574	2.70E-03
AC009478.1	0.573	2.78E-03
MAP3K9	0.573	2.73E-03
TRIM16	0.573	2.73E-03
AP001625.6	0.573	2.77E-03
DLX3	0.572	2.80E-03
PTGFRN	0.571	2.86E-03
ACOXL	0.571	2.85E-03
C4orf19	0.571	2.89E-03
RP11-7K24.3	0.571	2.86E-03
IFNGR1	0.571	2.90E-03

KLF5	0.57	2.96E-03
ZNF774	0.569	3.01E-03
ARHGAP5-AS1	0.568	3.07E-03
KRT15	0.568	3.04E-03
EVPL	0.568	3.06E-03
NPL	0.567	3.14E-03
C1orf74	0.567	3.09E-03
ALPP	0.567	3.11E-03
ICA1	0.567	3.13E-03
ARHGDIB	0.567	3.11E-03
PGAP3	0.567	3.09E-03
NOTCH3	0.566	3.18E-03
GDF15	0.566	3.17E-03
COL4A5	0.566	3.19E-03
WNT4	0.565	3.27E-03
SLC37A2	0.565	3.22E-03
CCDC64	0.565	3.24E-03
USP40	0.564	3.32E-03
ANKRD65	0.563	3.41E-03
RP3-406A7.7	0.563	3.36E-03
KCNK6	0.563	3.40E-03
OCLN	0.562	3.46E-03
TNFSF15	0.562	3.43E-03
RP6-65G23.3	0.562	3.45E-03
ABAT	0.562	3.48E-03
PEX13	0.561	3.54E-03
ACOT4	0.561	3.56E-03
SH3BP1	0.561	3.52E-03
FBLN1	0.561	3.49E-03
SLC1A3	0.56	3.63E-03
ATXN7L3B	0.56	3.63E-03
GDE1	0.56	3.58E-03
GCNT4	0.559	3.68E-03
RIPK3	0.559	3.65E-03
CYP2S1	0.559	3.68E-03
RNF13	0.558	3.74E-03
GATA3	0.558	3.77E-03
RIOK3	0.558	3.79E-03
RAP2B	0.557	3.83E-03
HCAR1	0.557	3.79E-03
IGSF3	0.556	3.89E-03
IL1RN	0.556	3.89E-03
NOTCH1	0.556	3.94E-03
HES2	0.555	3.99E-03

GOLT1A	0.555	4.00E-03
FAM89A	0.555	4.01E-03
SCNN1A	0.555	4.02E-03
ARIH1	0.555	4.01E-03
LGALS9	0.555	3.97E-03
MTND2P28	0.554	4.03E-03
DHRS2	0.554	4.06E-03
CTD-3128G10.6	0.554	4.06E-03
RIPK4	0.554	4.10E-03
TMEM165	0.553	4.11E-03
COX6A1P2	0.553	4.10E-03
EPHB4	0.553	4.17E-03
EPB41L4B	0.553	4.15E-03
SLC2A10	0.553	4.17E-03
COMT	0.553	4.15E-03
FAM171B	0.552	4.26E-03
PROSER2	0.552	4.22E-03
SH3TC2	0.551	4.32E-03
SVIL	0.551	4.31E-03
BLNK	0.551	4.30E-03
TSPAN2	0.55	4.41E-03
PLEKHG1	0.55	4.44E-03
C9orf169	0.55	4.40E-03
FAM3C2	0.55	4.38E-03
CTNNBIP1	0.549	4.46E-03
KRTCAP3	0.549	4.49E-03
P2RX4	0.549	4.51E-03
FERMT1	0.549	4.47E-03
CXADR	0.549	4.45E-03
CXCL16	0.548	4.60E-03
MT-TN	0.548	4.59E-03
SKIL	0.547	4.69E-03
B4GALT1	0.547	4.65E-03
AL603965.1	0.547	4.67E-03
MTUS1	0.546	4.78E-03
OAS1	0.546	4.76E-03
SDCBP2	0.546	4.72E-03
ZNRF2	0.545	4.88E-03
FAM3C	0.545	4.85E-03
MOB3B	0.545	4.87E-03
NEBL	0.545	4.82E-03
ALDH3B2	0.545	4.87E-03
RNF39	0.544	4.91E-03
NTF4	0.544	4.91E-03

TEN1	0.543	5.00E-03
POF1B	0.543	5.06E-03
RP11-902B17.1	0.542	5.12E-03
PLA2G4F	0.542	5.15E-03
DAPK2	0.542	5.16E-03
HSD11B2	0.542	5.12E-03
TMPRSS2	0.542	5.11E-03
PAQR7	0.541	5.27E-03
CCNYL1	0.541	5.23E-03
SPINK5	0.541	5.24E-03
HOXA1	0.541	5.28E-03
FAM83A-AS1	0.541	5.21E-03
CALML3-AS1	0.541	5.26E-03
TMEM51	0.54	5.30E-03
TMEM87A	0.54	5.30E-03
ARHGAP30	0.539	5.40E-03
SGK223	0.539	5.47E-03
TMEM45B	0.539	5.45E-03
ETHE1	0.539	5.43E-03
PPP1R13L	0.539	5.44E-03
ABLIM1	0.538	5.54E-03
HPGD	0.537	5.62E-03
SLC12A2	0.537	5.69E-03
SAMD9	0.537	5.65E-03
KLF4	0.537	5.65E-03
UNC93B1	0.537	5.68E-03
PEX11G	0.537	5.61E-03
MT-ND4	0.537	5.69E-03
RP11-480I12.5	0.536	5.72E-03
CHPF	0.536	5.75E-03
ZBED2	0.536	5.72E-03
CNGA1	0.536	5.77E-03
JAG2	0.536	5.80E-03
AIM1	0.535	5.85E-03
SYNE4	0.535	5.83E-03
TMEM27	0.535	5.85E-03
COL4A6	0.535	5.81E-03
TMEM41A	0.534	5.91E-03
RP11-488C13.5	0.534	6.02E-03
IL36RN	0.533	6.06E-03
BAZ2B	0.533	6.12E-03
TP53INP1	0.533	6.08E-03
IGF2	0.533	6.11E-03
MT-CO2	0.533	6.08E-03

CRABP2	0.532	6.22E-03
SUCO	0.532	6.24E-03
KRT6A	0.532	6.17E-03
ERGIC3	0.532	6.17E-03
WNT7A	0.531	6.26E-03
CROT	0.531	6.33E-03
DOCK8	0.531	6.35E-03
DDB2	0.531	6.26E-03
TNKS1BP1	0.531	6.27E-03
LGALS3	0.531	6.31E-03
SULT2B1	0.531	6.28E-03
FUT1	0.531	6.31E-03
RHOU	0.53	6.42E-03
KHNYN	0.53	6.41E-03
CLDN9	0.53	6.41E-03
MT-ND1	0.53	6.43E-03
PLSCR1	0.529	6.51E-03
EPS8L2	0.529	6.54E-03
C16orf74	0.529	6.54E-03
RIPPLY3	0.529	6.55E-03
METTL7A	0.528	6.64E-03
TMOD3	0.528	6.70E-03
SETBP1	0.528	6.70E-03
LAMP3	0.527	6.84E-03
ENTPD2	0.527	6.80E-03
ZNF641	0.527	6.80E-03
DGKA	0.527	6.82E-03
GOLGA5	0.527	6.74E-03
MT-CO1	0.527	6.84E-03
MT-CO3	0.527	6.84E-03
MAST4	0.526	6.97E-03
CARD11	0.526	6.89E-03
AC068580.7	0.526	6.88E-03
ATP2A2	0.526	6.94E-03
AP1G2	0.526	6.93E-03
ITCH	0.526	6.90E-03
MT-ND3	0.526	6.86E-03
TMEM133	0.525	7.06E-03
RP11-705C15.2	0.525	7.03E-03
CD24P4	0.525	7.08E-03
SH3BGRL2	0.524	7.22E-03
FGD4	0.524	7.21E-03
STRA6	0.524	7.12E-03
LINC00511	0.524	7.17E-03

BIK	0.524	7.21E-03
DHRS3	0.523	7.34E-03
TMEM51-AS1	0.523	7.32E-03
LY75	0.523	7.31E-03
N4BP3	0.523	7.27E-03
KCNK5	0.523	7.34E-03
KB-1562D12.1	0.523	7.37E-03
ERMP1	0.523	7.28E-03
FAM107B	0.523	7.27E-03
TMPRSS11E	0.522	7.49E-03
LRCH4	0.522	7.51E-03
GPRC5A	0.522	7.43E-03
KRT18	0.522	7.45E-03
ECH1	0.522	7.42E-03
SPTLC3	0.522	7.48E-03
OSBPL2	0.522	7.48E-03
SLITRK6	0.521	7.60E-03
SLC39A2	0.521	7.53E-03
ZBTB42	0.52	7.74E-03
FRRS1	0.518	7.98E-03
TRPV6	0.518	8.02E-03
CTD-2003C8.2	0.518	8.01E-03
WNT7B	0.518	8.01E-03
SMPDL3B	0.517	8.19E-03
SLAMF7	0.517	8.09E-03
IL1A	0.517	8.18E-03
MESDC2	0.517	8.15E-03
NEK8	0.517	8.08E-03
WNK2	0.516	8.33E-03
NUBPL	0.516	8.27E-03
DLG3	0.516	8.30E-03
PIGV	0.515	8.37E-03
CLUHP3	0.515	8.36E-03
CACNG4	0.515	8.43E-03
LIMK2	0.515	8.47E-03
MT-TC	0.515	8.48E-03
CASP10	0.514	8.56E-03
RP5-1148A21.3	0.514	8.64E-03
TAX1BP1	0.514	8.56E-03
PLCH2	0.513	8.80E-03
RNF103	0.513	8.74E-03
CAB39	0.513	8.75E-03
ALDH1A3	0.513	8.77E-03
SRPX2	0.513	8.77E-03

RP5-973M2.2	0.512	8.93E-03
TMCC1	0.512	8.83E-03
RNASE1	0.512	8.91E-03
SOX15	0.512	8.95E-03
MT-TS1	0.512	8.92E-03
CPT2	0.511	9.10E-03
EPAS1	0.511	9.09E-03
B4GALT4	0.511	8.98E-03
ZSCAN12P1	0.511	9.07E-03
SLC17A5	0.511	8.97E-03
PLEKHH1	0.511	9.08E-03
RFWD2	0.51	9.27E-03
ABCA12	0.51	9.27E-03
ZNF860	0.51	9.21E-03
SYT8	0.51	9.15E-03
STON2	0.51	9.22E-03
HELZ2	0.51	9.26E-03
RABGAP1L	0.509	9.38E-03
SDC1	0.509	9.43E-03
SLC10A7	0.509	9.32E-03
TMEM144	0.509	9.30E-03
NIPAL4	0.509	9.38E-03
RCE1	0.509	9.40E-03
CAPN13	0.508	9.50E-03
ZNF750	0.508	9.45E-03
DENND1B	0.507	9.76E-03
OLR1	0.507	9.77E-03
RAB27B	0.507	9.73E-03
RP11-488P3.1	0.506	9.88E-03
TET3	0.506	9.80E-03
CREG2	0.506	9.83E-03
PLEKHF2	0.506	9.85E-03
TNNI2	0.506	9.87E-03
RP11-734K2.4	0.506	9.83E-03
AC012318.3	0.506	9.89E-03
SRC	0.505	9.96E-03
ANKRD33B	-0.506	9.82E-03
GAPDHP63	-0.506	9.82E-03
DIP2C	-0.506	9.82E-03
KIF18B	-0.506	9.90E-03
NDUFA11	-0.506	9.90E-03
C1orf86	-0.507	9.66E-03
MDN1	-0.507	9.71E-03
RP1-239B22.5	-0.507	9.76E-03

PLK1	-0.507	9.69E-03
AIFM1	-0.507	9.70E-03
LRRC58	-0.508	9.45E-03
DDX21	-0.508	9.54E-03
KATNB1	-0.508	9.45E-03
ELAC2	-0.508	9.49E-03
SRSF1	-0.508	9.48E-03
DOCK4	-0.509	9.33E-03
RP11-1002K11.1	-0.509	9.30E-03
NEK6	-0.509	9.28E-03
RP11-452K12.7	-0.509	9.31E-03
NRIP3	-0.509	9.41E-03
ARHGEF25	-0.509	9.35E-03
PITPNM3	-0.509	9.41E-03
PRR3	-0.51	9.21E-03
RP11-706J10.1	-0.51	9.18E-03
SEC31B	-0.51	9.27E-03
C11orf95	-0.51	9.27E-03
CXXC1	-0.51	9.13E-03
NREP	-0.511	8.98E-03
RUSC2	-0.511	9.07E-03
PITRM1	-0.511	9.05E-03
HSPA14	-0.511	9.10E-03
RHOG	-0.511	9.12E-03
JAM3	-0.511	8.99E-03
GDF11	-0.511	9.06E-03
ACTR3	-0.512	8.84E-03
ITPR1	-0.512	8.93E-03
RP11-1415C14.3	-0.512	8.82E-03
NAV3	-0.512	8.82E-03
ASMTL	-0.512	8.94E-03
THAP3	-0.513	8.71E-03
NPHP1	-0.513	8.73E-03
ATXN7	-0.513	8.79E-03
FBXW11	-0.513	8.72E-03
RBM17	-0.513	8.71E-03
AKIP1	-0.513	8.76E-03
RP11-521B24.3	-0.513	8.70E-03
UBTF	-0.513	8.79E-03
SEC14L1	-0.513	8.76E-03
UBE2D2	-0.514	8.63E-03
NRG2	-0.514	8.56E-03
CCDC86	-0.514	8.59E-03
HOXC6	-0.514	8.60E-03

C12orf75	-0.514	8.55E-03
BUB1B	-0.514	8.58E-03
TNNT1	-0.514	8.61E-03
WDR4	-0.514	8.63E-03
GNAZ	-0.514	8.58E-03
DUSP18	-0.514	8.59E-03
SMTN	-0.514	8.60E-03
YWHAH	-0.514	8.57E-03
CRMP1	-0.515	8.40E-03
IL6ST	-0.515	8.39E-03
CDC23	-0.515	8.47E-03
BTN2A1	-0.515	8.39E-03
TUBA1B	-0.515	8.48E-03
SPATC1L	-0.515	8.50E-03
SELK	-0.516	8.28E-03
VAMP1	-0.516	8.27E-03
POLDIP2	-0.516	8.22E-03
ACLY	-0.516	8.35E-03
CEP250	-0.516	8.25E-03
ATP11C	-0.516	8.33E-03
S1PR1	-0.517	8.16E-03
RP11-479G22.8	-0.517	8.15E-03
CCDC102A	-0.517	8.07E-03
GEMIN4	-0.517	8.20E-03
PMM1	-0.517	8.08E-03
CHRDL1	-0.517	8.07E-03
ATP6V1B2	-0.518	8.06E-03
SLC25A51	-0.518	8.06E-03
SUPV3L1	-0.518	7.96E-03
GLUD1P3	-0.518	7.99E-03
NOC3L	-0.518	8.02E-03
LDHAP5	-0.518	8.05E-03
C2CD2L	-0.518	7.92E-03
TULP3	-0.518	7.94E-03
TFE3	-0.518	7.97E-03
WIPF1	-0.519	7.89E-03
PRDM5	-0.519	7.86E-03
DCTN4	-0.519	7.86E-03
HSF2	-0.519	7.91E-03
RP11-398C13.6	-0.519	7.80E-03
ZNF37A	-0.519	7.91E-03
MLST8	-0.519	7.81E-03
METTL22	-0.519	7.86E-03
TNFAIP8L1	-0.519	7.88E-03

AC079922.3	-0.52	7.75E-03
RIOK2	-0.52	7.72E-03
MRPL23	-0.52	7.68E-03
SLC39A13	-0.52	7.66E-03
COL6A2	-0.52	7.74E-03
AC098614.2	-0.521	7.59E-03
CMC1	-0.521	7.52E-03
ARL6	-0.521	7.54E-03
ACO1	-0.521	7.63E-03
KPNA3	-0.521	7.64E-03
CTD-2020K17.1	-0.521	7.52E-03
CERK	-0.521	7.57E-03
PMS1	-0.522	7.42E-03
TRNT1	-0.522	7.46E-03
CAMK1	-0.522	7.49E-03
PCOLCE2	-0.522	7.41E-03
MURC	-0.522	7.43E-03
ASRGL1	-0.522	7.43E-03
AAAS	-0.522	7.39E-03
EIF5A	-0.522	7.44E-03
TUBB6	-0.522	7.42E-03
TBCB	-0.522	7.42E-03
DPF1	-0.522	7.43E-03
SMG9	-0.522	7.40E-03
SYP	-0.522	7.50E-03
HSPD1	-0.523	7.30E-03
CRTAP	-0.523	7.34E-03
LRRC14	-0.523	7.26E-03
LINC00958	-0.523	7.32E-03
P4HA3	-0.523	7.30E-03
GAPDH	-0.523	7.25E-03
PTPN14	-0.524	7.17E-03
HPCAL1	-0.524	7.23E-03
RP11-242F4.2	-0.524	7.24E-03
PCGF5	-0.524	7.17E-03
CCDC34	-0.524	7.22E-03
PPME1	-0.524	7.12E-03
MRPL37	-0.525	7.06E-03
MSX1	-0.525	7.09E-03
KIAA1430	-0.525	7.10E-03
TTC33	-0.525	7.01E-03
ELL2	-0.525	7.00E-03
GAPDHP72	-0.525	7.01E-03
SNX18P7	-0.525	6.99E-03

RP11-462L8.1	-0.525	6.98E-03
STK32C	-0.525	7.00E-03
C12orf10	-0.525	7.11E-03
SNORD10	-0.525	7.01E-03
SNORA71B	-0.525	7.00E-03
HMGB2	-0.526	6.94E-03
U91328.19	-0.526	6.94E-03
NRBP2	-0.526	6.94E-03
RAP2A	-0.526	6.92E-03
STAU2	-0.527	6.81E-03
KHDRBS3	-0.527	6.80E-03
RP11-166D19.1	-0.527	6.76E-03
CYB5D1	-0.527	6.73E-03
DBF4B	-0.527	6.79E-03
NKIRAS1	-0.528	6.71E-03
NEK1	-0.528	6.71E-03
SOCS3	-0.528	6.69E-03
KCNAB2	-0.529	6.50E-03
PARK7	-0.529	6.59E-03
KIF1B	-0.529	6.58E-03
C2orf69	-0.529	6.50E-03
TRAK2	-0.529	6.61E-03
RFTN1	-0.529	6.50E-03
KCNQ5	-0.529	6.59E-03
ECD	-0.529	6.57E-03
AAMDC	-0.529	6.50E-03
KIAA0753	-0.529	6.56E-03
C19orf70	-0.529	6.55E-03
PPM1F	-0.529	6.60E-03
RP4-555D20.4	-0.53	6.45E-03
EXOSC9	-0.53	6.38E-03
NQO2	-0.53	6.41E-03
SLC43A1	-0.53	6.47E-03
COA4	-0.53	6.37E-03
LATS2	-0.53	6.37E-03
GPC6	-0.53	6.39E-03
ZNF580	-0.53	6.43E-03
RP11-386I14.4	-0.531	6.34E-03
AIDA	-0.531	6.26E-03
RPP14	-0.531	6.33E-03
FOXC2	-0.531	6.28E-03
FMNL1	-0.531	6.28E-03
CTD-2521M24.6	-0.531	6.29E-03
C20orf27	-0.531	6.27E-03

STRADB	-0.532	6.14E-03
NRG1	-0.532	6.16E-03
CNTF	-0.532	6.24E-03
SENP3	-0.532	6.22E-03
C17orf51	-0.532	6.22E-03
CCDC74A	-0.533	6.12E-03
ABHD14B	-0.533	6.09E-03
GTF2H2C	-0.533	6.09E-03
G3BP1	-0.533	6.13E-03
RP11-613M10.6	-0.533	6.07E-03
ALDH1B1	-0.533	6.04E-03
TLE4	-0.533	6.07E-03
SLC25A15	-0.533	6.11E-03
PURA	-0.534	5.97E-03
RBM24	-0.534	5.98E-03
NTMT1	-0.534	5.92E-03
ANKRD26	-0.534	5.99E-03
MYL6B	-0.534	5.98E-03
RP11-849F2.9	-0.534	6.01E-03
RPL14	-0.535	5.89E-03
WAPAL	-0.535	5.83E-03
PRR11	-0.535	5.83E-03
RP11-674N23.1	-0.535	5.87E-03
KIRREL	-0.536	5.77E-03
CHST10	-0.536	5.79E-03
POMGNT2	-0.536	5.76E-03
ARL13B	-0.536	5.80E-03
TUBB	-0.536	5.78E-03
HBS1L	-0.536	5.79E-03
C9orf37	-0.536	5.76E-03
DCAF4	-0.536	5.73E-03
HOXB2	-0.536	5.76E-03
CTXN1	-0.536	5.75E-03
POLR2F	-0.536	5.73E-03
TMSB15A	-0.536	5.79E-03
GNL3	-0.537	5.66E-03
FAHD1	-0.537	5.65E-03
EIF4ENIF1	-0.537	5.65E-03
LRRFIP2	-0.538	5.49E-03
OGFRL1	-0.538	5.52E-03
PRUNE2	-0.538	5.57E-03
MCM10	-0.538	5.50E-03
ANKRD54	-0.538	5.54E-03
IRAK1	-0.538	5.49E-03

ITGB1BP1	-0.539	5.45E-03
IP6K1	-0.539	5.41E-03
LINC00472	-0.539	5.45E-03
HSPA8P8	-0.539	5.46E-03
HCFC2	-0.539	5.48E-03
LSM7	-0.539	5.48E-03
TRMT1	-0.539	5.43E-03
STX10	-0.539	5.48E-03
PM20D2	-0.54	5.30E-03
DOHH	-0.54	5.32E-03
FANCB	-0.54	5.34E-03
BAP1	-0.541	5.23E-03
ABHD10	-0.541	5.21E-03
PPP1R18	-0.541	5.18E-03
C2CD5	-0.541	5.27E-03
DHX33	-0.541	5.20E-03
KXD1	-0.541	5.27E-03
PLCG1	-0.541	5.27E-03
MTFR1L	-0.542	5.13E-03
MRPS25	-0.542	5.08E-03
HLTF	-0.542	5.10E-03
MTHFD2L	-0.542	5.12E-03
TRIM52-AS1	-0.542	5.08E-03
PSIP1	-0.542	5.11E-03
BCCIP	-0.542	5.16E-03
PAFAH1B1	-0.542	5.11E-03
SPHK2	-0.542	5.10E-03
NANP	-0.542	5.16E-03
APOO	-0.542	5.09E-03
LAGE3	-0.542	5.09E-03
TRABD2A	-0.543	5.04E-03
RP11-85K15.2	-0.543	5.04E-03
IFT172	-0.544	4.92E-03
ASNSD1	-0.544	4.95E-03
C3orf14	-0.544	4.97E-03
FBL	-0.544	4.99E-03
PES1	-0.544	4.93E-03
PTTG1	-0.545	4.85E-03
ZC3HC1	-0.545	4.87E-03
TIMM10	-0.545	4.81E-03
SLC26A10	-0.545	4.84E-03
APBA2	-0.545	4.80E-03
C17orf49	-0.545	4.83E-03
CEP89	-0.545	4.83E-03

LRRC6	-0.546	4.71E-03
NUP88	-0.546	4.74E-03
MED31	-0.546	4.78E-03
KLF16	-0.546	4.80E-03
CDC25B	-0.546	4.72E-03
ANKRD36	-0.547	4.62E-03
RP11-109N23.6	-0.547	4.67E-03
POLR3F	-0.547	4.67E-03
CCDC117	-0.547	4.67E-03
CYR61	-0.548	4.58E-03
PARD3B	-0.548	4.59E-03
WDR82	-0.548	4.59E-03
FAM86DP	-0.548	4.58E-03
RWDD4	-0.548	4.54E-03
PFKP	-0.548	4.61E-03
PAX6	-0.548	4.56E-03
PKD1P6	-0.548	4.59E-03
SOGA2	-0.548	4.58E-03
AXL	-0.548	4.54E-03
DRG1	-0.548	4.53E-03
DNAJC8	-0.549	4.50E-03
PLEKHO1	-0.549	4.49E-03
AC093724.2	-0.549	4.51E-03
TTC1	-0.549	4.45E-03
AHI1	-0.549	4.52E-03
PDZD8	-0.549	4.50E-03
SCN8A	-0.549	4.52E-03
COL18A1	-0.549	4.50E-03
SEPHS1	-0.55	4.35E-03
MASTL	-0.55	4.38E-03
RUFY2	-0.55	4.38E-03
NUP98	-0.55	4.40E-03
USP35	-0.55	4.44E-03
AC027307.3	-0.55	4.39E-03
B9D2	-0.55	4.37E-03
LDOC1L	-0.55	4.38E-03
EFHC1	-0.551	4.33E-03
JAZF1	-0.551	4.35E-03
FAM155A	-0.551	4.31E-03
STK4	-0.551	4.32E-03
RP6-109B7.3	-0.551	4.29E-03
ZNF674-AS1	-0.551	4.35E-03
LSM10	-0.552	4.20E-03
SGOL1	-0.552	4.26E-03

RPSA	-0.552	4.25E-03
PPID	-0.552	4.23E-03
SPATA24	-0.552	4.20E-03
NKX2-5	-0.552	4.24E-03
DPCD	-0.552	4.19E-03
CYB5RL	-0.553	4.15E-03
HOXD4	-0.553	4.14E-03
CEP57L1	-0.553	4.13E-03
GINS4	-0.553	4.17E-03
POU6F1	-0.553	4.15E-03
PPP1R9B	-0.553	4.18E-03
ELAVL1	-0.553	4.16E-03
TIMM23	-0.554	4.03E-03
RRP12	-0.554	4.06E-03
SMC3	-0.554	4.07E-03
L3HYPDH	-0.554	4.10E-03
PARVB	-0.554	4.05E-03
ACTR8	-0.555	3.95E-03
FJX1	-0.555	3.96E-03
CRLF3	-0.555	3.96E-03
GTSE1	-0.555	3.99E-03
FAM161A	-0.556	3.88E-03
BIN1	-0.556	3.91E-03
POC1A	-0.556	3.89E-03
EVC	-0.556	3.89E-03
DPY19L2P1	-0.556	3.91E-03
AQP11	-0.556	3.93E-03
IL13RA2	-0.556	3.93E-03
SRM	-0.557	3.81E-03
RUVBL1	-0.557	3.85E-03
RAB23	-0.557	3.85E-03
DDX50	-0.557	3.86E-03
RP11-297D21.4	-0.557	3.83E-03
LL22NC03-2H8.5	-0.557	3.85E-03
ZC4H2	-0.557	3.81E-03
DLX1	-0.558	3.72E-03
TMEM158	-0.558	3.79E-03
CTD-2010I16.1	-0.558	3.77E-03
IPO5	-0.558	3.72E-03
NETO2	-0.558	3.78E-03
RBMX2	-0.558	3.71E-03
RNF130	-0.559	3.68E-03
QSER1	-0.559	3.71E-03
RP11-22P6.3	-0.559	3.68E-03

MAP2K2	-0.559	3.69E-03
AC108488.3	-0.56	3.58E-03
PSMD6	-0.56	3.58E-03
DBN1	-0.56	3.59E-03
C7orf55	-0.56	3.58E-03
RRAS2	-0.56	3.61E-03
RNASEH2A	-0.56	3.60E-03
TWF2	-0.561	3.56E-03
ETV5	-0.561	3.53E-03
KIF20A	-0.561	3.52E-03
OGDHL	-0.561	3.51E-03
NEU3	-0.561	3.54E-03
ATF2	-0.562	3.44E-03
RASSF1	-0.562	3.48E-03
MPP6	-0.562	3.48E-03
GKAP1	-0.562	3.49E-03
RIMKLB	-0.562	3.47E-03
NT5DC3	-0.562	3.43E-03
PKN1	-0.562	3.45E-03
LAS1L	-0.562	3.42E-03
ASUN	-0.563	3.41E-03
KANK2	-0.563	3.37E-03
SLC25A53	-0.563	3.36E-03
DHX30	-0.564	3.34E-03
BBX	-0.564	3.33E-03
SNX25	-0.564	3.30E-03
CWF19L1	-0.564	3.35E-03
CLMP	-0.564	3.35E-03
EID1	-0.564	3.29E-03
CUL4B	-0.564	3.32E-03
AK2	-0.565	3.26E-03
C4orf27	-0.565	3.27E-03
POLR3A	-0.565	3.23E-03
SMIM4	-0.566	3.21E-03
BTN2A3P	-0.566	3.18E-03
CUTC	-0.566	3.15E-03
UBE2S	-0.566	3.20E-03
SLCO4A1	-0.566	3.21E-03
PHF6	-0.566	3.22E-03
KPNA1	-0.567	3.11E-03
AKAP2	-0.567	3.10E-03
SNX21	-0.567	3.12E-03
GPRIN1	-0.568	3.04E-03
C11orf74	-0.568	3.03E-03

RNFT2	-0.568	3.07E-03
MPP3	-0.568	3.04E-03
CCDC106	-0.568	3.07E-03
FAM208A	-0.569	2.98E-03
RP5-1050D4.5	-0.569	3.01E-03
TCF3	-0.569	3.02E-03
IKBKG	-0.569	3.01E-03
LINC00342	-0.57	2.96E-03
LPAR1	-0.57	2.91E-03
RSAD1	-0.57	2.92E-03
GADD45GIP1	-0.57	2.95E-03
WDR62	-0.57	2.91E-03
FBXO17	-0.57	2.90E-03
RHOA	-0.571	2.88E-03
TTC37	-0.571	2.89E-03
REEP2	-0.571	2.88E-03
CUTA	-0.571	2.85E-03
ARMC4	-0.571	2.85E-03
PRDX3	-0.571	2.89E-03
TEX30	-0.571	2.85E-03
GAS6-AS2	-0.571	2.89E-03
SOGA1	-0.571	2.88E-03
KIAA0754	-0.572	2.80E-03
ADAM12	-0.572	2.79E-03
ADARB1	-0.572	2.84E-03
TMA16	-0.573	2.75E-03
RGMB-AS1	-0.573	2.73E-03
INPP5E	-0.573	2.75E-03
ARL3	-0.573	2.74E-03
UHRF1BP1L	-0.573	2.78E-03
RABEP1	-0.573	2.76E-03
NLGN2	-0.573	2.73E-03
POLR1E	-0.574	2.70E-03
RABEPK	-0.574	2.72E-03
RASSF8-AS1	-0.574	2.69E-03
HOXC8	-0.574	2.67E-03
SUGP2	-0.574	2.69E-03
GAS2L1	-0.574	2.69E-03
NLN	-0.575	2.66E-03
SF3B5	-0.575	2.61E-03
PDE1C	-0.575	2.67E-03
RP11-3J10.4	-0.575	2.63E-03
NCS1	-0.575	2.64E-03
SGTA	-0.575	2.64E-03

DNAJC9	-0.576	2.59E-03
RP11-818F20.5	-0.576	2.57E-03
TYMS	-0.576	2.56E-03
ZMAT5	-0.576	2.57E-03
PRAF2	-0.576	2.59E-03
CDKN2AIPNL	-0.577	2.54E-03
WASF1	-0.577	2.53E-03
EXOSC2	-0.577	2.55E-03
ESD	-0.577	2.54E-03
ABI3BP	-0.578	2.47E-03
RAP1GDS1	-0.578	2.46E-03
CCDC107	-0.578	2.50E-03
DPP9	-0.578	2.45E-03
ZNF330	-0.579	2.41E-03
CNTLN	-0.579	2.45E-03
ATP8B2	-0.58	2.38E-03
ANKRD36C	-0.58	2.37E-03
LZTFL1	-0.58	2.37E-03
SIRT1	-0.58	2.35E-03
CBL	-0.58	2.35E-03
DYNC1H1	-0.58	2.40E-03
CYLD	-0.58	2.38E-03
ALKBH7	-0.58	2.39E-03
ENY2	-0.581	2.31E-03
PMPCA	-0.581	2.31E-03
PRPF8	-0.581	2.33E-03
C18orf54	-0.581	2.32E-03
CCDC124	-0.581	2.34E-03
C4orf46	-0.582	2.25E-03
IK	-0.582	2.29E-03
RNH1	-0.582	2.29E-03
AP2A2	-0.582	2.28E-03
SEC23A	-0.582	2.29E-03
AC005789.11	-0.582	2.29E-03
FGFRL1	-0.583	2.22E-03
WWC2	-0.583	2.24E-03
ARL14EP	-0.583	2.23E-03
CRY1	-0.583	2.24E-03
PFN1	-0.583	2.24E-03
SNX7	-0.584	2.19E-03
AGPAT4	-0.584	2.15E-03
LEPREL2	-0.584	2.17E-03
TPGS1	-0.584	2.17E-03
DAPK3	-0.584	2.17E-03

RUVBL2	-0.584	2.17E-03
TRIM36	-0.585	2.15E-03
DUSP1	-0.585	2.15E-03
UTRN	-0.585	2.12E-03
KAT6B	-0.585	2.14E-03
CAMTA1	-0.586	2.09E-03
NUF2	-0.586	2.10E-03
SHQ1	-0.586	2.09E-03
LOX	-0.586	2.07E-03
PTDSS2	-0.586	2.06E-03
SCML2	-0.586	2.09E-03
OCRL	-0.586	2.10E-03
NR2C2	-0.587	2.05E-03
PRKD1	-0.587	2.03E-03
SAMD11	-0.588	1.99E-03
FH	-0.588	1.98E-03
LPIN1	-0.588	2.00E-03
PLOD2	-0.588	2.00E-03
NAP1L1	-0.588	1.98E-03
KIF1C	-0.588	1.97E-03
PIAS2	-0.588	2.00E-03
MIR155HG	-0.588	1.98E-03
NOL12	-0.588	1.97E-03
NBPF15	-0.589	1.96E-03
KLHL18	-0.589	1.94E-03
R3HDM1	-0.59	1.91E-03
PGBD1	-0.59	1.89E-03
ST6GALNAC4	-0.59	1.92E-03
PCGF6	-0.59	1.91E-03
KIAA1524	-0.591	1.86E-03
ECT2	-0.591	1.85E-03
XXbac-BPG299F13.17	-0.591	1.89E-03
GNG11	-0.591	1.87E-03
UAP1L1	-0.591	1.88E-03
ACBD7	-0.591	1.88E-03
CEP55	-0.591	1.87E-03
RAB11FIP2	-0.591	1.87E-03
RECQL	-0.591	1.86E-03
ZNF555	-0.591	1.85E-03
ARSK	-0.592	1.83E-03
HINT1	-0.592	1.81E-03
CKS2	-0.592	1.84E-03
8-Mar	-0.592	1.84E-03
PNMA1	-0.592	1.83E-03

TSR1	-0.592	1.83E-03
HPRT1	-0.592	1.84E-03
MPHOSPH9	-0.593	1.80E-03
EVI2A	-0.593	1.77E-03
SPIN2B	-0.593	1.78E-03
RP11-640M9.2	-0.594	1.76E-03
DPH3	-0.594	1.76E-03
SLC4A7	-0.594	1.74E-03
IFT122	-0.594	1.75E-03
RWDD1	-0.594	1.73E-03
HERC4	-0.594	1.76E-03
IPO7	-0.594	1.74E-03
RP11-253E3.3	-0.594	1.76E-03
IKBIP	-0.594	1.75E-03
ZIC5	-0.594	1.76E-03
CDH2	-0.594	1.75E-03
PHF20	-0.594	1.75E-03
PDGFC	-0.595	1.70E-03
TTC28-AS1	-0.595	1.71E-03
RP11-1148L6.5	-0.595	1.70E-03
POLR2L	-0.596	1.67E-03
AMDHD2	-0.596	1.66E-03
HACL1	-0.597	1.62E-03
NCEH1	-0.597	1.64E-03
FBXO10	-0.597	1.64E-03
PLCB4	-0.597	1.63E-03
DYRK3	-0.598	1.58E-03
GLI2	-0.598	1.58E-03
LNP1	-0.598	1.59E-03
DCUN1D4	-0.598	1.58E-03
RP11-359J14.2	-0.598	1.58E-03
TXNRD1	-0.598	1.58E-03
RFXAP	-0.598	1.58E-03
MAD2L2	-0.599	1.55E-03
THOC7	-0.599	1.56E-03
RAPGEF6	-0.599	1.56E-03
GABBR1	-0.599	1.57E-03
RPA1	-0.599	1.56E-03
EIF4A1	-0.599	1.57E-03
ENO1	-0.6	1.53E-03
BOP1	-0.6	1.54E-03
RASSF8	-0.6	1.53E-03
SMG6	-0.6	1.53E-03
RP11-1094M14.11	-0.6	1.51E-03

SLC35E4	-0.6	1.52E-03
RAB3B	-0.601	1.47E-03
WDR12	-0.601	1.50E-03
MRPS27	-0.601	1.49E-03
RP5-874C20.3	-0.601	1.47E-03
PPP2R5D	-0.601	1.50E-03
DEAF1	-0.601	1.47E-03
CCDC112	-0.603	1.42E-03
EXOSC8	-0.603	1.41E-03
ABCC4	-0.603	1.41E-03
TTC7B	-0.603	1.43E-03
RP11-371I1.2	-0.604	1.38E-03
PHF7	-0.604	1.39E-03
OSTM1	-0.604	1.38E-03
PBX3	-0.604	1.39E-03
CD320	-0.604	1.39E-03
CCDC66	-0.605	1.35E-03
ZNF771	-0.605	1.37E-03
MYO1C	-0.605	1.35E-03
LINC00707	-0.606	1.31E-03
PARP11	-0.606	1.33E-03
CDC27	-0.606	1.33E-03
SPIRE1	-0.606	1.32E-03
MALT1	-0.606	1.31E-03
KCTD17	-0.606	1.32E-03
UAP1	-0.607	1.30E-03
ICK	-0.607	1.31E-03
RAB32	-0.607	1.29E-03
BICD1	-0.607	1.31E-03
RBPMS2	-0.607	1.31E-03
FHL1	-0.607	1.30E-03
AMPD2	-0.608	1.25E-03
GPATCH11	-0.608	1.25E-03
ALS2	-0.608	1.26E-03
PVRL3	-0.608	1.27E-03
SUV39H2	-0.608	1.26E-03
RP11-108P20.1	-0.608	1.25E-03
TMCC1-AS1	-0.609	1.23E-03
SCN1B	-0.609	1.23E-03
C9orf40	-0.61	1.21E-03
PTRF	-0.61	1.21E-03
COX11	-0.61	1.21E-03
PDCD5	-0.61	1.20E-03
SMS	-0.61	1.20E-03

POLA1	-0.61	1.19E-03
VPS26A	-0.611	1.17E-03
LRP12	-0.612	1.16E-03
QSOX2	-0.612	1.14E-03
TRDMT1	-0.613	1.13E-03
LOXL2	-0.614	1.10E-03
ABL1	-0.614	1.11E-03
GNB3	-0.614	1.09E-03
DSTNP2	-0.614	1.09E-03
SPG20	-0.614	1.10E-03
IDH3G	-0.615	1.06E-03
RAD18	-0.616	1.05E-03
RBMS3	-0.616	1.03E-03
RECK	-0.616	1.06E-03
FLNA	-0.617	1.03E-03
SGCB	-0.618	1.00E-03
DPYSL2	-0.618	9.83E-04
CLPP	-0.618	9.98E-04
EIF2B3	-0.619	9.72E-04
ODC1	-0.619	9.59E-04
RP11-134K13.2	-0.619	9.71E-04
RP11-1391J7.1	-0.619	9.65E-04
CEP164	-0.619	9.75E-04
FOXD1	-0.62	9.53E-04
PFDN1	-0.62	9.58E-04
ASAP1	-0.62	9.53E-04
TMEFF1	-0.62	9.54E-04
CEP97	-0.621	9.20E-04
ARHGEF10	-0.621	9.15E-04
CHST3	-0.621	9.19E-04
DPY19L2	-0.621	9.27E-04
STARD8	-0.621	9.26E-04
BCORL1	-0.621	9.28E-04
AKT3	-0.622	8.96E-04
CTGF	-0.622	8.91E-04
GFRA1	-0.622	9.06E-04
PFKM	-0.622	9.10E-04
NTHL1	-0.622	9.10E-04
WRAP53	-0.622	8.97E-04
CD70	-0.622	9.11E-04
DNAJB4	-0.623	8.73E-04
ABL2	-0.623	8.70E-04
APPL1	-0.623	8.83E-04
SLC35B4	-0.623	8.69E-04

PHF19	-0.623	8.77E-04
NUDC	-0.624	8.66E-04
GLYCTK	-0.624	8.62E-04
COL8A1	-0.624	8.59E-04
HMGN4	-0.624	8.60E-04
MSANTD3	-0.624	8.67E-04
WNT5B	-0.624	8.50E-04
GAS6	-0.624	8.63E-04
MRPL28	-0.624	8.68E-04
FGF5	-0.625	8.39E-04
DNAJB5	-0.625	8.46E-04
ACAT1	-0.625	8.38E-04
C1QL1	-0.625	8.41E-04
USP32	-0.625	8.47E-04
AP005482.1	-0.625	8.44E-04
ST3GAL3	-0.626	8.07E-04
DYNC1LI1	-0.626	8.16E-04
CCNH	-0.626	8.16E-04
FAM53C	-0.626	8.18E-04
ILK	-0.626	8.12E-04
TMEM160	-0.626	8.18E-04
NAA10	-0.626	8.23E-04
DFFA	-0.627	8.04E-04
VPRBP	-0.627	7.92E-04
ZBTB47	-0.628	7.77E-04
NCR3LG1	-0.628	7.71E-04
LGALS1	-0.628	7.75E-04
AK5	-0.629	7.66E-04
OXSM	-0.629	7.59E-04
ARSJ	-0.629	7.59E-04
TANGO6	-0.629	7.51E-04
SLMO1	-0.629	7.51E-04
SHOX2	-0.63	7.41E-04
ZFHX4-AS1	-0.63	7.40E-04
MMP16	-0.63	7.34E-04
APBB1	-0.63	7.45E-04
ASPHD1	-0.63	7.36E-04
THOC5	-0.63	7.38E-04
BBS5	-0.631	7.16E-04
RRP9	-0.631	7.16E-04
SPHK1	-0.631	7.14E-04
FER	-0.632	7.06E-04
WT1	-0.632	6.94E-04
CKAP5	-0.632	6.97E-04

NNMT	-0.632	6.96E-04
VKORC1	-0.632	7.07E-04
MAP3K3	-0.632	6.94E-04
FAM127A	-0.632	7.09E-04
FBXO18	-0.633	6.86E-04
BNIP3	-0.633	6.77E-04
PTPRJ	-0.633	6.85E-04
RANGRF	-0.633	6.92E-04
NUMBL	-0.633	6.92E-04
FAM50A	-0.633	6.89E-04
HDGFRP2	-0.634	6.61E-04
ADSL	-0.634	6.59E-04
GDI1	-0.634	6.70E-04
PDE12	-0.635	6.46E-04
LARS	-0.635	6.46E-04
FGD1	-0.635	6.57E-04
RPL13P5	-0.636	6.33E-04
DSEL	-0.636	6.33E-04
PREX1	-0.636	6.26E-04
WDR35	-0.637	6.13E-04
PFN2	-0.637	6.20E-04
FAM168A	-0.637	6.21E-04
SSX2IP	-0.638	6.03E-04
SDPR	-0.638	6.06E-04
RPL26L1	-0.638	6.07E-04
GPR3	-0.639	5.87E-04
ZNF438	-0.639	5.89E-04
CCDC85B	-0.639	5.91E-04
DIXDC1	-0.639	5.92E-04
CSF1	-0.64	5.68E-04
AC018737.1	-0.64	5.66E-04
PXK	-0.64	5.63E-04
ACTR1A	-0.641	5.59E-04
TBC1D24	-0.641	5.59E-04
PRR7	-0.642	5.46E-04
TMEM136	-0.642	5.45E-04
MSN	-0.642	5.35E-04
MECP2	-0.642	5.35E-04
ABHD6	-0.643	5.23E-04
GNG12	-0.644	5.08E-04
KCNIP3	-0.644	5.17E-04
EIF5A2	-0.644	5.12E-04
DPF3	-0.644	5.16E-04
TBC1D25	-0.644	5.10E-04

RP11-798M19.6	-0.646	4.92E-04
GEM	-0.646	4.86E-04
FAM92A1	-0.647	4.71E-04
PRKCA	-0.647	4.70E-04
CMSS1	-0.648	4.62E-04
RSU1	-0.648	4.57E-04
HMG20B	-0.648	4.63E-04
MON1A	-0.649	4.44E-04
TBC1D4	-0.649	4.49E-04
FOPNL	-0.649	4.44E-04
GJC1	-0.649	4.50E-04
SCML1	-0.649	4.47E-04
AZI2	-0.65	4.39E-04
RGMB	-0.65	4.34E-04
BDNF	-0.65	4.40E-04
FAM92A1P1	-0.65	4.34E-04
SAC3D1	-0.651	4.21E-04
NFIX	-0.651	4.29E-04
RP11-412D9.4	-0.652	4.16E-04
CPNE2	-0.652	4.11E-04
PCBP4	-0.653	4.05E-04
DLG4	-0.653	3.97E-04
CTDNEP1	-0.653	4.00E-04
C5orf24	-0.654	3.94E-04
ZNF804A	-0.655	3.80E-04
RP11-350N15.4	-0.655	3.81E-04
LURAP1	-0.656	3.66E-04
ELP6	-0.656	3.67E-04
TRPC1	-0.656	3.69E-04
ERCC2	-0.656	3.68E-04
SH2D5	-0.657	3.59E-04
FAM196B	-0.657	3.57E-04
ST3GAL2	-0.657	3.64E-04
PHF23	-0.657	3.56E-04
MATR3	-0.659	3.41E-04
PTMS	-0.659	3.36E-04
NDRG3	-0.66	3.34E-04
YEATS2	-0.661	3.26E-04
FXR2	-0.661	3.24E-04
C19orf44	-0.662	3.16E-04
PKIG	-0.662	3.16E-04
DZIP1L	-0.663	3.04E-04
SPATA5	-0.663	3.06E-04
BCKDK	-0.663	3.06E-04

SH2B3	-0.664	2.94E-04
MAP7D3	-0.666	2.79E-04
GPR176	-0.667	2.70E-04
OSBPL6	-0.668	2.66E-04
ZNF589	-0.668	2.64E-04
PIP4K2A	-0.668	2.66E-04
ARL2	-0.67	2.50E-04
ATP8B3	-0.671	2.39E-04
DPY19L2P2	-0.672	2.33E-04
CORO1C	-0.672	2.32E-04
CNTROB	-0.673	2.26E-04
EMD	-0.673	2.30E-04
MPP1	-0.673	2.24E-04
DNLZ	-0.674	2.20E-04
ERC1	-0.674	2.22E-04
CTC-308K20.1	-0.675	2.13E-04
ZSCAN16-AS1	-0.675	2.17E-04
ARRB2	-0.675	2.15E-04
CLTCL1	-0.675	2.12E-04
GEMIN5	-0.676	2.07E-04
TUBA1A	-0.677	2.02E-04
CCDC88A	-0.678	1.94E-04
TCF4	-0.68	1.83E-04
DNAJC18	-0.681	1.78E-04
BNC2	-0.683	1.69E-04
ST6GALNAC6	-0.683	1.71E-04
HARS	-0.684	1.64E-04
SPDL1	-0.684	1.64E-04
PNMA2	-0.684	1.63E-04
MRPL22	-0.685	1.58E-04
BMI1	-0.685	1.58E-04
MICU1	-0.685	1.57E-04
FAT4	-0.686	1.55E-04
MYBL1	-0.686	1.56E-04
MAP3K12	-0.686	1.54E-04
EID3	-0.686	1.53E-04
PPIP5K2	-0.687	1.50E-04
DVL2	-0.687	1.49E-04
RRP8	-0.688	1.45E-04
BOLA3-AS1	-0.689	1.38E-04
KCTD7	-0.689	1.41E-04
COQ10A	-0.689	1.38E-04
B4GALNT1	-0.689	1.38E-04
PIN1	-0.689	1.39E-04

UROD	-0.691	1.31E-04
RP11-156P1.3	-0.691	1.29E-04
NEIL3	-0.692	1.26E-04
STXBP1	-0.692	1.28E-04
COPRS	-0.694	1.19E-04
ZNF428	-0.694	1.20E-04
CDKN2C	-0.695	1.16E-04
CSGALNACT2	-0.696	1.10E-04
SATB2	-0.697	1.07E-04
ECI2	-0.697	1.09E-04
AAED1	-0.697	1.07E-04
MICAL3	-0.697	1.10E-04
ENO2	-0.698	1.06E-04
DPYD	-0.699	1.03E-04
SUSD5	-0.699	1.02E-04
FERMT2	-0.7	9.84E-05
FAM64A	-0.7	9.96E-05
PDHB	-0.701	9.44E-05
MAPK8IP1	-0.701	9.44E-05
MRC2	-0.702	9.33E-05
KIAA1279	-0.703	8.86E-05
SNAPC2	-0.703	8.96E-05
ANKRD28	-0.704	8.46E-05
LEPRE1	-0.706	7.92E-05
ABI2	-0.706	8.04E-05
BAG2	-0.706	7.93E-05
PGM5P2	-0.706	8.10E-05
CCDC77	-0.707	7.82E-05
HSPA8	-0.708	7.62E-05
KDM4A-AS1	-0.709	7.34E-05
POLR3G	-0.711	6.73E-05
FMNL3	-0.711	6.84E-05
PHF17	-0.713	6.37E-05
CAP2	-0.713	6.38E-05
ZEB1-AS1	-0.713	6.36E-05
FUT11	-0.714	6.07E-05
SYNC	-0.715	5.87E-05
LL22NC03-2H8.4	-0.715	5.91E-05
NFIC	-0.718	5.33E-05
NDUFAF3	-0.719	5.10E-05
MRPL41	-0.719	5.10E-05
PDDC1	-0.72	4.88E-05
TLN1	-0.721	4.78E-05
CNRIP1	-0.724	4.24E-05

MAP4	-0.725	4.19E-05
AC005624.2	-0.726	4.00E-05
GPR173	-0.731	3.29E-05
FAM21C	-0.732	3.19E-05
KIF18A	-0.732	3.23E-05
ZNF569	-0.732	3.20E-05
USP13	-0.736	2.74E-05
RP1-167A14.2	-0.739	2.48E-05
HABP4	-0.739	2.50E-05
ZFHX4	-0.74	2.34E-05
AP1S2	-0.74	2.32E-05
KATNAL1	-0.743	2.09E-05
LINC00094	-0.745	1.93E-05
ZHX3	-0.747	1.80E-05
RP11-145M9.4	-0.748	1.74E-05
RASA3	-0.748	1.71E-05
VIM	-0.75	1.57E-05
PTPRG	-0.751	1.55E-05
NXPE3	-0.751	1.51E-05
KLF2	-0.751	1.51E-05
SLC4A8	-0.754	1.32E-05
EMP3	-0.754	1.33E-05
PRR16	-0.755	1.30E-05
TRIM3	-0.755	1.31E-05
PYGO1	-0.755	1.31E-05
DOCK10	-0.757	1.16E-05
MIR137HG	-0.758	1.12E-05
CEP170	-0.758	1.12E-05
PFAS	-0.758	1.14E-05
EID2B	-0.759	1.11E-05
TTC28	-0.761	1.00E-05
NEXN	-0.763	9.19E-06
SEC22C	-0.763	9.29E-06
MAP1B	-0.763	9.24E-06
RP11-620J15.3	-0.765	8.60E-06
MAMLD1	-0.768	7.52E-06
ANXA6	-0.77	6.76E-06
FAM216A	-0.772	6.11E-06
CD99L2	-0.772	6.10E-06
DCLK2	-0.773	5.87E-06
RIC8B	-0.773	5.91E-06
MLTK	-0.774	5.61E-06
GNB4	-0.775	5.47E-06
ZEB2	-0.776	5.12E-06

SMAD9	-0.778	4.73E-06
TSPAN4	-0.781	4.00E-06
TTL	-0.783	3.68E-06
C17orf67	-0.783	3.65E-06
PDXP	-0.783	3.68E-06
PRR7-AS1	-0.784	3.58E-06
SGTB	-0.787	3.12E-06
CTD-2195B23.3	-0.789	2.71E-06
FAM171A1	-0.79	2.58E-06
MEX3B	-0.79	2.59E-06
FGF2	-0.796	1.94E-06
KCTD6	-0.8	1.62E-06
NMT2	-0.801	1.52E-06
DENND5A	-0.804	1.26E-06
FGFR1	-0.809	1.00E-06
CFL2	-0.809	1.00E-06
RP1-152L7.5	-0.81	9.40E-07
SACS	-0.815	6.86E-07
STX2	-0.817	6.40E-07
PMP22	-0.818	6.00E-07
CNTNAP1	-0.818	5.84E-07
C1orf216	-0.82	5.21E-07
AC007620.3	-0.82	5.28E-07
SOX12	-0.826	3.78E-07
VIM-AS1	-0.827	3.35E-07
AP1M1	-0.836	1.92E-07
LIX1L	-0.841	1.39E-07
CHN1	-0.841	1.39E-07
MSRB3	-0.842	1.33E-07
AC108463.2	-0.844	1.13E-07
FSD1L	-0.848	8.89E-08
STARD9	-0.849	7.93E-08
SAP30	-0.852	6.50E-08
DENND5B	-0.853	6.17E-08
FBXO43	-0.872	1.41E-08
GBE1	-0.883	5.14E-09
AC108463.1	-0.885	4.46E-09
ZEB1	-0.918	1.01E-10
SYDE1	-0.92	7.89E-11

Note: Genes that harbor a correlation coefficient ＞0.5 or ＜-0.5 and P-value ＜0.01 were identified.

Supplementary Table S7. The GSEA results for *CDH1* in BC cell lines. The most significantly enriched signal transduction pathways enriched in phenotype of high and low expression of *CDH1* in BC cell lines.

**Gene set name**	**ES**	**NES**	**adj. *p*-value**	**FDR**
**The most significantly gene sets enriched in phenotype of high expression of CDH1 in BCa cell lines**				
Alpha linolenic acid metabolism	0.789	1.644	0.000	0.346
Linoleic acid metabolism	0.670	1.547	0.027	0.541
**The most significantly gene sets enriched in phenotype of low expression of CDH1 in BCa cell lines**				
Aminoacyl tRNA biosynthesis	-0.618	-1.669	0.008	0.482
Glyoxylate and dicarboxylate metabolism	-0.6945	-1.656	0.0144	0.293
Cysteine and methionine metabolism	-0.580	-1.622	0.002	0.281
Basal transcription factors	-0.572	-1.590	0.016	0.299
Selenoamino acid metabolism	-0.578	-1.586	0.018	0.248
Cell cycle	-0.555	-1.564	0.038	0.257
RNA degradation	-0.584	-1.558	0.036	0.234
Pyrimidine metabolism	-0.528	-1.552	0.029	0.218

ES: enrichment score; NES: normalized enrichment score; adj: adjusted; FDR: false discovery rate.
Gene sets with adj. *p*-value are considered as significant.

Supplementary Table S8. The miRNAs related to the ten hub genes were predicted by intersecting the results obtained from TargetScan and miRDB databases.

CDH1	ACTA2	COL3A1	TPM1	ACTC1	ACTN1	IGFBP3	PPARG	SDC1	EPCAM
hsa-miR-4796-3p	hsa-miR-205-3p	hsa-miR-29c-3p	hsa-miR-4740-5p	hsa-miR-6847-5p	hsa-miR-4531	hsa-miR-374a-5p	hsa-miR-548n	hsa-miR-302e	hsa-miR-4307
hsa-miR-219a-5p	hsa-miR-3942-3p	hsa-miR-29b-3p	hsa-miR-302d-5p	hsa-miR-95-5p	hsa-miR-129-5p	hsa-miR-944	hsa-miR-548d-5p	hsa-miR-302a-3p	hsa-miR-33a-3p
hsa-miR-4282	hsa-miR-27b-3p	hsa-miR-5682	hsa-miR-302b-5p	hsa-miR-92b-3p	hsa-miR-889-3p	hsa-miR-374b-5p	hsa-miR-548y	hsa-miR-302b-3p	hsa-miR-4318
hsa-miR-6766-3p	hsa-miR-27a-3p	hsa-miR-29a-3p	hsa-miR-558	hsa-miR-29b-1-5p	hsa-miR-6880-5p	hsa-miR-19a-3p	hsa-miR-548h-5p	hsa-miR-302d-3p	hsa-miR-524-5p
hsa-miR-4782-3p	hsa-miR-6069	hsa-miR-6871-3p	hsa-miR-3169	hsa-miR-92a-3p	hsa-miR-2355-3p	hsa-miR-19b-3p	hsa-miR-548au-5p	hsa-miR-520c-3p	hsa-miR-520d-5p
hsa-miR-6128	hsa-miR-6124	hsa-miR-767-5p	hsa-miR-155-3p	hsa-miR-32-5p	hsa-miR-1236-3p	hsa-miR-4699-3p	hsa-miR-548a-5p	hsa-miR-520d-3p	hsa-miR-466
hsa-miR-3658	hsa-miR-513a-5p	hsa-miR-4500	hsa-miR-4255	hsa-miR-367-3p	hsa-miR-3163	hsa-miR-340-5p	hsa-miR-548i	hsa-miR-373-3p	hsa-miR-4789-3p
hsa-miR-627-3p	hsa-miR-128-3p	hsa-miR-98-5p	hsa-miR-548n	hsa-miR-363-3p	hsa-miR-676-3p	hsa-miR-3670	hsa-miR-548as-5p	hsa-miR-520a-3p	hsa-miR-4328
hsa-miR-544a	hsa-miR-216a-3p	hsa-miR-4458	hsa-miR-548am-5p	hsa-miR-25-3p	hsa-miR-6889-3p	hsa-miR-2113	hsa-miR-548w	hsa-miR-372-3p	hsa-miR-6839-5p
hsa-miR-4668-5p	hsa-miR-605-3p	hsa-let-7i-5p	hsa-miR-548i	hsa-miR-30a-5p	hsa-miR-545-5p	hsa-miR-3163	hsa-miR-548ar-5p	hsa-miR-19a-3p	hsa-miR-5692a
hsa-miR-378g	hsa-miR-3681-3p	hsa-let-7b-5p	hsa-miR-548o-5p	hsa-miR-30d-5p	hsa-miR-4456	hsa-miR-197-3p	hsa-miR-548ab	hsa-miR-4731-5p	hsa-miR-1290
hsa-miR-372-5p	hsa-miR-4524a-3p	hsa-let-7c-5p	hsa-miR-548ap-5p	hsa-miR-30e-5p	hsa-miR-19a-3p	hsa-miR-1468-3p	hsa-miR-548ap-5p	hsa-miR-19b-3p	hsa-miR-590-3p
hsa-miR-5003-3p	hsa-miR-5000-3p	hsa-let-7g-5p	hsa-miR-548ay-5p	hsa-miR-30c-5p	hsa-miR-2113	hsa-miR-4671-5p	hsa-miR-548o-5p	hsa-miR-1207-5p	hsa-miR-378j
hsa-miR-510-5p	hsa-miR-4436a	hsa-let-7a-5p	hsa-miR-548ae-5p	hsa-miR-30b-5p	hsa-miR-6881-3p	hsa-miR-3910	hsa-miR-548ay-5p	hsa-miR-548f-5p	hsa-miR-4705
hsa-miR-7106-5p	hsa-miR-4477a	hsa-let-7f-5p	hsa-miR-548ar-5p	hsa-miR-340-5p	hsa-miR-19b-3p	hsa-miR-1910-3p	hsa-miR-548ae-5p	hsa-miR-9500	hsa-miR-1289
hsa-miR-548t-5p	hsa-miR-3978	hsa-let-7d-5p	hsa-miR-548as-5p	hsa-miR-4793-5p	hsa-miR-5695	hsa-miR-548aw	hsa-miR-548am-5p	hsa-miR-212-5p	hsa-miR-4775
hsa-miR-548az-5p	hsa-miR-5702	hsa-let-7e-5p	hsa-miR-548ab	hsa-miR-5688	hsa-miR-636	hsa-miR-9-5p	hsa-miR-548aq-5p	hsa-miR-548g-5p	hsa-miR-4432
hsa-miR-1273h-5p	hsa-miR-6818-5p	hsa-miR-6759-3p	hsa-miR-548w	hsa-miR-3119	hsa-miR-502-3p	hsa-miR-6511a-5p	hsa-miR-548bb-5p	hsa-miR-548aj-5p	hsa-miR-3942-3p
hsa-miR-1207-5p		hsa-miR-3942-3p	hsa-miR-548au-5p	hsa-miR-3145-5p	hsa-miR-501-3p	hsa-miR-4684-5p	hsa-miR-548j-5p	hsa-miR-548x-5p	hsa-miR-6079
hsa-miR-23a-3p		hsa-miR-548t-5p	hsa-miR-548y	hsa-miR-495-3p	hsa-miR-548g-3p	hsa-miR-548x-3p	hsa-miR-548ak	hsa-miR-6512-3p	hsa-miR-4755-5p
hsa-miR-23c		hsa-miR-548az-5p	hsa-miR-548a-5p	hsa-miR-299-5p	hsa-miR-7852-3p	hsa-miR-548aj-3p	hsa-miR-559	hsa-miR-597-3p	hsa-miR-5006-3p
hsa-miR-23b-3p		hsa-miR-205-3p	hsa-miR-548ad-5p	hsa-miR-4422	hsa-miR-449b-3p	hsa-miR-548at-5p	hsa-miR-548c-5p	hsa-miR-6720-5p	hsa-let-7c-3p
hsa-miR-9-3p		hsa-miR-665	hsa-miR-548b-5p	hsa-miR-142-5p	hsa-miR-374a-5p	hsa-miR-548f-5p	hsa-miR-548b-5p	hsa-miR-196a-3p	hsa-miR-6828-5p
hsa-miR-4486		hsa-miR-3613-3p	hsa-miR-548j-5p	hsa-miR-3942-5p	hsa-miR-374b-5p	hsa-miR-548aj-5p	hsa-miR-548ad-5p	hsa-miR-4776-3p	hsa-miR-4719
hsa-miR-6780a-5p		hsa-miR-219a-2-3p	hsa-miR-548d-5p	hsa-miR-5580-3p	hsa-miR-3065-5p	hsa-miR-548g-5p	hsa-miR-3606-3p	hsa-miR-548e-5p	hsa-miR-3121-3p
hsa-miR-3163		hsa-miR-4670-3p	hsa-miR-548bb-5p	hsa-miR-4703-5p	hsa-miR-450b-5p	hsa-miR-4696	hsa-miR-27b-3p	hsa-miR-4763-3p	hsa-miR-6759-3p
hsa-miR-30b-3p		hsa-miR-520d-5p	hsa-miR-548aq-5p	hsa-miR-5696	hsa-miR-5692b	hsa-miR-548x-5p	hsa-miR-27a-3p	hsa-miR-10a-5p	hsa-miR-4782-5p
hsa-miR-3689c		hsa-miR-380-5p	hsa-miR-548h-5p	hsa-miR-4297	hsa-miR-3680-3p	hsa-miR-548am-3p	hsa-miR-513a-3p	hsa-miR-10b-5p	hsa-miR-5706
hsa-miR-3689b-3p		hsa-miR-4474-3p	hsa-miR-548ak	hsa-miR-3943	hsa-miR-5692c	hsa-miR-548j-3p	hsa-miR-513c-3p	hsa-miR-561-3p	hsa-miR-3190-3p
hsa-miR-3689a-3p		hsa-miR-545-3p	hsa-miR-548c-5p	hsa-miR-4760-3p	hsa-miR-369-3p	hsa-miR-7154-3p	hsa-miR-3617-3p	hsa-miR-3125	hsa-miR-513a-3p

hsa-miR-6779-5p		hsa-miR-524-5p	hsa-miR-559	hsa-miR-6844	hsa-miR-507	hsa-miR-548ae-3p	hsa-miR-5694	hsa-miR-6822-5p	hsa-miR-3606-3p
hsa-miR-7977		hsa-miR-651-3p	hsa-miR-4528	hsa-miR-4724-5p	hsa-miR-218-5p	hsa-miR-548ah-3p	hsa-miR-454-3p	hsa-miR-4673	hsa-miR-513c-3p
hsa-miR-5688		hsa-miR-6514-5p	hsa-let-7b-3p	hsa-miR-6835-3p	hsa-miR-5692a	hsa-miR-548aq-3p	hsa-miR-4295	hsa-miR-6859-5p	hsa-miR-567
hsa-miR-4677-5p		hsa-miR-4762-3p	hsa-miR-98-3p	hsa-miR-548g-3p	hsa-miR-4516	hsa-miR-4427	hsa-miR-130b-3p	hsa-miR-5584-5p	hsa-miR-4693-5p
hsa-miR-7-1-3p		hsa-miR-6844	hsa-let-7f-1-3p	hsa-miR-5590-3p	hsa-miR-557	hsa-miR-3159	hsa-miR-130a-3p	hsa-miR-3916	hsa-miR-297
hsa-miR-1323		hsa-miR-4653-3p	hsa-let-7a-3p	hsa-miR-190a-5p	hsa-miR-8071	hsa-miR-4770	hsa-miR-301b-3p	hsa-miR-4531	hsa-miR-1297
hsa-miR-7-2-3p		hsa-miR-548e-5p	hsa-miR-4639-5p	hsa-let-7c-3p	hsa-miR-3165	hsa-miR-6088	hsa-miR-301a-3p	hsa-miR-6750-5p	hsa-miR-509-3p
hsa-miR-495-3p		hsa-miR-2113	hsa-miR-297	hsa-miR-5581-5p	hsa-miR-4434	hsa-miR-143-3p	hsa-miR-3666	hsa-miR-152-5p	hsa-miR-5088-3p
hsa-miR-4433a-3p		hsa-miR-4504	hsa-miR-3936	hsa-miR-376c-3p	hsa-miR-5703	hsa-miR-3173-3p	hsa-miR-586	hsa-miR-5004-3p	hsa-miR-7705
hsa-miR-6765-3p		hsa-miR-3192-3p	hsa-miR-3606-5p	hsa-miR-551b-5p	hsa-miR-4739	hsa-miR-146a-3p	hsa-miR-4446-5p	hsa-miR-182-3p	hsa-miR-5688
hsa-miR-3609		hsa-miR-563	hsa-miR-219a-5p	hsa-miR-5008-3p	hsa-miR-374c-5p	hsa-miR-6818-5p	hsa-miR-548t-5p	hsa-miR-519a-3p	hsa-miR-4676-5p
hsa-miR-1285-3p		hsa-miR-153-5p	hsa-miR-4782-3p	hsa-miR-7157-3p	hsa-miR-5186	hsa-miR-6891-5p	hsa-miR-4671-3p	hsa-miR-519b-3p	hsa-miR-575
hsa-miR-5189-5p		hsa-miR-3163	hsa-miR-3149	hsa-miR-6737-3p	hsa-miR-655-3p	hsa-miR-1290	hsa-miR-548az-5p	hsa-miR-6828-5p	hsa-miR-6854-5p
hsa-miR-340-5p		hsa-miR-3194-3p	hsa-miR-6766-3p	hsa-miR-371b-5p	hsa-miR-548c-3p	hsa-miR-4260	hsa-miR-3154	hsa-miR-519c-3p	hsa-miR-205-3p
hsa-miR-383-3p		hsa-miR-3908	hsa-miR-4692	hsa-miR-616-5p	hsa-miR-16-2-3p	hsa-miR-4666a-3p	hsa-miR-889-3p	hsa-miR-4645-5p	hsa-miR-148b-5p
hsa-miR-4684-5p		hsa-miR-3148	hsa-miR-2115-3p	hsa-miR-373-5p	hsa-miR-195-3p	hsa-miR-3679-3p	hsa-miR-7515	hsa-miR-1468-3p	hsa-miR-6874-3p
hsa-miR-302c-5p		hsa-miR-4256	hsa-miR-4514	hsa-miR-139-5p	hsa-miR-550b-2-5p	hsa-miR-3065-5p	hsa-miR-5583-3p	hsa-miR-4286	
hsa-miR-580-3p		hsa-miR-4517	hsa-miR-3182	hsa-miR-4684-3p	hsa-miR-4756-5p	hsa-miR-5582-3p	hsa-miR-4711-3p	hsa-miR-22-5p	
hsa-miR-1225-3p		hsa-miR-585-5p	hsa-miR-4666a-3p	hsa-miR-4692	hsa-miR-1321	hsa-miR-2117	hsa-miR-590-3p	hsa-miR-1910-3p	
hsa-miR-3065-3p		hsa-miR-3928-3p	hsa-miR-8064	hsa-miR-7703	hsa-miR-30e-5p	hsa-miR-5010-3p	hsa-miR-338-5p	hsa-miR-548aw	
hsa-miR-3187-5p		hsa-miR-3663-3p	hsa-miR-4445-5p	hsa-miR-6888-3p	hsa-miR-30d-5p	hsa-miR-34a-5p	hsa-miR-3163	hsa-miR-6887-3p	
hsa-miR-6860		hsa-miR-5691	hsa-miR-3622b-3p	hsa-let-7g-3p	hsa-miR-3678-3p	hsa-miR-548au-3p	hsa-miR-513a-5p	hsa-miR-8485	
hsa-miR-4306		hsa-miR-128-3p	hsa-miR-3622a-3p	hsa-miR-5010-5p	hsa-miR-30a-5p	hsa-miR-449a	hsa-miR-545-5p	hsa-miR-6794-3p	
hsa-miR-6715b-5p		hsa-miR-6805-3p	hsa-miR-542-3p	hsa-miR-4525	hsa-miR-1273h-3p	hsa-miR-7159-5p	hsa-miR-4775	hsa-miR-6511a-5p	
hsa-miR-612		hsa-miR-216a-3p	hsa-let-7c-3p	hsa-let-7a-2-3p	hsa-miR-4763-3p	hsa-miR-593-3p	hsa-miR-335-3p	hsa-miR-4308	
hsa-miR-4690-5p		hsa-miR-3681-3p	hsa-miR-3665	hsa-miR-548x-3p	hsa-miR-4722-5p	hsa-miR-3153	hsa-miR-216a-3p	hsa-miR-6825-5p	
hsa-miR-4763-3p		hsa-miR-196a-5p	hsa-miR-3691-5p	hsa-miR-507	hsa-miR-30b-5p	hsa-miR-6733-5p	hsa-miR-128-3p	hsa-miR-6813-3p	
hsa-miR-3127-3p		hsa-miR-3120-3p	hsa-miR-4474-5p	hsa-miR-548aj-3p	hsa-miR-30c-5p	hsa-miR-6739-5p	hsa-miR-3140-3p	hsa-miR-6849-3p	
hsa-miR-548o-3p		hsa-miR-4422	hsa-miR-4520-3p	hsa-miR-570-3p	hsa-miR-1207-5p	hsa-miR-7161-3p	hsa-miR-3681-3p	hsa-miR-6504-3p	
hsa-miR-6756-3p		hsa-miR-196b-5p		hsa-miR-6072	hsa-miR-4311	hsa-miR-1276	hsa-miR-3121-3p	hsa-miR-548as-3p	
hsa-miR-616-5p		hsa-miR-548p		hsa-miR-4432	hsa-miR-6734-5p	hsa-miR-1908-3p	hsa-miR-1279	hsa-miR-520g-5p	
hsa-miR-6772-3p		hsa-miR-5089-5p		hsa-miR-4325	hsa-miR-550a-3-5p	hsa-miR-449b-5p	hsa-miR-340-5p	hsa-miR-4684-3p	
hsa-miR-548c-3p		hsa-miR-4425		hsa-miR-4666a-3p	hsa-miR-1271-3p	hsa-miR-34c-5p		hsa-miR-4511	

hsa-miR-149-3p		hsa-miR-7159-5p		hsa-miR-568	hsa-miR-298	hsa-miR-5692a		hsa-miR-3919
hsa-miR-3617-3p		hsa-miR-3145-3p		hsa-miR-5584-3p	hsa-miR-550a-5p	hsa-miR-659-3p		hsa-miR-665
hsa-miR-519e-5p		hsa-miR-3115		hsa-miR-5692a	hsa-miR-7106-5p	hsa-miR-5004-5p		hsa-miR-6721-5p
hsa-miR-4269		hsa-miR-4282		hsa-miR-2276-3p	hsa-miR-3912-5p	hsa-miR-6842-5p		hsa-miR-3157-5p
hsa-miR-6785-5p		hsa-miR-3659		hsa-miR-557	hsa-miR-4505	hsa-miR-7110-5p		hsa-miR-548v
hsa-miR-548ah-5p		hsa-miR-3978		hsa-miR-548am-3p	hsa-miR-5787	hsa-miR-4799-5p		hsa-miR-9-5p
hsa-miR-515-5p		hsa-miR-634		hsa-miR-5571-5p	hsa-miR-590-3p	hsa-miR-4635		hsa-miR-7974
hsa-miR-4728-5p		hsa-miR-5692a		hsa-miR-6891-3p	hsa-miR-633	hsa-miR-543		hsa-miR-561-5p
hsa-miR-199b-5p		hsa-miR-3189-5p		hsa-miR-548aq-3p	hsa-miR-4450	hsa-miR-6884-5p		hsa-miR-659-3p
hsa-miR-1299		hsa-miR-576-5p		hsa-miR-544a	hsa-miR-1228-3p	hsa-miR-6131		hsa-miR-1291
hsa-miR-4441		hsa-miR-582-3p		hsa-miR-4709-3p	hsa-miR-6894-5p	hsa-miR-548h-3p		hsa-miR-4736
hsa-miR-4270		hsa-miR-3146		hsa-miR-548ah-3p	hsa-miR-4267	hsa-miR-485-5p		hsa-miR-4530
hsa-miR-199a-5p		hsa-miR-4789-3p		hsa-miR-548j-3p	hsa-miR-4433b-3p	hsa-miR-548z		hsa-miR-556-3p
hsa-miR-3613-3p		hsa-miR-876-5p		hsa-miR-548ae-3p	hsa-miR-6770-5p	hsa-miR-548ac		hsa-miR-4514
hsa-miR-519d-5p		hsa-miR-3167		hsa-miR-4514	hsa-miR-4786-3p	hsa-miR-548bb-3p		hsa-miR-3133
hsa-miR-185-5p		hsa-miR-4311		hsa-miR-222-3p	hsa-miR-3124-3p	hsa-miR-616-5p		hsa-miR-6509-5p
hsa-miR-6883-5p		hsa-miR-6734-3p		hsa-miR-4799-5p	hsa-miR-548av-3p	hsa-miR-373-5p		hsa-miR-6795-5p
hsa-miR-6799-5p		hsa-miR-186-5p		hsa-miR-511-3p	hsa-miR-6867-5p	hsa-miR-371b-5p		hsa-miR-367-5p
hsa-miR-4644		hsa-miR-616-3p		hsa-miR-221-3p		hsa-miR-629-5p		hsa-miR-6887-5p
hsa-miR-4328		hsa-miR-5093		hsa-miR-200b-3p		hsa-miR-6858-3p		hsa-miR-4692
hsa-miR-4254		hsa-miR-6128		hsa-miR-429		hsa-miR-6752-5p		hsa-miR-4447
hsa-miR-5699-3p		hsa-miR-202-3p		hsa-miR-200c-3p		hsa-miR-4795-3p		hsa-miR-4685-5p
hsa-miR-551b-5p				hsa-miR-4715-3p		hsa-miR-1266-3p		hsa-miR-5580-5p
hsa-miR-20a-3p				hsa-miR-378a-5p		hsa-miR-548d-3p		hsa-miR-4756-3p
hsa-miR-4421				hsa-miR-938		hsa-miR-588		hsa-miR-6733-5p
hsa-miR-888-5p				hsa-miR-576-5p		hsa-miR-4701-5p		hsa-miR-6739-5p
hsa-miR-508-3p				hsa-miR-561-3p				hsa-miR-5701
hsa-miR-4711-5p				hsa-miR-7849-3p				hsa-miR-6775-3p
hsa-miR-320c				hsa-miR-8076				hsa-miR-6732-3p
hsa-miR-6787-3p				hsa-miR-548at-5p				hsa-miR-3139
hsa-miR-320b				hsa-miR-3171				hsa-miR-28-5p
hsa-miR-4429				hsa-miR-4527				hsa-miR-182-5p
hsa-miR-6501-3p				hsa-miR-7107-3p				hsa-miR-708-5p

hsa-miR-1237-3p				hsa-miR-6753-3p				hsa-miR-660-5p	
hsa-miR-320d				hsa-miR-6074				hsa-miR-4492	
hsa-miR-4500				hsa-miR-513c-5p				hsa-miR-548p	
hsa-miR-216b-3p				hsa-miR-500a-3p				hsa-miR-378g	
hsa-miR-373-5p				hsa-miR-4264				hsa-miR-4472	
hsa-miR-371b-5p				hsa-miR-7-5p				hsa-miR-4722-5p	
hsa-miR-4801				hsa-miR-8485				hsa-miR-4488	
hsa-miR-4445-5p				hsa-miR-514b-5p				hsa-miR-548ax	
hsa-miR-610				hsa-miR-5695				hsa-miR-548u	
hsa-miR-2355-5p				hsa-miR-548b-3p				hsa-miR-548ao-5p	
hsa-miR-7112-3p				hsa-miR-6071					
hsa-miR-4310				hsa-miR-5693					
				hsa-miR-892a					
				hsa-miR-6503-5p					
				hsa-miR-3614-3p					
				hsa-miR-6852-3p					
				hsa-miR-223-5p					
				hsa-miR-4272					
				hsa-miR-3182					

Supplementary Table S9. The miRNAs paired with DElncRNAs and hub genes. DEmiRNAs that interacted with both DElncRNAs and hub genes were identified, and 424 paired lncRNAs and miRNAs, and 59 paired hub genes and miRNAs were obtained. the miRNAs related to hub genes were identified by intersecting the results obtained form TargetScan and miRDB databases. The DElncRNAs and DEmiRNAs were identified in bladder cancer tissues comparing to adjacent noncancer bladder tissues from TCGA database.

**DElncRNA - DEmiRNA** (424 paired)		
**lncRNA**	**Category**	**miRNA**
PART1	Down-regulated gene	hsa-mir-301b
		hsa-mir-454
		hsa-mir-4295
		hsa-mir-3666
		hsa-mir-9
		hsa-mir-143
		hsa-mir-4770
		hsa-mir-152
		hsa-mir-155
		hsa-mir-195
		hsa-mir-429
		hsa-mir-548a
		hsa-mir-205
		hsa-mir-508
		hsa-mir-22
		hsa-mir-25
		hsa-mir-32
		hsa-mir-363
		hsa-mir-367
		hsa-mir-1297
		hsa-mir-128
C2orf48	Up-regulated gene	hsa-mir-7
		hsa-mir-372
		hsa-mir-373
		hsa-mir-519a
		hsa-mir-143
		hsa-mir-4770
		hsa-mir-155
		hsa-mir-195
		hsa-mir-519d
		hsa-mir-216a
		hsa-mir-223
		hsa-mir-338
		hsa-mir-128
AC009065.1	Up-regulated gene	hsa-mir-7
		hsa-mir-372
		hsa-mir-373
		hsa-mir-519a

**mRNA - DEmiRNA** (59 paired)		
**Hub gene （mRNA）**	**Category**	**miRNA**
CDH1	Up-regulated gene	hsa-mir-372
		hsa-mir-9
		hsa-mir-383
		hsa-mir-519d
		hsa-mir-508
		hsa-mir-4500
		hsa-mir-216b
		hsa-mir-373
ACTA2	Down-regulated gene	hsa-mir-205
		hsa-mir-128
		hsa-mir-216a
COL3A1	Down-regulated gene	hsa-mir-4500
		hsa-mir-98
		hsa-mir-4458
		hsa-mir-205
		hsa-mir-153
		hsa-mir-128
		hsa-mir-216a
TPM1	Down-regulated gene	hsa-mir-155
		hsa-mir-548a
		hsa-mir-98
ACTC1	Down-regulated gene	hsa-mir-32
		hsa-mir-367
		hsa-mir-363
		hsa-mir-25
		hsa-mir-373
		hsa-mir-507
		hsa-mir-222
		hsa-mir-221
		hsa-mir-429
		hsa-mir-7
		hsa-mir-223
ACTN1	Down-regulated gene	hsa-mir-507
		hsa-mir-218
		hsa-mir-195
		hsa-mir-1271
IGFBP3	Up-regulated gene	hsa-mir-9
		hsa-mir-4770

C20orf197	Up-regulated gene	hsa-mir-372<br>hsa-mir-373<br>hsa-mir-519a<br>hsa-mir-143<br>hsa-mir-4770<br>hsa-mir-519d<br>hsa-mir-218<br>hsa-mir-508<br>hsa-mir-25<br>hsa-mir-32<br>hsa-mir-363<br>hsa-mir-367<br>hsa-mir-338<br>hsa-mir-383
LINC00518	Up-regulated gene	hsa-mir-143<br>hsa-mir-4770<br>hsa-mir-216a<br>hsa-mir-216b<br>hsa-mir-128
LINC00308	Up-regulated gene	hsa-mir-519d<br>hsa-mir-205<br>hsa-mir-22<br>hsa-mir-338
LINC00482	Up-regulated gene	hsa-mir-7<br>hsa-mir-143<br>hsa-mir-4770<br>hsa-mir-22<br>hsa-mir-128
MIR22HG	Down-regulated gene	hsa-mir-25<br>hsa-mir-32<br>hsa-mir-363<br>hsa-mir-367<br>hsa-mir-383
LINC00221	Up-regulated gene	hsa-mir-301b<br>hsa-mir-454<br>hsa-mir-4295<br>hsa-mir-3666<br>hsa-mir-7<br>hsa-mir-9<br>hsa-mir-372<br>hsa-mir-373<br>hsa-mir-519a<br>hsa-mir-507<br>hsa-mir-1271<br>hsa-mir-143<br>hsa-mir-4770
		hsa-mir-143<br>hsa-mir-373
PPARG	Up-regulated gene	hsa-mir-548a<br>hsa-mir-454<br>hsa-mir-4295<br>hsa-mir-301b<br>hsa-mir-3666<br>hsa-mir-338<br>hsa-mir-216a<br>hsa-mir-128
SDC1	Up-regulated gene	hsa-mir-373<br>hsa-mir-372<br>hsa-mir-212<br>hsa-mir-152<br>hsa-mir-182<br>hsa-mir-519a<br>hsa-mir-22<br>hsa-mir-9<br>hsa-mir-367
EPCAM	Up-regulated gene	hsa-mir-1297<br>hsa-mir-205

		hsa-mir-152<br>hsa-mir-153<br>hsa-mir-519d<br>hsa-mir-182<br>hsa-mir-98<br>hsa-mir-4458<br>hsa-mir-4500<br>hsa-mir-508<br>hsa-mir-22<br>hsa-mir-25<br>hsa-mir-32<br>hsa-mir-363<br>hsa-mir-367<br>hsa-mir-1297<br>hsa-mir-338<br>hsa-mir-128
C9orf163	Up-regulated gene	hsa-mir-9<br>hsa-mir-143<br>hsa-mir-4770<br>hsa-mir-195<br>hsa-mir-205
LINC00336	Up-regulated gene	hsa-mir-7<br>hsa-mir-507<br>hsa-mir-1271<br>hsa-mir-143<br>hsa-mir-4770<br>hsa-mir-98<br>hsa-mir-4458<br>hsa-mir-4500<br>hsa-mir-216a<br>hsa-mir-216b<br>hsa-mir-128
LINC00487	Up-regulated gene	hsa-mir-9<br>hsa-mir-372<br>hsa-mir-373<br>hsa-mir-519a<br>hsa-mir-143<br>hsa-mir-4770<br>hsa-mir-152<br>hsa-mir-205<br>hsa-mir-216a<br>hsa-mir-216b<br>hsa-mir-338<br>hsa-mir-128
AC099805.1	Down-regulated gene	hsa-mir-7

AC133041.1	Up-regulated gene	hsa-mir-143<br>hsa-mir-4770
C7orf71	Up-regulated gene	hsa-mir-301b<br>hsa-mir-454<br>hsa-mir-4295<br>hsa-mir-3666<br>hsa-mir-9<br>hsa-mir-152<br>hsa-mir-155<br>hsa-mir-195<br>hsa-mir-508<br>hsa-mir-25<br>hsa-mir-32<br>hsa-mir-363<br>hsa-mir-367<br>hsa-mir-1297
LINC00473	Down-regulated gene	hsa-mir-195<br>hsa-mir-218<br>hsa-mir-338<br>hsa-mir-128
LINC00393	Up-regulated gene	hsa-mir-372<br>hsa-mir-373<br>hsa-mir-519a
LINC00337	Up-regulated gene	hsa-mir-212<br>hsa-mir-372<br>hsa-mir-373<br>hsa-mir-519a<br>hsa-mir-153<br>hsa-mir-519d<br>hsa-mir-182<br>hsa-mir-216a<br>hsa-mir-216b<br>hsa-mir-218<br>hsa-mir-338<br>hsa-mir-383<br>hsa-mir-128
LINC00351	Up-regulated gene	hsa-mir-155<br>hsa-mir-205
LINC00161	Down-regulated gene	hsa-mir-9<br>hsa-mir-205<br>hsa-mir-1297<br>hsa-mir-128
LINC00355	Up-regulated gene	hsa-mir-153<br>hsa-mir-195<br>hsa-mir-98<br>hsa-mir-4458

		hsa-mir-4500<br>hsa-mir-218<br>hsa-mir-223<br>hsa-mir-338<br>hsa-mir-128
LINC00392	Up-regulated gene	hsa-mir-7<br>hsa-mir-9<br>hsa-mir-153<br>hsa-mir-25<br>hsa-mir-32<br>hsa-mir-363<br>hsa-mir-367
HCG22	Down-regulated gene	hsa-mir-507<br>hsa-mir-1271<br>hsa-mir-195<br>hsa-mir-182<br>hsa-mir-216a<br>hsa-mir-216b<br>hsa-mir-218<br>hsa-mir-508<br>hsa-mir-22<br>hsa-mir-383<br>hsa-mir-128
SACS-AS1	Down-regulated gene	hsa-mir-372<br>hsa-mir-373<br>hsa-mir-519a<br>hsa-mir-143<br>hsa-mir-4770<br>hsa-mir-205<br>hsa-mir-218<br>hsa-mir-508<br>hsa-mir-22
ERVMER61-1	Up-regulated gene	hsa-mir-7<br>hsa-mir-507<br>hsa-mir-1271<br>hsa-mir-153<br>hsa-mir-182<br>hsa-mir-205<br>hsa-mir-1297<br>hsa-mir-338
MIR137HG	Up-regulated gene	hsa-mir-182<br>hsa-mir-98<br>hsa-mir-4458<br>hsa-mir-4500<br>hsa-mir-22<br>hsa-mir-25

		hsa-mir-32
		hsa-mir-363
		hsa-mir-367
		hsa-mir-338
		hsa-mir-128
ERVH48-1	Up-regulated gene	hsa-mir-301b
		hsa-mir-454
		hsa-mir-4295
		hsa-mir-3666
		hsa-mir-7
		hsa-mir-9
		hsa-mir-507
		hsa-mir-1271
		hsa-mir-182
		hsa-mir-98
		hsa-mir-4458
		hsa-mir-4500
		hsa-mir-508
		hsa-mir-22
		hsa-mir-223
		hsa-mir-338
		hsa-mir-128
LINC00472	Down-regulated gene	hsa-mir-372
		hsa-mir-373
		hsa-mir-519a
		hsa-mir-143
		hsa-mir-4770
		hsa-mir-155
		hsa-mir-195
		hsa-mir-216b
		hsa-mir-22
		hsa-mir-383
AC110491.1	Down-regulated gene	hsa-mir-7
		hsa-mir-9
		hsa-mir-143
		hsa-mir-4770
		hsa-mir-153
		hsa-mir-182
		hsa-mir-98
		hsa-mir-4458
		hsa-mir-4500
		hsa-mir-429
		hsa-mir-548a
		hsa-mir-205
		hsa-mir-216a
		hsa-mir-216b

		hsa-mir-218<br>hsa-mir-508<br>hsa-mir-22<br>hsa-mir-221<br>hsa-mir-222<br>hsa-mir-1297<br>hsa-mir-338<br>hsa-mir-128
AC005035.1	Up-regulated gene	hsa-mir-301b<br>hsa-mir-454<br>hsa-mir-4295<br>hsa-mir-3666<br>hsa-mir-212<br>hsa-mir-7<br>hsa-mir-9<br>hsa-mir-507<br>hsa-mir-1271<br>hsa-mir-155<br>hsa-mir-182<br>hsa-mir-429<br>hsa-mir-548a<br>hsa-mir-221<br>hsa-mir-222<br>hsa-mir-1297
LINC00460	Up-regulated gene	hsa-mir-143<br>hsa-mir-4770<br>hsa-mir-429<br>hsa-mir-548a<br>hsa-mir-221<br>hsa-mir-222<br>hsa-mir-338
LINC00462	Up-regulated gene	hsa-mir-9<br>hsa-mir-372<br>hsa-mir-373<br>hsa-mir-519a<br>hsa-mir-519d<br>hsa-mir-25<br>hsa-mir-32<br>hsa-mir-363<br>hsa-mir-367
LINC00163	Down-regulated gene	hsa-mir-7<br>hsa-mir-143<br>hsa-mir-4770<br>hsa-mir-128
LINC00330	Down-regulated gene	hsa-mir-301b<br>hsa-mir-454

		hsa-mir-4295<br>hsa-mir-3666<br>hsa-mir-372<br>hsa-mir-373<br>hsa-mir-519a<br>hsa-mir-195<br>hsa-mir-519d<br>hsa-mir-98<br>hsa-mir-4458<br>hsa-mir-4500<br>hsa-mir-205<br>hsa-mir-218<br>hsa-mir-383
LINC00402	Down-regulated gene	hsa-mir-7<br>hsa-mir-9<br>hsa-mir-143<br>hsa-mir-4770<br>hsa-mir-153<br>hsa-mir-155<br>hsa-mir-519d<br>hsa-mir-182<br>hsa-mir-429<br>hsa-mir-548a<br>hsa-mir-216a<br>hsa-mir-216b<br>hsa-mir-22<br>hsa-mir-1297<br>hsa-mir-338<br>hsa-mir-383<br>hsa-mir-128
AC112721.1	Up-regulated gene	hsa-mir-153<br>hsa-mir-195
AL139002.1	Up-regulated gene	hsa-mir-301b<br>hsa-mir-454<br>hsa-mir-4295<br>hsa-mir-3666<br>hsa-mir-143<br>hsa-mir-4770<br>hsa-mir-507<br>hsa-mir-1271<br>hsa-mir-182
AC011453.1	Up-regulated gene	hsa-mir-143<br>hsa-mir-4770<br>hsa-mir-205<br>hsa-mir-218
LINC00536	Up-regulated	hsa-mir-507

	gene	hsa-mir-1271<br>hsa-mir-195<br>hsa-mir-519d<br>hsa-mir-182<br>hsa-mir-205<br>hsa-mir-508<br>hsa-mir-22<br>hsa-mir-338
AC128709.1	Up-regulated gene	hsa-mir-152<br>hsa-mir-195<br>hsa-mir-98<br>hsa-mir-4458<br>hsa-mir-4500<br>hsa-mir-22<br>hsa-mir-1297<br>hsa-mir-338
LINC00534	Up-regulated gene	hsa-mir-7<br>hsa-mir-372<br>hsa-mir-373<br>hsa-mir-519a<br>hsa-mir-507<br>hsa-mir-1271<br>hsa-mir-205<br>hsa-mir-22
AL354707.1	Up-regulated gene	hsa-mir-216b
FRMD6-AS2	Down-regulated gene	hsa-mir-9<br>hsa-mir-507<br>hsa-mir-1271<br>hsa-mir-143<br>hsa-mir-4770<br>hsa-mir-182<br>hsa-mir-218<br>hsa-mir-22<br>hsa-mir-338<br>hsa-mir-128
LINC00520	Up-regulated gene	hsa-mir-372<br>hsa-mir-373<br>hsa-mir-519a<br>hsa-mir-195<br>hsa-mir-519d<br>hsa-mir-98<br>hsa-mir-4458<br>hsa-mir-4500<br>hsa-mir-205<br>hsa-mir-216b

		hsa-mir-223
LINC00261	Up-regulated gene	hsa-mir-301b<br>hsa-mir-454<br>hsa-mir-4295<br>hsa-mir-3666<br>hsa-mir-212<br>hsa-mir-143<br>hsa-mir-4770<br>hsa-mir-152<br>hsa-mir-153<br>hsa-mir-155<br>hsa-mir-182<br>hsa-mir-429<br>hsa-mir-548a<br>hsa-mir-216b<br>hsa-mir-218<br>hsa-mir-508<br>hsa-mir-223<br>hsa-mir-338<br>hsa-mir-128
